# Fast and Slow Signal Propagation in Abiotic Polypeptide Assemblies

**DOI:** 10.1002/cbic.202500943

**Published:** 2026-03-23

**Authors:** Panagiotis Mougkogiannis, Andrew Adamatzky

**Affiliations:** ^1^ Unconventional Computing Laboratory University of the West of England Bristol UK

**Keywords:** catalytic assistance, information‐guided organization, proteinoids, template‐directed assembly, unconscious

## Abstract

Proteinoid microspheres—abiotically synthesized by thermal polymerization of amino acids—exhibit spontaneous electrical potential fluctuations despite lacking genetic material, membranes, or ion channels. Here, we quantify the electrical and structural dynamics of five proteinoid compositions using multi‐electrode differential recordings and high‐resolution electron microscopy. The assemblies display rapid voltage oscillations (timescales ≪1 min), long‐timescale drifts (hours to days), exponential relaxation, and correlated potential shifts across spatially separated electrode pairs (Pearson correlations 0.147–0.601, significantly above baseline noise levels, r<0.05), suggesting composition‐dependent patterns of electrical coupling. Optical stimulation induces reproducible voltage responses characterized by logarithmic drift and stimulus‐specific stabilization, indicating that proteinoid networks can modulate electrical pathways in response to external perturbations. Morphological analysis reveals that single‐amino‐acid systems create uniform microspheres (2–3 μm). In contrast, mixed compositions lead to varied structures. These include hollow spheres, lamellar extensions, and crystalline aggregates that can reach 129 μm. Each structure shows unique electrical signatures. We develop a quantitative framework based on algorithmic complexity (Lempel–Ziv), spatial coherence (phase‐locking value), and graph‐theoretical metrics (global efficiency, clustering coefficient) to characterize emergent dynamics in these abiotic networks. These results show that proteinoid assemblies have unique electrical properties based on their composition. This may help us understand prebiotic organization and inspire new types of bioinspired computing materials.

## Graphical abstract

Proteinoid microspheres formed by thermal polymerization of amino acids exhibit spontaneous electrical potential fluctuations without genetic material, membranes, or ion channels. Multi‐electrode recordings and electron microscopy reveal composition‐dependent voltage oscillations, long‐term drifts, and correlated signals across electrodes. Optical stimulation produces reproducible responses and stabilization. Diverse microsphere morphologies show distinct electrical signatures, supporting emergent electrical coupling in abiotic networks.

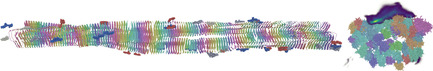

## 1 Introduction

Proteinoids are complexes of amino acids formed by heat [1, 2, 3, 4, 5, 6, 7, 8]. Their self‐assembly enables experimental investigation of emergent behaviors that exhibit functional characteristics analogous to distributed information processing in living systems [9].In this study, we look at proteinoid microspheres as models for the dual‐process framework of fast and slow thinking proposed in [10, 11, 12]. System 1 stands for quick, automatic responses. System 2 refers to slower, thoughtful regulation. We aim to understand the electrical and structural dynamics of these synthetic networks. We will map their spontaneous, or intuitive, reactivity (System 1) and adaptive behavior (System 2). This relates to traits such as signal transduction, latency, and pathway stability. We study multichannel electrical responses to stimuli. This helps us to understand how proteinoid self‐assembly creates complex bioinspired functions [13]. These functions include proto‐computational abilities and adaptive signal processing [14]. We emphasize that this cognitive analogy is functional rather than mechanistic—proteinoids do not “think,” but they exhibit dual timescales in their electrical dynamics that operationally resemble fast and slow information processing. This framework is supported by three observations. First, multi‐electrode recordings (Pt/Ir pairs, 10 mm separation, 1 Hz sampling) reveal a clear separation of timescales between rapid voltage transients (Δt<10 min, corresponding to frequencies of 0.001–0.01 Hz) and slow drifts (Δt>1 h, with plateaus exceeding 10 h) that cannot be captured by single‐timescale models. Second, these behaviors map onto reflexive versus adaptive responses observed in a wide range of physical systems, from colloidal assemblies to neural networks [15, 16]. Third, the data require clear definitions: Signal transduction is quantified through stimulus–response correlations significantly above baseline noise, latency through response timing in the fast regime, and pathway stability through mathematical characterization of voltage evolution patterns. Different compositions have unique electrical signatures. Some show logarithmic drift over time, while others have quick transitions across wide voltage ranges. These observations support our dual‐regime framework. This study aims to connect synthetic biomaterials and cognitive models [17]. The goal is to improve our understanding of prebiotic systems [18]. We also explore their uses in unconventional computing [19] and synthetic biology [20].

The search for the origin of life [21] has focused on prebiotic systems—chemical environments capable of generating life's building blocks under early Earth conditions. The 1953 Miller–Urey experiment [22] demonstrated that amino acids, the fundamental building blocks of proteins, can be synthesized abiotically under plausible prebiotic conditions. They did this using simple gases like methane, ammonia, water vapor, and hydrogen under electrical discharge. This supported Oparin's “primordial soup” idea [23, 24]. In the 1950s and 1960s, Sidney Fox created proteinoids by heating amino acids. He suggested these as models for protocells [25]. Fox noted that proteinoid microspheres had structural traits like selective permeability and catalytic activity. They also showed basic electrical responses. This discovery was largely ignored because later research centered on the RNA world hypothesis [26, 27]. The renewed interest in proteinoids comes from progress in unconventional computing and synthetic biology [14]. This drives our detailed study of their electrical dynamics. We see them as possible materials for abiotic information processing.

Proteinoids can mimic the functions of biological polymers, even if they are made artificially. This establishes a connection between abiotic chemistry and biological systems. Fox's experiments found that heating dry amino acid mixtures above 100∘C resulted in branched, protein‐like structures. Upon cooling in water, these structures formed microspheres. These microspheres exhibit behaviors such as budding, fusion, and responding to stimuli [28], resembling the behaviors of cells. Kaoru Harada contributed to advancements in proteinoid synthesis [29], while Leslie Orgel conducted research on analogous prebiotic polymers [30, 31]. Fox's research focused on the potential of protocell‐like structures [32]. They believed that forming proteinoids was less likely than reactions occurring in water. After 1970, proteinoid research declined as the field prioritized the RNA world hypothesis [26, 27], largely due to proteinoids’ lack of genetic continuity [33, 34]. In the 1980s, molecular biology emerged as a focal point [35], resulting in a reallocation of funding. This relegated proteinoids to a secondary position. Studying these systems provided insights into self‐assembly and prebiotic complexity [36]. Yet, recent advances in unconventional computing and synthetic biology have renewed interest in proteinoids not as complete protocells, but as abiotic substrates for information processing [13, 19]. Modern multi‐electrode recording techniques and quantitative network analysis let us study electrical dynamics in detail. This was only seen qualitatively in Fox's original work. Now, we can rigorously investigate how different compositions affect signal processing for the first time [6, 37].

Intelligence in biological systems [38] extends beyond the confines of the brain. It manifests in various forms, including plants [39, 40], fungi [41], kombucha cultures [42, 43, 44, 45, 46, 47], and algae [48]. The use of terms like ”intelligence” for these systems is debated. Yet, these organisms show clear signs of electrical signaling, adaptive responses, and information sharing through networks. These are measurable facts that can be studied without needing to interpret them cognitively. This perspective drives our study of electrical dynamics in proteinoid assemblies. Instead of linking these to intelligence or cognition, we focus on measurable electrical behaviors. These include oscillations, correlations, and responses to stimuli. Understanding these behaviors may help model abiotic information processing. These organisms employ decentralized and adaptive methods for information processing. Plants exhibit distributed intelligence via chemical signaling and electrical potentials. The Venus flytrap [49] rapidly closes its trap, while Mimosa pudica [50, 51] exhibits leaf folding behavior. They interact with their environment without relying on a central nervous system. Fungi, especially mycelial networks like *Armillaria* [52, 53], can tackle many problems. Nutrient transport and growth are optimized across extensive regions. This capability has led to the designation “wood wide web” due to its role in facilitating communication among various species [54]. Kombucha comprises a symbiotic culture of bacteria and yeast, referred to as SCOBY [47, 55, 56, 57]. It demonstrates collective behavior in the context of fermentation. The culture modifies its metabolic outputs in response to the availability of nutrients [45].

This suggests a basic form of decision‐making in its microbial community. Algae like *Chlamydomonas* sense light using photoreceptors [58, 59]. This ability helps them move toward light, a process called phototaxis [60]. It's a simple form of sensory processing. These systems use self‐organized networks—like vascular, hyphal, or cellular ones [61]. They can integrate stimuli, adapt to change, and show memory‐like responses. This suggests that intelligence, or the ability to process and act on information, likely came before animal cognition. It might even trace back to prebiotic forms like proteinoids. This is where basic responsiveness and structural complexity first emerged.

The proteinoids’ varied structure allows different signals to spread. This is like a decentralized decision‐making system, like proto‐intelligence. It acts as a precursor to cognitive function in living systems. Their reactions to repeated stimuli suggest proto‐consciousness. This means they have a basic awareness. They can take in environmental information and turn it into organized responses. Proteinoids show us how proto‐intelligence and proto‐consciousness could arise. They do this not through neurons but by using molecular complexity and self‐assembly. This offers a link between nonliving chemistry and the start of biological thinking. Proteinoids serve as prebiotic models. They show that proto‐intelligence and proto‐consciousness can arise from molecular complexity and self‐assembly. This idea connects abiotic chemistry to the beginnings of biological cognition. This new ability shows that proteinoids might handle basic information processing. Their molecular interactions create patterns like decision‐making or sensing the environment. As these synthetic systems change in different conditions, they can reorganize and respond. This ability supports the idea of a proto‐conscious state. It may also help create the advanced intelligence found in later biological beings. We study these properties to uncover how chemicals relate to cognition. We suggest that the complexity in proteinoids shows how abiotic polypeptide assemblies can have coordinated dynamics based on their composition. These properties might help us understand prebiotic organization and the rise of basic information processing.

### 1.1 Theoretical Framework and Quantitative Metrics for Emergent Dynamics

Our quantitative analysis is motivated by the ecological approach to intelligence [62, 63], which interprets adaptive behavior as emerging from physical principles rather than requiring neural computation. This framework has been applied to both living systems (slime molds navigating environments [64, 65, 66, 67], *Physarum polycephalum* [68]) and nonliving dissipative structures (Rayleigh‐Bénard convection cells [69, 70]), where organized behavior arises from nonequilibrium thermodynamics and autocatakinetic processes [62, 71]. Under this view, ”intelligence” is not exclusive to brains but characterizes any system that processes environmental information to guide adaptive responses through energy dissipation [72]. This perspective reframes information not as Shannon uncertainty reduction [73] but as Gibson information [62, 74]—environmental specifications that afford action possibilities at appropriate spatiotemporal scales. For proteinoid networks, electrical potentials across electrode channels show information states. These states indicate how the system responds to optical stimuli. Instead of cognitive representations, we see channel‐specific voltage patterns.

Evaluating proteinoid networks needs metrics. These metrics measure how well they process environmental information without using neural representations. To clearly define these emergent behaviors, we shift from qualitative comparisons to quantitative measures. We focus on nonlinear dynamics and information theory. We examine how proteinoids show spatial coherence (coordination) and temporal complexity (information density). These metrics match the ecological approach to physical intelligence. They view the material as a thermodynamic system. This system reduces uncertainty by being more specific [62]. We suggest a mathematical framework to measure these behaviors. It is meant to be used directly on the recorded voltage time‐series data.

#### 1.1.1 Spatial Coherence Index (I_sync_)

To quantify whether proteinoid networks exhibit coordinated or independent channel dynamics, we compute the phase locking value (PLV) between all electrode pairs [75]. This metric captures functional connectivity, defined as the degree to which spatially separated channels maintain consistent phase relationships over time, thereby replacing qualitative descriptions of spatial heterogeneity with a quantitative measure of temporal coordination.

Prior to synchronization analysis, several preprocessing steps were applied to minimize potential confounds. First, a high‐pass filter with a cutoff frequency of 0.0001 Hz was used to remove slow baseline drift and DC offsets that could artificially inflate correlation measures. Second, stimulus‐locked transients were excluded by removing the first 10 s following each optical pulse. Third, synchronization patterns were verified to persist across non‐overlapping time windows, ruling out artifacts arising from transient common‐mode signals.

The spatial coherence index is computed as

(1) $$I_{\mathrm{sync}}=\frac{1}{N}\sum_{j=1}^{N}\left|\frac{1}{T}\int_{0}^{T}e^{i\left(\phi_{x}(t)-\phi_{y}(t)\right)}\;dt\right|$$

where the time integration yields the complex‐valued average phase difference for each channel pair and the magnitude operator is applied to obtain the PLV for that pair. The final value is obtained by averaging across all N unique channel pairs. This order of operations ensures that $0\leq I_{\mathrm{sync}}\leq 1$, with values approaching unity indicating persistent phase locking (coordinated dynamics) and values near zero indicating phase independence (uncorrelated dynamics).

In this formulation, $\phi_{x}(t)$ and $\phi_{y}(t)$ denote the instantaneous phases of signals recorded from two distinct electrode channels, extracted using the Hilbert transform. The parameter N represents the number of unique channel pairs analyzed; for an eight‐channel system, N=(82)=28. The variable T denotes the total duration of the recording, expressed in samples at a sampling rate of 1 kHz. Elevated values of $I_{\mathrm{sync}}$ indicate that phase relationships between channels cannot be attributed to random noise or measurement artifacts, but instead reflect genuine spatiotemporal coordination in the electrical dynamics. Such coordinated activity is operationally analogous to distributed signal propagation observed in decentralized biological networks, such as slime mold colonies [76], although the underlying physical mechanisms differ fundamentally [76].

#### 1.1.2 Algorithmic Complexity (*C*_LZ_)

To quantify the ”richness” of the proteinoid response, we employ Lempel‐Ziv Complexity. This metric distinguishes between simple repetitive spiking (reflexive) and complex, nonrepeating patterns (adaptive) [77].

(2) $$C_{LZ}=\frac{c(n)}{\frac{n}{\log_{2}n}}$$

$C_{LZ}$ is the normalized Lempel–Ziv complexity.

c(n) is the number of unique substrings (patterns) detected in the binary spike train sequence.

n is the length of the time series.

A higher $C_{LZ}$ means the proteinoid network creates detailed responses. It integrates environmental input over time instead of just reacting with a fixed reflex.

### 1.2 Topological Quantification of Network States

We investigate the organizational principles of proteinoid electrical networks using a graph‐theoretical framework. Our analysis focuses on three key properties: signal complexity, which quantifies the unpredictability of temporal activity patterns; global integration, which evaluates how efficiently information is communicated across the entire network; and local segregation, which assesses the presence of modular substructures within the network. This approach is inspired by the Integrated Information Theory (IIT) framework [78, 79, 80, 81], which posits that systems exhibiting both high differentiation (segregation) and high unification (integration) are capable of supporting complex information processing. We emphasize that the use of these metrics does not imply or assert consciousness in proteinoid systems. Rather, IIT‐inspired topological analysis provides a rigorous and quantitative methodology for characterizing network organization in physical systems [81].

#### 1.2.1 Permutation Entropy ($H_{\mathrm{PE}}$): Complexity

To quantify the temporal complexity of the electrical dynamics, we compute the Permutation Entropy [82]. The permutation entropy $H_{\mathrm{PE}}$ differs fundamentally from standard Shannon entropy. Whereas Shannon entropy is based on amplitude distributions, $H_{\mathrm{PE}}$ characterizes the ordering of values within a time series. This approach emphasizes the diversity and predictability of temporal patterns rather than absolute signal magnitude. Permutation entropy is robust to amplitude noise and outliers, as it operates on rank order rather than raw values. However, it remains sensitive to methodological choices, particularly the embedding dimension and the sampling rate, which must be selected carefully to ensure meaningful interpretation [82].

(3) $$H_{\mathrm{PE}}(m)=-\frac{1}{\log_{2}(m!)}\sum_{j=1}^{m!}p_{j}\log_{2}p_{j}$$

m is the embedding dimension (order), defining the length of the ordinal patterns analyzed (set to m=3).

$p_{j}$ is the relative frequency of the j‐th unique permutation pattern occurring in the time‐series.

Normalization: The value is normalized by $\log_{2}(m!)$ to range between 0 (perfectly monotonic/predictable) and 1 (stochastic/random).

The parameter m denotes the embedding dimension, which defines the length of the ordinal patterns used in the permutation entropy calculation. In this study, we set m=3 following standard practice for noisy physiological and physical signals [82]. This choice provides a balanced trade‐off between statistical reliability and discriminative power. For m=3, there are 3!=6 possible ordinal patterns, which is sufficient to capture meaningful temporal structure without requiring excessively long data segments. This embedding dimension requires only four consecutive samples per pattern, making it well suited to our 1 kHz sampling rate and enabling efficient computation across approximately 4,300 analysis windows. Larger embedding dimensions (m≥5) would require substantially longer stationary segments, which are not compatible with the inherently nonstationary nature of proteinoid electrical activity. The quantity $p_{j}$ represents the relative frequency of the j‐th unique permutation pattern observed within each analysis window. The permutation entropy is normalized by $\log_{2}(m!)$ so that its values range from 0, corresponding to perfectly monotonic and fully predictable dynamics, to 1, corresponding to maximally irregular behavior approaching that of a uniformly random process. Higher values of $H_{\mathrm{PE}}$ indicate that the system generates a broad diversity of temporal patterns, reflecting complex and nonrepetitive dynamics. Conversely, lower values correspond to more structured and regular oscillatory behavior with limited pattern diversity [83]. In this context, elevated permutation entropy is interpreted as evidence of rich state‐space exploration. This interpretation does not imply deterministic chaos, which would require additional analyses such as Lyapunov exponent estimation or attractor reconstruction.

#### 1.2.2 Global Efficiency (*E* *_glob_*): Integration

We measure how well signals spread across the network. We do this by calculating the global efficiency of the functional graph G, which is based on phase‐synchronization relationships. Integration means how well electrical phase information moves between distant electrode channels. This happens through both direct and indirect coupling pathways. The graph is constructed independently for each analysis window.

Each network node corresponds to one of the eight electrode channels (ChA–ChH). For every unique channel pair ((82)=28 pairs), the Phase Locking Value (PLV) is computed to form a weighted connectivity matrix W with elements $W_{ij}\in [0,1]$. To obtain a binary adjacency matrix A, an adaptive thresholding procedure is applied such that $A_{ij}=1$ when $W_{ij}>\theta_{w}$ and $A_{ij}=0$ otherwise. The threshold $\theta_{w}$ is defined as the 75th percentile of all PLV values within the current analysis window, ensuring a consistent network density of approximately 25% edge retention across windows despite variations in signal amplitude. The robustness of this procedure was verified by testing threshold values in the 70th–80th percentile range and by comparing the resulting patterns with weighted efficiency metrics, confirming that the observed efficiency trends are not artifacts of binarization.

Global efficiency is then defined as

(4) $$E_{\mathrm{glob}}=\frac{1}{N(N-1)}\sum_{i\neq j\in G}\frac{1}{d_{ij}},$$

where N is the total number of nodes (N=8) and $d_{ij}$ denotes the shortest path length, measured as the minimum number of edges connecting node i to node j in the binary adjacency matrix A. Shortest paths are computed using a breadth‐first search algorithm. For node pairs that are not connected, $d_{ij}$ is set to infinity, such that $1/d_{ij}=0$, following standard convention [84].

High values of $E_{\mathrm{glob}}$ approaching unity indicate a small‐world‐like topology [76], in which most node pairs are connected by short paths (typically $d_{ij}\leq 2$), enabling rapid signal propagation across the entire network. Conversely, low values of $E_{\mathrm{glob}}<0.3$ indicate fragmented or lattice‐like organization with limited long‐range connectivity. In the context of proteinoid assemblies, elevated global efficiency implies that locally established electrical phase coherence can propagate throughout the network via intermediate channels, supporting coordinated responses across spatially distributed microsphere populations.

#### 1.2.3 Clustering Coefficient (*C*_glob_): Segregation

We calculate the mean Local Clustering Coefficient [76] to check how the network tends to form close‐knit groups and specialized substructures. This metric quantifies the prevalence of triangles (closed triplets) in the network, capturing the extent to which neighboring nodes of a given node are also connected to each other. High local clustering is not the same as formal modularity. Formal modularity needs community detection algorithms. However, high local clustering can indicate functional segregation. This is true in networks where dense local neighborhoods work semi‐independently.

Specifically, we compute the nodewise local clustering coefficient for each node ii i, then average across all nodes:

(5) $$C_{glob}=\frac{1}{N}\sum_{i=1}^{N}C_{i}=\frac{1}{N}\sum_{i=1}^{N}\frac{2t_{i}}{k_{i}(k_{i}-1)}$$

Here, $C_{i}$ denotes the local clustering coefficient of node i. We emphasize that this definition corresponds to the mean local clustering coefficient, also known as the Watts–Strogatz clustering measure [76], rather than the global transitivity ratio defined as the number of triangles divided by the number of connected triples. This formulation was selected because it assigns equal weight to all nodes regardless of degree, thereby avoiding biases toward high‐degree hubs that can disproportionately influence transitivity‐based measures. For nodes with degree $k_{i}<2$, the clustering coefficient is defined as $C_{i}=0$ by convention. In this context, $C_{i}$ represents the fraction of possible triangles that actually exist among the neighbors of node i, $t_{i}$ denotes the number of triangles (closed triplets of edges) involving node i, and $k_{i}$ is the degree of node i in the binary adjacency matrix. The total number of nodes in the network is N=8. High values of the global clustering coefficient $C_{\mathrm{glob}}$, close to one, show that the network has tightly connected local groups. In these groups, neighboring nodes often connect to each other, creating clique‐like structures. This organization helps functional segregation. It allows specialized processing in tightly linked subgroups [85]. In proteinoid networks, high clustering means nearby electrode channels work well together. This creates local functional modules. High clustering with high global efficiency shows a small‐world organization [76, 86]. This means it has efficient global connections and strong local specialization. This pattern is common in biological neural networks [87].

### 1.3 Dual‐Regime Dynamics: Operational Classification

We classify how the proteinoid network behaves electrically in two ways. This is based on a detailed look at voltage time‐series features like relaxation timescales and stimulus–response latencies. This classification is empirical and data‐driven, not analogous to psychological constructs [12], though we note functional parallels for conceptual framing.

For each channel and recording segment, the analysis proceeds as follows. The raw voltage traces are first detrended using high‐pass filtering with a cutoff frequency of 0.0001 Hz to remove slow baseline drift and isolate transient dynamics. Local voltage maxima are then identified as peaks exceeding two standard deviations above a moving average computed over a 10,000‐sample window. For each detected peak, the subsequent 1,000‐sample (1 s) decay segment is extracted and fitted using both exponential models, $V(t)=V_{0}e^{-t/\tau }$, and logarithmic models, $V(t)=V_{0}+A\log(t)$, via least‐squares regression. Characteristic timescales are obtained from the exponential decay constant τ or, for logarithmic fits, from the characteristic time $t_{c}=e^{-V_{0}/A}$, with model selection based on goodness of fit ($R^{2}$). Segments with τ<600 s (10 min) are assigned to Regime I, whereas those with τ>3600 s (1 h) are assigned to Regime II. Intermediate timescales between 600 and 3600 s are considered transitional and excluded from regime‐specific analyses.

Regime I corresponds to volatile dynamics characterized by rapid voltage transients and short relaxation timescales (τ<10 min). The rate of voltage change, ΔV/Δt, is computed as the maximum absolute slope within 100‐sample (0.1 s) sliding windows applied to the raw voltage trace sampled at 1 kHz. Slopes are estimated using a five‐point Savitzky–Golay filter of polynomial order 2 to suppress noise while preserving sharp transitions. For example, L‐Phe:L‐Lys systems exhibit maximum transient slopes of ΔV/Δt≈1.129 mV s^−1^ during stimulus‐evoked responses. Regime I dynamics show high‐frequency spectral content. Most power lies in the 0.001–0.01 Hz range. They also have short stimulus–response latencies, under 10 s. Additionally, rapid autocorrelation decay happens in less than 100 s. These properties show that signals move quickly. Yet, the exact mechanisms, like ionic currents, capacitive coupling, or conformational changes, still need to be explained. Regime II corresponds to nonvolatile dynamics characterized by slow voltage evolution over extended timescales (τ>1 h). After removing Regime I transients using median filtering with a 10,000‐sample window, baseline drift is quantified by fitting voltage traces over contiguous 10,000‐s segments to logarithmic models, $V(t)=V_{0}+A\log(t/t_{0})$, or exponential models, $V(t)=V_{1}+Be^{-t/\tau_{2}}$. Model selection is determined using the Akaike Information Criterion (AIC). As an illustrative example, L‐Phe Channel C exhibits logarithmic drift well described by $V(t)\approx -40+15\log(t/10^{4})$ mV for t>40,000 s, with an AIC of −1247 compared to −1198 for the exponential model, indicating a sustained, memory‐like electrical state. Regime II is defined by a few key features. First, there is a dominant low‐frequency drift lasting over 1 h. Second, voltage plateaus last more than 10 h without stimulation. Finally, there is hysteresis in stimulus–response curves, meaning post‐stimulus voltages differ from pre‐stimulus baselines. These features suggest slow charge redistribution, structural changes, or stable reconfigurations. Yet, the exact causes are still unclear. The shift from exponential relaxation in Regime I to logarithmic drift in Regime II shows a clear difference in timescales. This change occurs across various compositions. This dual‐timescale behavior is common in glassy systems, colloidal suspensions, and biological tissues. It comes from complex energy landscapes that have many relaxation pathways. Table 1 shows how measured electrical behaviors, like spontaneous oscillations, threshold responses, and synchronization patterns, relate to thermodynamic and network processes. The terms “memory‐like” and “adaptive” are just operational. They refer to persistence and stimulus dependence in the signals we measure. They do not suggest any cognitive or neural processes.

**TABLE 1:** Mapping Cognitive Heuristics to Biophysical Dynamics in Proteinoid Microspheres.

Cognitive Parallel (System 1)	Biophysical Mechanism (Regime I)
**Automaticity**: Rapid, intuitive response to stimuli without conscious processing.	**Low‐Latency Transduction**: Immediate generation of transient voltage spikes upon square‐wave stimulation, bypassing slow capacitive charging phases.
**Pattern Matching**: Converting initial perceptions into convictions/beliefs.	**Attractor Formation**: Transition from volatile, chaotic oscillations to stable limit cycles (steady‐state response) under repetitive stimulation.
**Attention Guidance**: Focusing on specific signals based on intensity or relevance.	**Threshold Gating**: Non‐linear voltage response where only inputs exceeding a specific potential (or light intensity) trigger a global depolarization event.
**Skill Acquisition**: Developing intuitive expertise through repetition.	**Memristive Plasticity**: Hysteresis effects where prior electrical activity lowers the resistance for future signals, creating ”learned” conduction pathways.
**Associative Memory**: Constructing a unified network of linked concepts.	**Spatial Synchronization**: Emergence of high synchronization indices (*I*_sync_) between distant channels, creating a coupled functional network.
**Cognitive Ease**: Linking simplicity to validity and lower alertness.	**Signal‐to‐Noise Optimization**: The system naturally relaxes into low‐energy states (stable baselines) that resist minor thermal or voltage fluctuations.
**Anomaly Detection**: Differentiating the unexpected from the routine.	**High‐Pass Filtering**: Rapid, high‐amplitude spiking in response to sudden voltage shifts (step functions) while dampening slow, DC‐like inputs.
**Generative Modeling**: Deducing explanations or filling in gaps.	**Self‐Organized Criticality**: Spontaneous generation of complex oscillatory bursts (resembling neural avalanches) even in the absence of continuous external forcing.
**Bias/Heuristics**: Overlooking uncertainty to favor a dominant conclusion.	**Winner‐Takes‐All Dynamics**: Current preferentially flows through the path of least resistance, effectively “ignoring” high‐resistance (ambiguous) regions of the network.

## 2 Experimental

### 2.1 Multichannel Differential Recording Apparatus for Characterizing Spontaneous and Evoked Electrical Activity in Self‐Assembled Proteinoid Systems

All chemicals for the synthesis of proteinoid microspheres were purchased from Sigma–Aldrich (Merck) and used as received without further purification. The amino acids employed included L‐Phenylalanine (reagent grade, ≥98%, CAS. No. 63‐91‐2), L‐Aspartic acid (reagent grade, ≥98%, CAS. No. 56‐84‐8), L‐Glutamic acid (ReagentPlus, ≥99%, CAS. No. 56‐86‐0), L‐Lysine (≥98%, CAS. No. 56‐87‐1), and L‐Arginine (≥98%, CAS. No. 74‐79‐3). These precursors were selected to ensure consistent polymerization kinetics and reproducible electrical properties across all experimental trials. This made sure that the proteinoid self‐assembly process could be repeated. It also kept the electrical properties consistent in all trials. We recently investigated the influence of water on proteinoid polymerization [88]. The introduction of water during thermal polymerization was found to significantly affect both the structure and morphology of the resulting proteinoid assemblies. In particular, controlled hydration during synthesis modulates the degree of cross‐linking and promotes the formation of hierarchical architectures. In the present study, all proteinoid samples were synthesized under anhydrous conditions by heating amino acid mixtures above 100^∘^C [89]. This approach ensured high reproducibility and allowed self‐assembly behaviors to be decoupled from hydration effects. Water was introduced only during the microsphere formation stage, upon cooling in aqueous solution, where it facilitates spontaneous morphological assembly. This two‐step procedure—anhydrous polymerization followed by aqueous self‐assembly—clearly separates polymer formation from structural organization, enabling a more precise investigation of structure–function relationships underlying the observed electrical dynamics.

This study examined amino acid compositions (Figure 2) based on three primary criteria: diversity in physicochemical properties to enable a broad functional range, general prebiotic relevance supported by Miller–Urey‐type synthesis yields and early screening results indicating distinct electrical signatures [22]. We did not test all possible combinations of the 20 proteinogenic amino acids, as a comprehensive survey would require analysis of 20 single–amino‐acid systems, (202)=190 dipeptide combinations, and (203)=1140 tripeptide combinations, corresponding to more than 1,350 total formulations. Instead, we employed a targeted approach based on functional group diversity. While acidic residues like L‐aspartic acid and L‐glutamic acid are readily produced in Miller–Urey‐type synthesis [22], other amino acids were selected to expand the chemical space. L‐phenylalanine (L‐Phe) was chosen as a typical hydrophobic, aromatic amino acid. Its benzyl side chain contains π‐electron systems, which may play a role in charge transport and photosensitivity. L‐aspartic acid (L‐Asp) was selected because of its acidic, negatively charged carboxyl group, which enables electrostatic interactions and proton‐transfer pathways. L‐lysine (L‐Lys) possesses a basic, positively charged amine group, providing electrostatic complementarity to acidic residues. L‐glutamic acid (L‐Glu), chemically similar to L‐Asp but with a longer side chain, was included to explore charge‐density and spatial effects. L‐arginine (L‐Arg) was chosen for its guanidinium group, which remains protonated over a wide pH range and can participate in multiple hydrogen‐bonding interactions. Binary combinations (L‐Phe:L‐Lys and L‐Glu:L‐Arg) were designed to form amphiphilic systems containing both hydrophobic and charged domains. This design was expected to enhance self‐assembly complexity and improve electrical responsiveness. The tricomponent system (L‐Glu:L‐Phe:L‐Asp) was selected to incorporate aromatic, acidic, and structural diversity simultaneously, based on previous studies showing enhanced morphological polymorphism in multicomponent proteinoids [88]. For the synthesis of dipeptide and tripeptide proteinoids, equimolar ratios of the constituent amino acids were employed. The amino acids were thoroughly mixed in the dry state prior to thermal polymerization to promote random copolymerization rather than sequential block formation. L‐Phe:L‐Lys proteinoids were synthesized by combining equal amounts of L‐phenylalanine and L‐lysine, heating the mixture to 180–200∘C for 3–6 h under a nitrogen atmosphere to prevent oxidation, followed by cooling and dissolution in deionized water to induce microsphere formation. The resulting proteinoids are statistical copolymers with heterogeneous sequence distributions, rather than well‐defined dipeptides or tripeptides in the strict chemical sense. Accordingly, the notation “L‐Phe:L‐Lys” refers to amino acid composition rather than a specific peptide sequence (Table 2).

**TABLE 2:** Proteinoid compositions investigated and selection basis.

Composition	Functional Groups	Selection Basis
L‐Phe	Aromatic, hydrophobic	π‐electron system, photosensitivity
L‐Asp	Acidic, negatively charged	Proton transfer, electrostatic interactions
L‐Phe:L‐Lys	Aromatic + basic	Amphiphilic, charge complementarity
L‐Glu:L‐Arg	Acidic + basic	Strong ionic pairing, hydrogen bonding
L‐Glu:L‐Phe:L‐Asp	Acidic + aromatic	Maximal chemical diversity, enhanced morphological complexity

**FIGURE 1:**
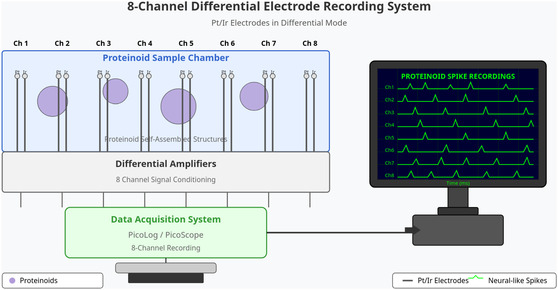
Multichannel recording system to detect electrical activity in proteinoid microspheres. The device uses eight pairs of platinum/iridium (Pt/Ir) electrodes. They are in differential mode to detect electrical signals from proteinoid self‐assembled structures. Each electrode pair is placed to record from different areas in the sample chamber. This setup helps map the electrical activity patterns across the proteinoid field. Signals from the electrodes go through differential amplifiers. This boosts the signal‐to‐noise ratio and cuts out common‐mode interference. The processed signals are digitized using a PicoLog/PicoScope data acquisition system. This system has 8‐channel capability. The results show spike waveforms on a computer monitor, resembling neural activity. This setup allows us to see and record electrical oscillations from proteinoid self‐assembly. It shows the electrical properties in these early life structures.

Figure 1 shows how we recorded electrical activity in proteinoid self‐assembled structures. The recording system has eight pairs of platinum/iridium (Pt/Ir) electrodes. Each electrode is 0.1 mm in diameter. They are set up in differential mode, with a fixed distance of 10 mm between electrodes. This setup covers the space in the proteinoid sample chamber. It also reduces common‐mode interference. Each pair of electrodes acts as its own recording channel. This setup enables simultaneous multisite measurements throughout the proteinoid field.

Signal acquisition used two systems: (1) a PicoLog data logger to monitor spontaneous oscillations from proteinoid structures continuously and (2) a PicoScope oscilloscope for high‐resolution recordings that synced with optical stimulation. This dual‐acquisition method let us examine the intrinsic electrical properties and the stimulus–response relationships in the proteinoid system. The differential recording setup used well‐spaced electrode pairs. This improved the signal‐to‐noise ratio, which is key for picking up the low‐amplitude electrical signals found in these prebiotic structures. Real‐time visualization showed neural‐like spike activity across all eight channels. This helped map electrical phenomena during the experimental sessions.

### 2.2 Computational Quantification of Emergent Dynamics

We employed a sliding‐window analysis to distinguish the unstable, high‐variability signals characteristic of Regime I from the stable, memory‐like structures characteristic of Regime II in the multichannel voltage recordings. Each window contained N=1000 samples and advanced with a step size of 100 samples, allowing high‐granularity tracking of temporal evolution.

All recordings were acquired at a 1 kHz sampling rate, yielding 1 s analysis windows with 0.1 s advancement steps and 90% overlap between consecutive windows. Regime classification is performed using two operational criteria computed for each window: the voltage standard deviation (*σ*_V_) and the autocorrelation decay time ($\tau_{\mathrm{AC}}$), defined as the lag at which the autocorrelation function decays to 1/e. Windows are assigned to Regime I (volatile) if *σ*_V_ exceeds the 60th‐percentile threshold and $\tau_{\mathrm{AC}}<0.1$ s, indicating rapid signal fluctuations. Regime II (nonvolatile) classification requires sustained low variability (*σ*_V_ below threshold for >10 h) combined with voltage plateau persistence, defined as a return to within 10% of baseline following stimulus offset—an operational criterion for “memory‐like” behavior. This quantitative assignment rule ensures reproducible regime identification across all compositions and channels.

Algorithmic complexity, denoted *C*_LZ_, was computed to quantify the information density of the proteinoid network. For each time window, the voltage trace V(t) was converted into a binary sequence S(t) by thresholding against the local window mean μ:

$$\begin{array}{c} S(t)=\left\{ \begin{array}{cc} 1, & \text{if}\;V(t)\;>\;\mu ,\\ 0, & \text{otherwise} \end{array}\right. \end{array}$$

The resulting binary sequence was processed using the Lempel–Ziv parsing algorithm, which counts the number of unique substrings required to reconstruct the sequence. The raw complexity count was normalized by the finite‐length upper bound $n/\log_{2}n$ to yield the normalized complexity:

(6) $$C_{\mathrm{LZ}}=\frac{c(n)}{n/\log_{2}n}$$

where c(n) is the Lempel–Ziv complexity of a sequence of length n. Values of $C_{\mathrm{LZ}}\approx 1$ indicate high information content or near‐random behavior, while values approaching 0 indicate highly regular, low‐complexity dynamics.

Spatial coherence was quantified using the Phase Locking Value (PLV), defining the synchronization index $I_{\mathrm{sync}}$. For each channel i, the instantaneous phase $\phi_{i}(t)$ was extracted from the analytic signal obtained via the Hilbert transform. For each channel pair (i,j), we computed:

(7) $$I_{\mathrm{sync}}^{\left(i,j\right)}=\left|\frac{1}{T}\sum_{t=1}^{T}e^{\;i\left(\phi_{i}(t)-\phi_{j}(t)\right)}\right|$$

where T is the number of samples within the window. The PLV ranges from 0 (no consistent phase relationship) to 1 (perfect synchronization). Higher values of $I_{\mathrm{sync}}$ correspond to stronger functional connectivity, reflecting the ability of the material to support collective versus modular processing modes.

## 3 Results and Discussion

### 3.1 Thinking Fast in Proteinoids: Rapid Electrical Signal Propagation and Automatic Response Mechanisms

#### 3.1.1 Volatile Electrical Dynamics and Spontaneous Oscillations in L‐Phe Proteinoids

**FIGURE 2:**
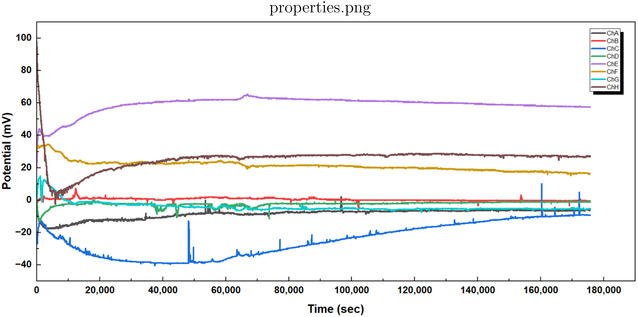
Long‐term recordings capture spontaneous electrical activity in L‐phenylalanine proteinoid networks. These networks show characteristics similar to System 2 thought processing. The graph shows potential measurements from eight channels (ChA to ChH) over about 50 h (or $1.8\cdot 10^{5}$ seconds). This data highlights unique slow–thinking computational traits. Different pathways for processing information have their own baseline potentials and distinct patterns over time. They exhibit rapid changes initially (within 10000 s) and then enter longer periods of stabilization before reaching steady states. Notable features include: (1) Bifurcation into positive (ChE, ChG, ChH) and negative (ChA, ChC) potential domains, suggesting computational polarization. (2) Formation of stable intermediate–term memory in ChE, with a plateau voltage of about 60mV lasting for over 40 h. (3) In ChC, a gradual logarithmic drift is observed, described by the equation $V(t)\approx -40+15\log(\frac{t}{10^{4}})\text{(mV)}\;\text{for }t>4\cdot 10^{4}s$. (4) Sporadic spike events occur in multiple channels, indicating computational threshold‐crossing events. This multichannel activity profile exhibits System 2‐like “thinking slow” traits in proteinoid assemblies. It encompasses: (1) thought processing over extended time periods, (2) parallel but interconnected pathways for computation, (3) formation of long‐term memory (stable potential states), and (4) gradual behavior that aids in optimization. The different pathways taken by similar channels show how they become specialized—a key feature of complex adaptive systems. These findings demonstrate that L‐Phe proteinoid networks are capable of both rapid and slow signal processing, which may reflect a basic form of the dual‐process system found in more advanced cognitive systems.

Our recordings show that L‐phenylalanine proteinoid networks act in two unique ways in their electrical behavior. They display traits of both slow, thoughtful processing and quick, intuitive responses. Figure 2 shows that the long‐term potential changes in a complex way. We can model this process by mixing logarithmic drift with exponential stabilization.

(8) $$V_{\mathrm{slow}}(t)=\left\{ \begin{array}{cc} V_{0}+A\cdot e^{-t/\tau_{1}} & \text{for}\;t\;<\;t_{\mathrm{transition}}\\ V_{1}+B\cdot \log\left(\frac{t}{t_{0}}\right) & \text{for}\;t\geq t_{\mathrm{transition}} \end{array}\right.$$

where $V_{0}$ and $V_{1}$ are baseline potentials. A and B are scaling factors. $\tau_{1}$ is the relaxation time constant. *t*_transition_ indicates when the behavior changes from exponential to logarithmic. This math description shows the split into positive and negative potential areas seen across channels. It highlights the key logarithmic drift in ChC that follows:

(9) $$V_{\mathrm{ChC}}(t)\approx -40+15\cdot \log\left(\frac{t}{10^{4}}\right)\text{(mV)}\;\text{for}\;t\;>\;4\cdot 10^{4}\text{ s}$$

**FIGURE 3:**
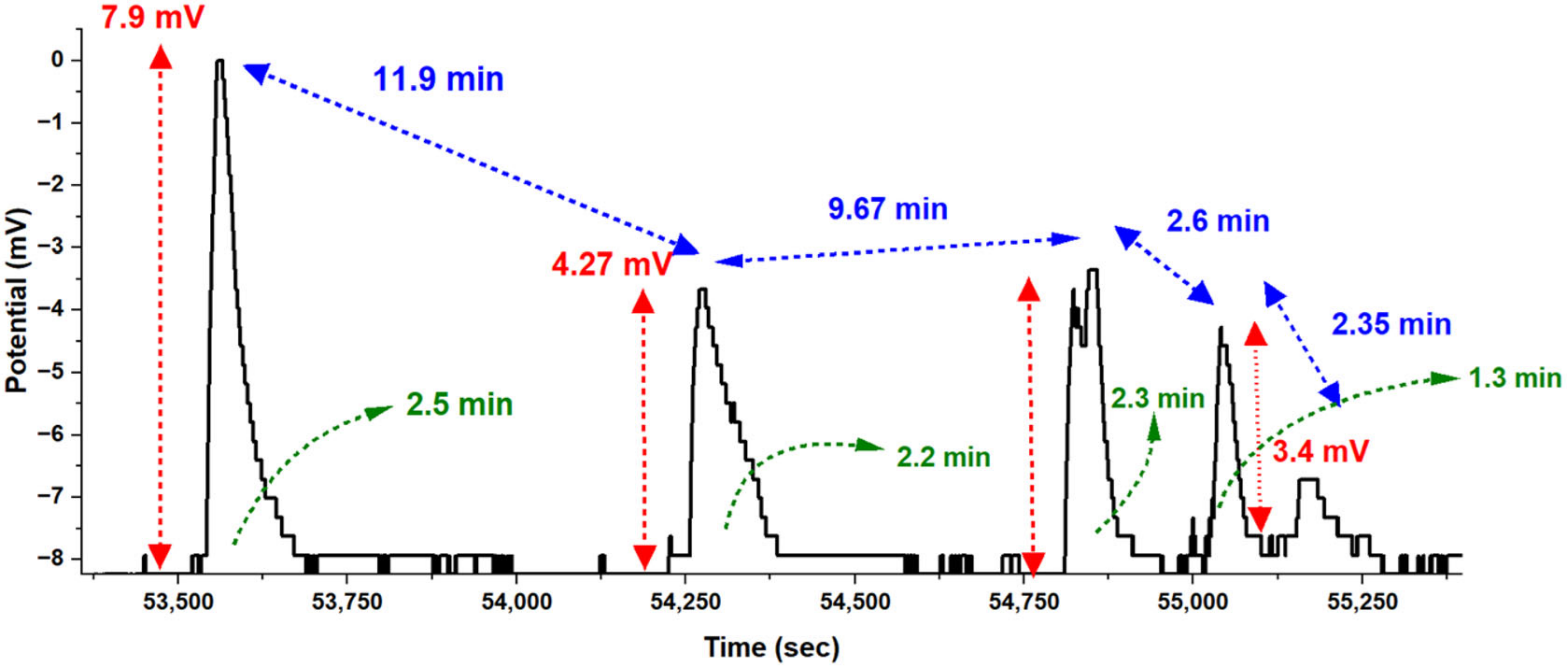
L‐phenylalanine proteinoid microspheres show spontaneous electrical oscillations. These oscillations have emergent computational properties. They work like System 1 thinking, which is known for quick, intuitive responses. The graph shows oscillatory patterns that occur on their own, without outside help. This reveals important details about how information is processed internally. The marked voltage peaks (*V*_max1_ = 7.9, *V*_max2_ = 4.27, *V*_max3_ = 3.4 mV; red triangles) demonstrate amplitude modulation characteristic of self‐regulated signaling networks. Notably, these spontaneous oscillations exhibit precisely timed intervals between major activity bursts (blue arrows, $\Delta t_{\mathrm{interval}1}=11.9$ min, $\Delta t_{\mathrm{interval}2}=9.67$ min, $\Delta t_{\mathrm{interval}3}=2.6$ min, $\Delta t_{\mathrm{interval}4}=2.35$ min) and secondary rhythm patterns (green arrows, $\Delta t_{\mathrm{secondary}1}=2.5$ min, $\Delta t_{\mathrm{secondary}2}=2.2$ min, $\Delta t_{\mathrm{secondary}3}=2.3$ min, $\Delta t_{\mathrm{secondary}4}=1.3$ min). This oscillatory behavior shows that proteinoid structures can process information like System 1. They do this automatically, without needing outside input. This is a key trait of fast‐thinking systems. The progressive shortening of inter‐oscillation periods ($\Delta t_{\mathrm{interval}1}>\Delta t_{\mathrm{interval}2}>\Delta t_{\mathrm{interval}3}$) suggests self‐organizing temporal dynamics with potential adaptive properties. These findings show that simple proteinoid assemblies can create complex behaviors on their own. This might be an early form of molecular cognition that appeared before neural systems.

Figure 3 shows rapid oscillatory patterns. We can see these as bursts with changing amplitudes and shorter gaps between them. The temporal pattern follows:

(10) $$\Delta t_{\mathrm{interval}}(n)=\Delta t_{\mathrm{interval}}(1)\cdot e^{-\alpha (n-1)}$$

where $\Delta t_{\mathrm{interval}}(n)$ is the $n^{th}$ interval between major activity bursts. Here, α is the decay constant that shows how intervals shorten over time. The amplitude modulation follows a decay pattern that is similar.

(11) $$V_{\max}(n)=V_{\max}(1)\cdot e^{-\beta (n-1)}$$

with $V_{\max}(n)$ representing the $n^{th}$ voltage peak amplitude and β controlling the rate of amplitude attenuation.

Slow (Figure 2) and fast (Figure 3) dynamics can occur together in the same proteinoid system. This reveals an important principle. Even at this basic level, these information processing systems split into two modes. This is like the System 1 (fast, intuitive) and System 2 (slow, deliberative) distinction found in cognitive systems.

Heating L‐phenylalanine to its boiling point produces L‐Phe proteinoids. This process promotes random polymerization, resulting in a complex network of peptide bonds that connect phenylalanine residues. L‐phenylalanine is an aromatic amino acid. It has a benzyl side chain (C6H5CH2‐) linked to its chiral carbon. This structure creates a hydrophobic area rich in π‐electrons. These features enable weak interactions, such as π–π stacking and van der Waals forces. In proteinoids, these residues can form irregular, amorphous structures with varying degrees of cross‐linking, creating a mixed matrix. This structural diversity is key to the slow, logarithmic change in electrical potential. The aromatic rings and peptide backbone can trap charge carriers or ions, causing a gradual redistribution over time, which likely results from the initial relaxation of charged species in the network. The relaxation time constant $\tau_{1}$ in Equation (8) indicates how energy is dissipated through hydrogen bonding and hydrophobic interactions within the proteinoid matrix. It should be noted that the observed morphological polymorphism is influenced by both the amino acid composition and the hydration conditions during synthesis, as we have demonstrated elsewhere for Glu–Phe–Asp systems [88]. The present study employs standardized anhydrous synthesis conditions to enable direct comparison across compositions.

The rapid oscillatory patterns show decaying intervals and changing amplitudes.These patterns arise from the flexible structure and ionic conductivity of L‐Phe proteinoids. Benzyl side chains can create areas where electrons move around. This allows for quick charge shifts, leading to bursts of electrical activity. Water molecules or impurities in the proteinoid network can influence these oscillations by acting as dielectric media or charge carriers. This, in turn, helps the network switch quickly between high and low conductance states. The decay constants α and β hint at a self‐regulating mechanism (Equations (10), (11)), possibly due to the slow saturation of charge traps or energy loss through vibrations in the aromatic rings. This matches the quick, intuitive ”System 1” behavior observed. This dual ability—slow thinking and quick reactions—reflects how our minds work and shows the promise of proteinoids as materials that mimic biological systems.

The slow and fast dynamics observed in the L‐Phe proteinoid system likely originate from microdomains within the polymer network. The distribution of these microdomains appears largely stochastic, arising from the thermodynamic self‐assembly processes that occur during thermal polymerization. The formation of microdomains—regions characterized by differing aromatic density, hydration levels, and degrees of cross‐linking—is governed by local variations in temperature, cooling rate, and monomer concentration during synthesis. Although precise synthetic control over microdomain size and spatial distribution has not yet been achieved in the present work, several potential strategies for tuning these architectures merit future investigation. These include the use of controlled cooling protocols with defined temperature gradients to bias nucleation sites, the incorporation of structure‐directing agents or templating molecules during polymerization, sequential polymerization with staged amino acid addition to generate layered architectures, postsynthetic annealing treatments at submelting temperatures to promote domain reorganization, and variation of hydration conditions during the aqueous self‐assembly phase. As demonstrated in our recent work on Glu–Phe–Asp systems [88], water content can significantly modulate structural order. Areas with high aromatic density allow slow charge migration. In contrast, more disordered or hydrated zones support quick ionic or electronic transitions. The thermal synthesis process can create defects or branching points in the polypeptide chain. This boosts the material's memory‐like features, like logarithmic drift, and causes reactive bursts. These traits are like ”System 2” deliberative processing. Phenylalanine residues are hydrophobic. Applying electric fields can cause this property to exhibit semi‐conductive behavior. We see this in the voltage peaks. It suggests that proteinoids might act as a basic model for neural or sensory systems.

**FIGURE 4:**
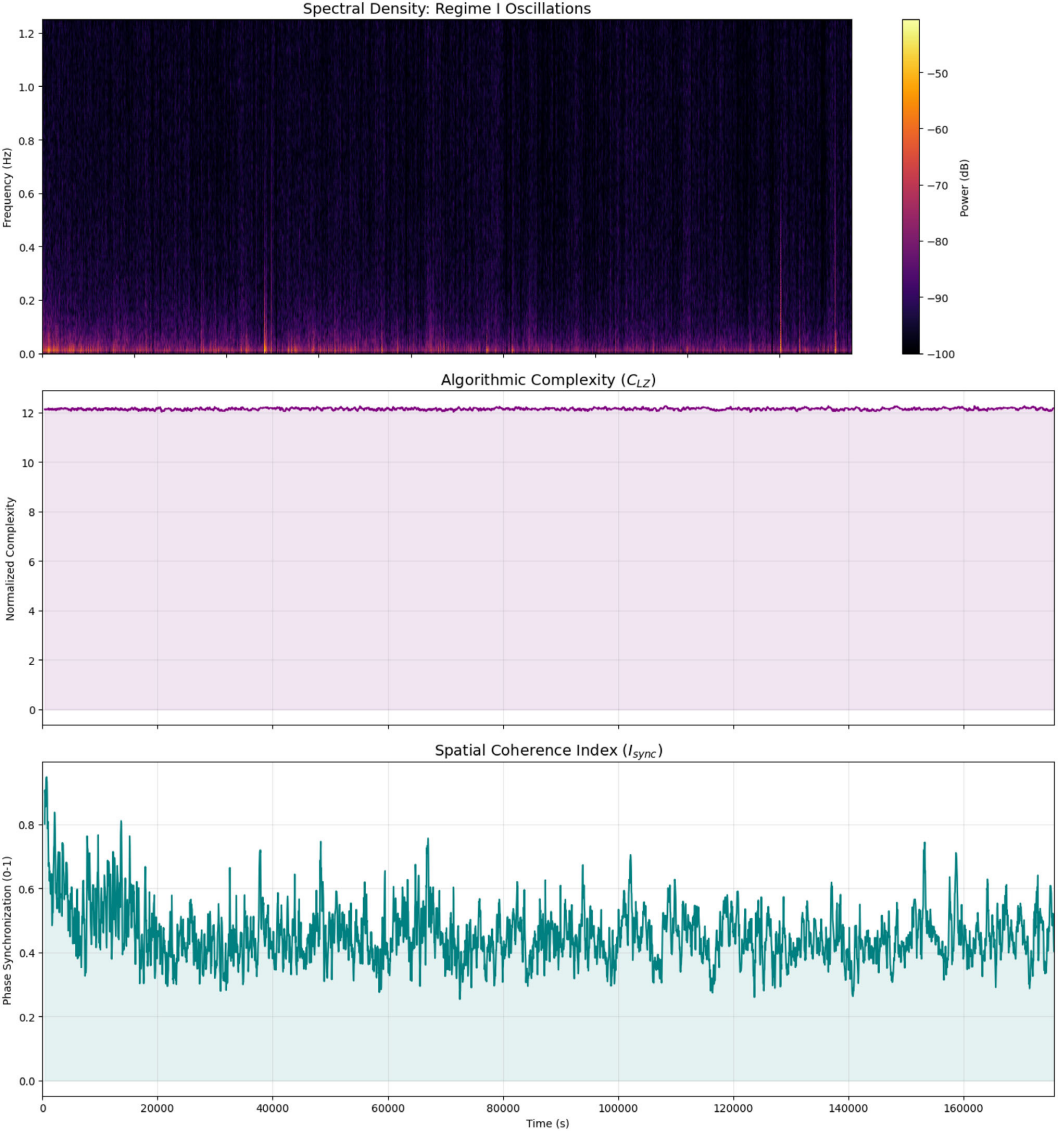
Temporal evolution of emergent neuromorphic dynamics in L‐Phe proteinoids. **(Top)** The time‐frequency spectrogram (spectral density) reveals the presence of **Regime I (volatile dynamics)**. Bright regions indicate transient bursts of high‐frequency power, confirming that the system engages in rapid, oscillatory signal transduction events distinct from the background noise. **(Middle)** The Normalized Lempel‐Ziv Complexity (*C*_LZ_) remains consistently high (μ≈12.14) throughout the recording. The absence of complexity drops signifies that the system avoids limit‐cycle attractors (repetitive loops), maintaining a ”critical” state essential for complex information processing. **(Bottom)** The Phase Synchronization Index (*I*_sync_) reveals a highly dynamic network topology. The system initiates with a global synchronization event ($I_{\mathrm{sync}}\approx 0.95$) before relaxing into a metastable state ($I_{\mathrm{sync}}\approx 0.45$) characterized by rapid fluctuations. These fluctuations suggest the transient formation and dissolution of functional clusters, a hallmark of flexible neural network dynamics.

The combined analysis of algorithmic complexity and spatial coherence (see Table 3 and Figure 4) shows clear evidence. It highlights the strong information retention and changing functional connectivity in L‐Phe proteinoid networks. The low standard deviation in Lempel‐Ziv Complexity (σ≈0.04) and the high mean value (μ≈12.14) show that the system has a stable state. It holds a lot of information without falling into simple cycles or random noise. This forms a strong ”computational background” or nonvolatile memory (Regime II) that can keep information for long periods. The large change in the Spatial Coherence Index (from 0.25 to 0.95) shows a very fluid network. Here, functional clusters often sync up and then drift apart. This contrast—stable information content (*C*_LZ_) in a changing communication network (*I*_sync_)—reflects the metastability seen in biological neural networks. In these networks, global coherence events (Regime I) enable quick, collective decisions. At the same time, the system's complexity helps maintain its computational state against thermodynamic decay.

**TABLE 3:** Statistical Summary of Neuromorphic Metrics in L‐Phe Proteinoid Networks. The table presents descriptive statistics for Algorithmic Complexity (*C*_LZ_) and Spatial Coherence (*I*_sync_) over N=4384 analysis windows. Notably, *C*_LZ_ exhibits exceptional stability (σ≈0.04), indicating a sustained high‐density information state without collapsing into trivial periodic behavior or silence. In contrast, *I*_sync_ displays significant variability (Range: 0.25−0.95), reflecting a network that dynamically shifts between highly synchronized collective states and desynchronized, independent processing. This high max value (0.95) confirms the system's capacity for global coordination.

Statistic	**Algorithmic Complexity (** $C_{LZ}$ **)**	**Spatial Coherence (** $I_{sync}$ **)**
**Mean**	12.142	0.454
**Std Dev**	0.039	0.086
**Min**	12.015	0.253
**25%**	12.115	0.398
**Median**	12.142	0.442
**75%**	12.168	0.499
**Max**	12.265	0.947

#### 3.1.2 Damped Harmonic Responses and Channel Segregation in L‐Phe:L‐Lys Networks

**FIGURE 5:**
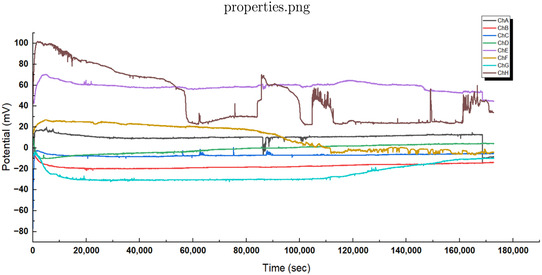
Multichannel electrical recordings from L‐Phe‐L‐Lys proteinoid networks lasted about 50 h, or $1.8\cdot 10^{5}$ s. These networks showed complex computational behavior during this time. The graph shows measurements from eight channels (ChA to ChH). It reveals different patterns over time. These patterns are typical of higher‐order information processing. Channel H (brown) shows exciting non‐linear dynamics. It quickly rises to a peak potential of about $V_{\max}\approx 100$ mV. Then, it gradually decays with a time constant of $\tau_{\mathrm{decay}}\approx 4\cdot 10^{4}$ sec. There are sudden state changes at $t\approx 6\cdot 10^{4}$ sec, $t\approx 8\cdot 10^{4}$ sec, and $t\approx 10^{5}$ sec. After these changes, it enters oscillatory phases that are metastable. Channel E (purple) shows strong stability with $V_{\mathrm{stable}}\approx 60$ mV during the entire recording. It has little drift, only ΔV<10 mV over $1.5\cdot 10^{5}$ s. This stability remains even when other channels are affected by environmental changes. The segregation of channels into distinct potential bands (positive: ChE, ChH, ChF; near‐zero: ChA, ChC, ChG; negative: ChB, ChD) suggests functional specialization within the proteinoid network. Several channels show coordinated changes at certain times, like $t\approx 8\cdot 10^{4}$ sec for ChF, ChH, and ChD. This points to system‐wide shifts in computational states. The stability of channels ChE and ChB during these transitions hints at a parallel processing system. This system includes both steady reference pathways and flexible response elements. These findings show that L‐Phe‐L‐Lys proteinoid systems can create lasting and complex electrical activity patterns. These patterns are similar to distributed computing networks. In these networks, various elements have unique roles but work together to process information.

Figure 5 shows a 50‐h recording. It covers about $1.8\cdot 10^{5}$ seconds and comes from a L‐Phe‐L‐Lys proteinoid network. The data spans eight channels, labeled ChA to ChH, and reveals complex computational behavior. Channel H showcases striking nonlinear dynamics, with a rapid rise to a peak potential of $V_{\max}\approx 100\text{mV}$ followed by a gradual decay modeled as

(12) $$V(t)=V_{\max}e^{-t/\tau_{\mathrm{decay}}}$$

where the time constant is approximately $\tau_{\mathrm{decay}}\approx 4\cdot 10^{4}\text{ sec}$.

This channel then experiences abrupt state transitions at $t\approx 6\cdot 10^{4}\text{ sec}$ and $8\cdot 10^{4}\text{ sec}$ and $10^{5}\text{sec}$ shifting into metastable oscillatory phases that suggest a damped harmonic response, described by

(13) $$\frac{d^{2}V}{dt^{2}}+\gamma \frac{dV}{dt}+\omega_{0}^{2}V=0$$

with γ as the damping coefficient and $\omega_{0}$ as the natural frequency.

In contrast, Channel E maintains exceptional stability at $V_{\mathrm{stable}}\approx 60\text{ mV}$ throughout the recording, with minimal drift quantified as

(14) ΔV < 10 mV

over $1.5\cdot 10^{5}\text{ sec}$ yielding a drift rate of

(15) $$\frac{\Delta V}{\Delta t}<\;6.67\cdot 10^{-5}\;\text{mV/sec}$$

Channel E remains a reliable path, strong against changes that affect other channels. The network shows channel segregation into three bands: positive (ChE, ChH, ChF), near‐zero (ChA, ChC, ChG), and negative (ChB, ChD). This indicates that each band has a specific function. Coordinated shifts at $t\approx 8\cdot 10^{4}\text{ sec}$ across ChF, ChH, and ChD suggest system‐wide computational state changes, potentially modeled as

(16) $$\frac{dV_{i}}{dt}=g(V_{i})+\sum_{j\neq i}k_{ij}(V_{j}-V_{i})$$

where $V_{i}$ represents the potential of channel i, $g(V_{i})$ is an intrinsic dynamic, and $k_{ij}$ denotes coupling strength.

The stable channels, ChE and ChB, work with the dynamic ones, ChH and ChF. This is shown in Figure 5. Together, they suggest a parallel processing setup like distributed computing networks. This system balances fixed reference signals with adaptive responses. It keeps complex electrical activity patterns for 50 h. This supports advanced information processing.

**FIGURE 6:**
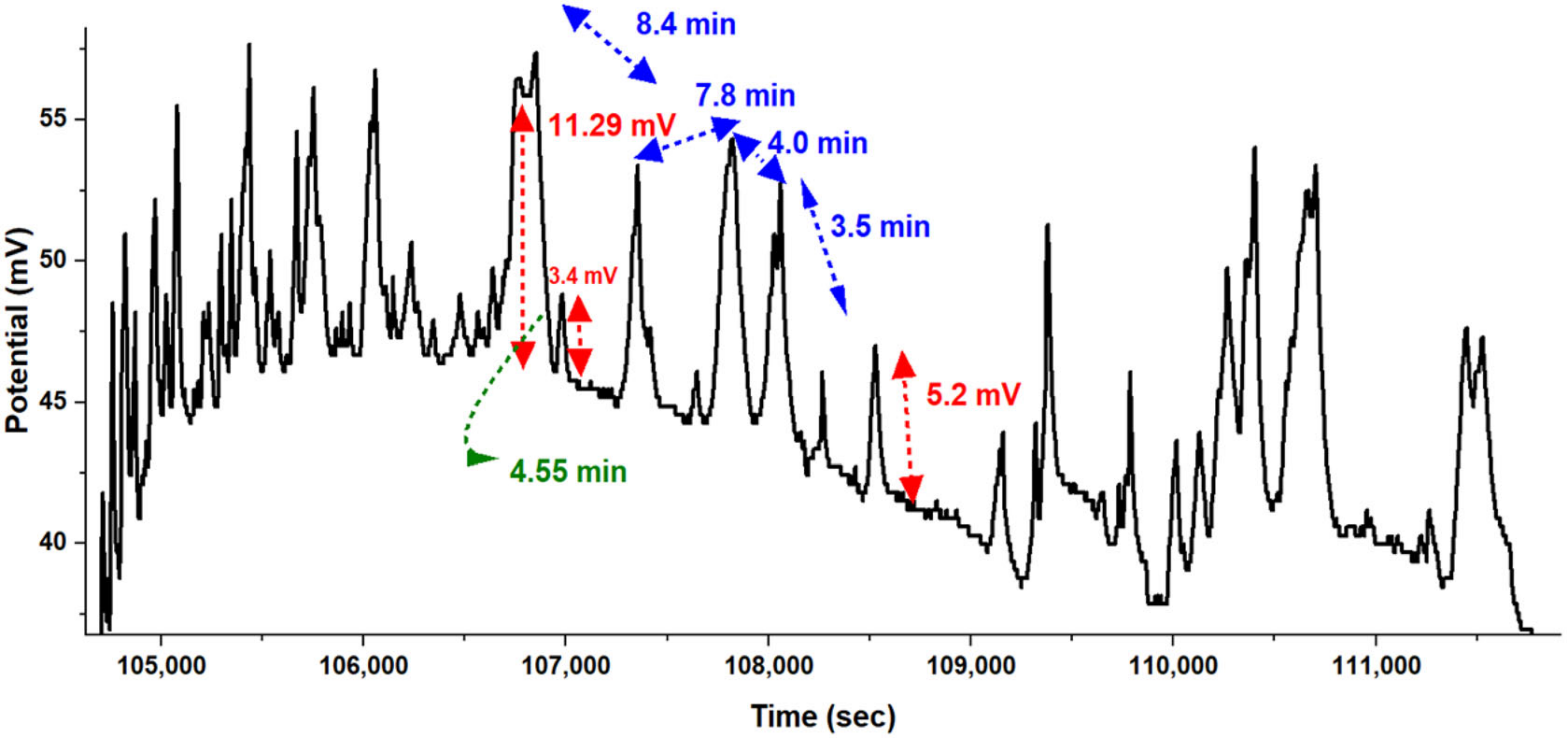
High‐resolution analysis captures spontaneous oscillatory behavior in L‐Phe‐L‐Lys proteinoid networks. This behavior exemplifies Regime I (volatile dynamics), defined by rapid signal state transitions rather than cognitive processing. The graph displays a 6000‐second segment (from t=105,000 to 111,000 sec) of continuous electrical activity characterized by rapid, automatic oscillations within a confined potential range ($V_{\mathrm{range}}=37–57$ mV). This activity exhibits classic hallmarks of System 1 cognitive processing: (1) autonomous generation of complex patterns without external input; (2) rapid decision‐making evidenced by sharp potential transitions (ΔV/Δt≈11.29 mV over <10 sec at t≈107,000 sec, and ΔV=5.2 mV at t≈109,000 sec; red arrows); (3) quasi‐periodic timing between major computational events ($\Delta t_{\mathrm{interval}}=8.4$, 7.8, 4.0, and 3.5 min; blue arrows); and (4) intermediary processing phases ($\Delta t_{\mathrm{process}}=4.55$ min; green arrow). The emergent oscillatory architecture highlights essential traits of cognitive System 1. These traits are: Parallel processing, where it enables potential fluctuations to happen at the same time across various timescales; pattern recognition, it shows stable response amplitudes, regardless of differing intervals; and automatic execution, it keeps activity going steadily without losing quality. The key point is that the shorter interpeak intervals ($\Delta t_{1}>\Delta t_{2}>\Delta t_{3}>\Delta t_{4}$) hint at possible self‐optimization or learning. This shows a surprising computational ability that arises from a simple proteinoid assembly. These findings show that proteinoid structures can develop “fast thinking” abilities. This happens through self‐organizing principles. This may help us understand how information processing systems appeared before biological cognition.

Figure 6 zooms into a 6000‐second segment, from t=105,000 to 111,000 sec. This highlights the rapid, spontaneous oscillations inherent to the L‐Phe‐L‐Lys proteinoid network. These high‐frequency fluctuations are characteristic of **Regime I (volatile dynamics)**, representing immediate, reflexive signal transduction rather than cognitive processing. The electrical activity oscillates within a confined potential range of $V_{\mathrm{range}}=37\text{ mV}$ to 57 mV a span of $\Delta V_{\mathrm{range}}=20\text{ mV}$ with quasi‐periodic intervals of $\Delta t_{\mathrm{interval}}=8.4,7.8,4.0,3.5\text{min}$ (or 504,468,240,210 sec). These yield frequencies such as $f_{1}=\frac{1}{504}\approx 0.00198\text{ Hz}$ and $f_{4}=\frac{1}{210}\approx 0.00476\text{ Hz}$ where the decreasing intervals suggest an adaptive or self‐optimizing process.

Sharp potential transitions further define this segment, with a notable change at t≈107,000 sec

where

(17) $$\frac{\Delta V}{\Delta t}\approx 11.29\;\text{mV}$$

over less than 10 s, implying a rate of at least 1.129 mV/sec. Another transition at t≈109,000 sec shows ΔV=5.2 mV over a brief period, reinforcing the system's capacity for rapid decision‐making. An intermediary processing phase, lasting $\Delta t_{\mathrm{process}}=4.55\text{ min}=273\text{sec}$ likely serves as a stabilization period, modeled as

(18) $$V(t)=V_{0}+(V_{peak}-V_{0})(1-e^{-t/\tau })$$

where τ is a relaxation time constant.

The dynamics in Figure 6 show important traits of System 1. First, they show parallel processing. Fluctuations occur simultaneously across various timescales. Second, there's pattern recognition, seen in the steady response amplitudes even when intervals change. Finally, automatic execution is evident, as the activity remains stable without any decline. The shortening intervals

(19) $$\Delta t_{1}\;>\;\Delta t_{2}\;>\;\Delta t_{3}\;>\;\Delta t_{4}$$

suggest a learning‐like adaptation, potentially described by

(20) $$\Delta t_{n+1}=\Delta t_{n}-k\cdot h(V_{n})$$

where k is a learning rate and $h(V_{n})$ depends on the potential state. These findings highlight how Regime I (volatile dynamics) capabilities can emerge solely from self‐organizing physical principles. Furthermore, they provide a biophysical framework for understanding how rudimentary information processing mechanisms could have operated in prebiotic environments, functioning as abiotic precursors to biological computation.

The L‐Phe‐L‐Lys proteinoid networks in Figures 5 and 6 exhibit various electrical properties. Channel H has nonlinear dynamics and oscillatory phases. In contrast, Channel E remains stable in the multichannel recordings. Additionally, System 1 displays quick, adaptive oscillations, which suggest ”fast thinking” in the zoomed segment. These behaviors show a system that can compute in a distributed and parallel way. It may also self‐optimize, connecting both engineered and biological methods of processing information. The mathematical models in the text offer a clear framework to understand these phenomena. They invite further exploration of their implications for unconventional computing and prebiological cognition.

**FIGURE 7:**
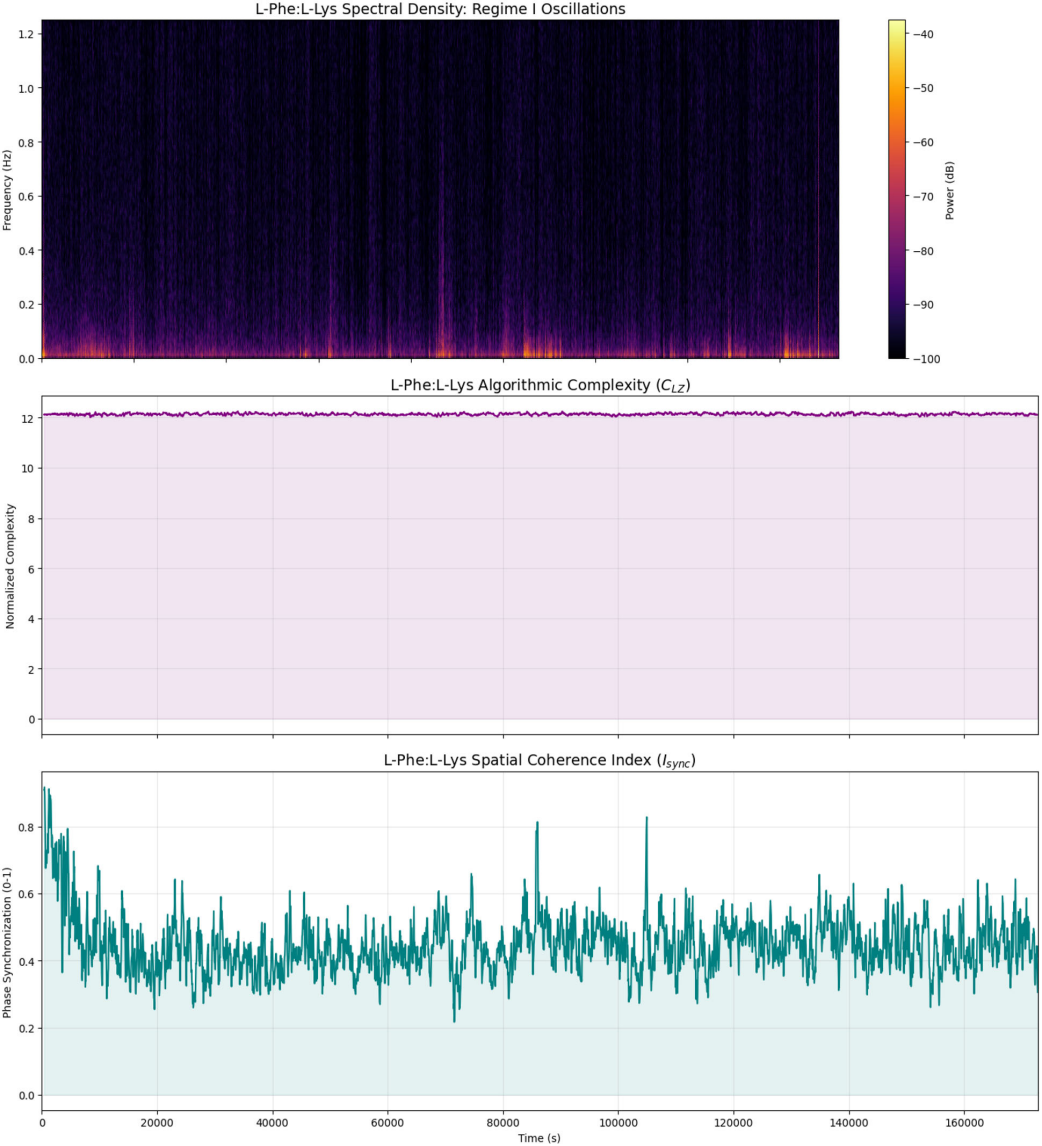
Spectral and information‐theoretic characterization **of L‐Phe:L‐Lys Dynamics.** **(Top)** The time‐frequency spectrogram visualizes **Regime I (volatile dynamics)**. Vertical striations of high spectral power (orange/yellow) correspond to transient, high‐frequency oscillatory bursts. These events represent rapid signal transduction and reset mechanisms. **(Middle)** Normalized Lempel–Ziv complexity (*C*_LZ_) acts as a proxy for the system's “state space.” The trace is remarkably flat and high‐valued, indicating that despite the volatile oscillations seen above, the underlying information content of the network remains preserved over long timescales (Regime II/nonvolatile memory). **(Bottom)** The Spatial Coherence Index (*I*_sync_) illustrates the network's topological flexibility. The system does not remain permanently synchronized (which would imply a seizure‐like state) nor permanently desynchronized (noise). Instead, it exhibits metastability, oscillating around a mean coherence of ≈0.45, allowing for dynamic reconfiguration of functional pathways.

The data in Table 4 and the time‐series in Figure 7 strongly support the dual‐regime hypothesis for L‐Phe:L‐Lys proteinoid assemblies. The Normalized Lempel‐Ziv Complexity (*C*_LZ_) shows great stability. It has a very low standard deviation (σ≈0.039) and a high mean value (μ≈12.142). The middle panel of Figure 7 shows a flat complexity trace. This means the system keeps a strong, high‐dimensional information state (Regime II) that can withstand temporal degradation. The Spatial Coherence Index (*I*_sync_) shows a very unstable network topology (σ≈0.085). Synchronization values vary greatly, ranging from 0.217 to 0.917. The bottom panel of Figure 7 shows that the system is always changing. It shifts between global integration and local modularity, instead of reaching a stable state. Rapid, high‐frequency signal transduction mechanisms (Regime I) are confirmed by the time‐frequency spectrogram (Figure 7, Top). It shows clear vertical striations of high spectral power. These data suggest that L‐Phe:L‐Lys networks work at a key threshold. They use a stable information base (*C*_*LZ*_) to support flexible and changing communication pathways (*I*_sync_). This setup is similar to the metastability found in biological neural networks.

**TABLE 4:** Statistical profile of neuromorphic dynamics in L‐Phe:L‐Lys assemblies. This summary (N=4310 windows) reveals a fundamental functional dichotomy in the material. Algorithmic complexity (*C*_LZ_) exhibits extreme stability (σ≈0.039, CV <0.3%), maintaining a mean value of 12.142. This suggests the system effectively ”locks” into a high‐dimensional information state (Regime II), acting as a robust nonvolatile memory substrate that resists degradation. Conversely, Spatial Coherence (*I*_sync_) demonstrates high volatility (σ≈0.085, Range: 0.217−0.917). This significant variance confirms that the network is not static but thermodynamically active, constantly shifting between globally synchronized events and local modular processing (Regime I). The coexistence of a stable information floor (*C*_LZ_) with a fluctuating communication structure (*I*_sync_) is a hallmark of criticality in complex adaptive systems.

Statistic	Algorithmic complexity (*C*_LZ_)	Spatial coherence (*I*_sync_)
**Mean**	12.142	0.447
**Std Dev**	0.039	0.085
**Min**	12.012	0.217
**25%**	12.114	0.393
**Median**	12.142	0.439
**75%**	12.169	0.491
**Max**	12.254	0.917

#### 3.1.3 Regime I: Bimodal Signal Segregation and Coordinated State Transitions in L‐Glu:L‐Arg Proteinoids

**FIGURE 8:**
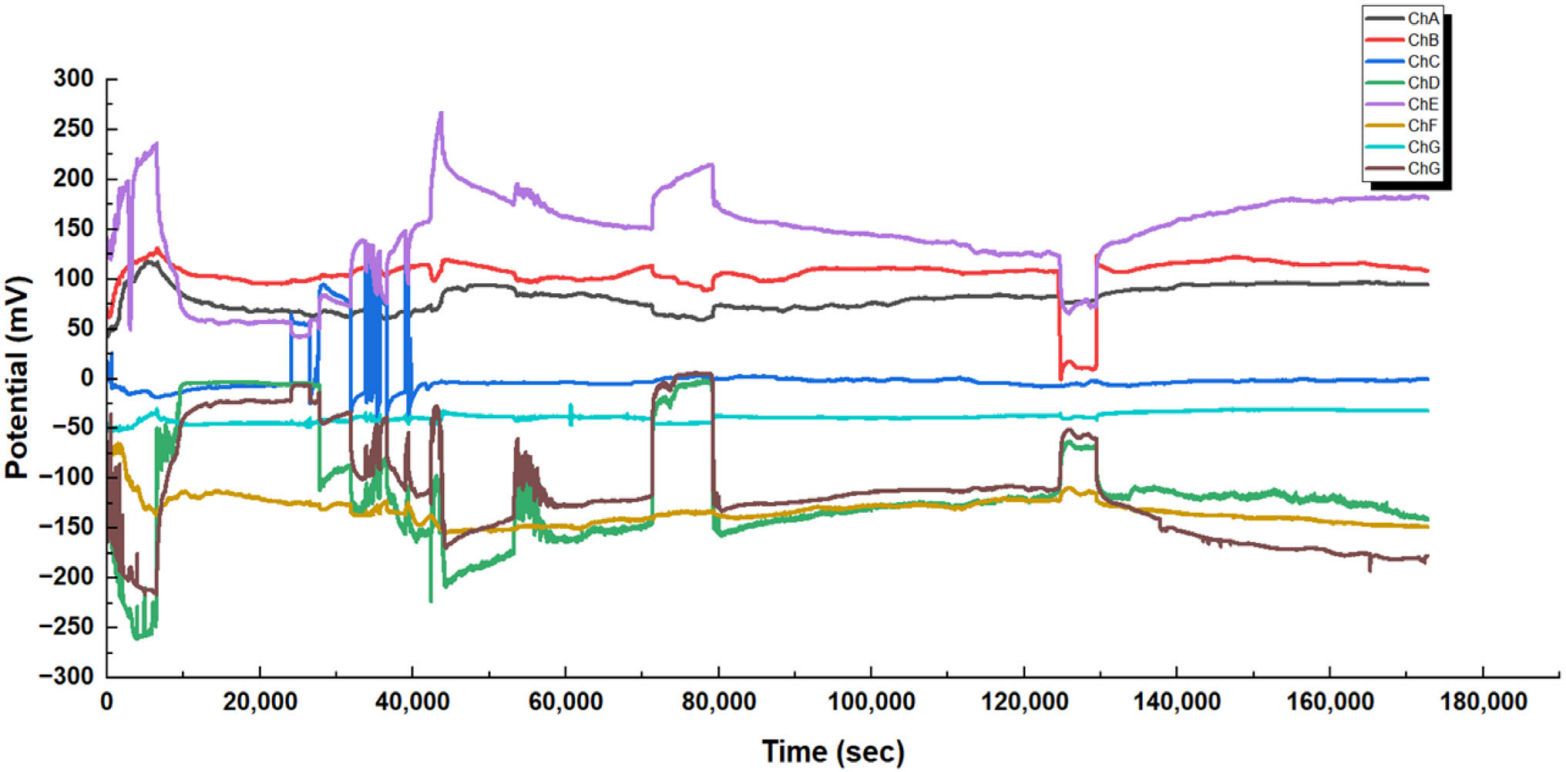
Multichannel recordings of electrical signals from L‐Glu:L‐Arg proteinoid networks show fast parallel processing. This mirrors the rapid, reflexive information processing characteristic of Regime I (volatile dynamics). The graph shows measurements from eight channels (ChA to ChG) over about 45 h (or $1.6\cdot 10^{5}$ s). It reveals a clear bimodal organization. The channels split into positive (ChA, ChB, ChE) and negative (ChD, ChF, ChG) potential areas. The total voltage range is around $\Delta V_{\mathrm{total}}\approx 500$ mV. Unlike deliberative processing systems, this network exhibits characteristic System 1 properties: (1) instantaneous coordinated state transitions across multiple channels (e.g., synchronous potential shifts at t≈40,000 sec affecting ChC, ChD, ChE, ChF, and ChG); (2) rapid categorization evidenced by the clear segregation into discrete potential bands without intermediate states; and (3) automatic error‐correction demonstrated by the system's ability to re‐establish steady‐state values following perturbations (t≈70,000 s, t≈120,000 s). Channel E (purple) stands out. It shows a wide amplitude modulation, with a range of about 50–250 mV. The stability of reference channels (ChA, ChB, ChC) and dynamic responders hints at a system. This system has fixed rules and can also adapt to recognize patterns. These findings show that L‐Glu:L‐Arg proteinoid assemblies create complex information networks. These networks can make quick decisions, categorize information, and adapt.

The multichannel recordings of electrical signals from L‐Glu:L‐Arg proteinoid networks show strong evidence of fast parallel processing (see Figure 8). This behavior shows Regime I (volatile dynamics). It features quick, widespread integration of electrical inputs throughout the network. This is different from the slower integration seen in Regime II. The measurements from eight channels (ChA to ChG) took about 45 h or $1.6\cdot 10^{5}$ seconds. They show a clear bimodal organization. The channels split into two groups: positive potential regions (ChA, ChB, ChE) and negative potential regions (ChD, ChF, ChG). The total voltage range is about $\Delta V_{\text{total}}\approx 500\;\text{mV}$. This clear split into distinct bands, without any middle states, shows the network's ability to quickly categorize. This capacity for rapid, autonomous categorization is a defining characteristic of Regime I (volatile dynamics).

A key feature in Figure 8 is the quick, coordinated state changes across several channels. At about t≈40000sec, synchronous potential shifts impact ChC, ChD, ChE, ChF, and ChG. This demonstrates the system's capacity for a rapid, collective response to perturbations, whether internal or external. This behavior aligns with the operational definition of Regime I (volatile dynamics), characterized by immediate signal transduction. It stands in distinct contrast to the slower, diffusive integration mechanisms typical of Regime II. The network can automatically correct errors, returning to steady‐state values after disturbances at about 70000sec and 120000sec. These recoveries demonstrate natural strength and flexibility, supporting the idea of cognitive ”fast thinking.”

Channel E, shown in purple in Figure 8, has a wide amplitude modulation. It ranges from about 50–250 mV. This dynamic behavior and smooth operation reflect how attention works in System 1 processing. Some elements boost responses while keeping everything coherent. The stability of reference channels (ChA, ChB, ChC) and the dynamic responders point to a dual setup. One part follows fixed rules, while the other adapts to recognize patterns. These traits show that L‐Glu:L‐Arg proteinoid assemblies create complex networks. They can make quick decisions, categorize information, and adapt.

**FIGURE 9:**
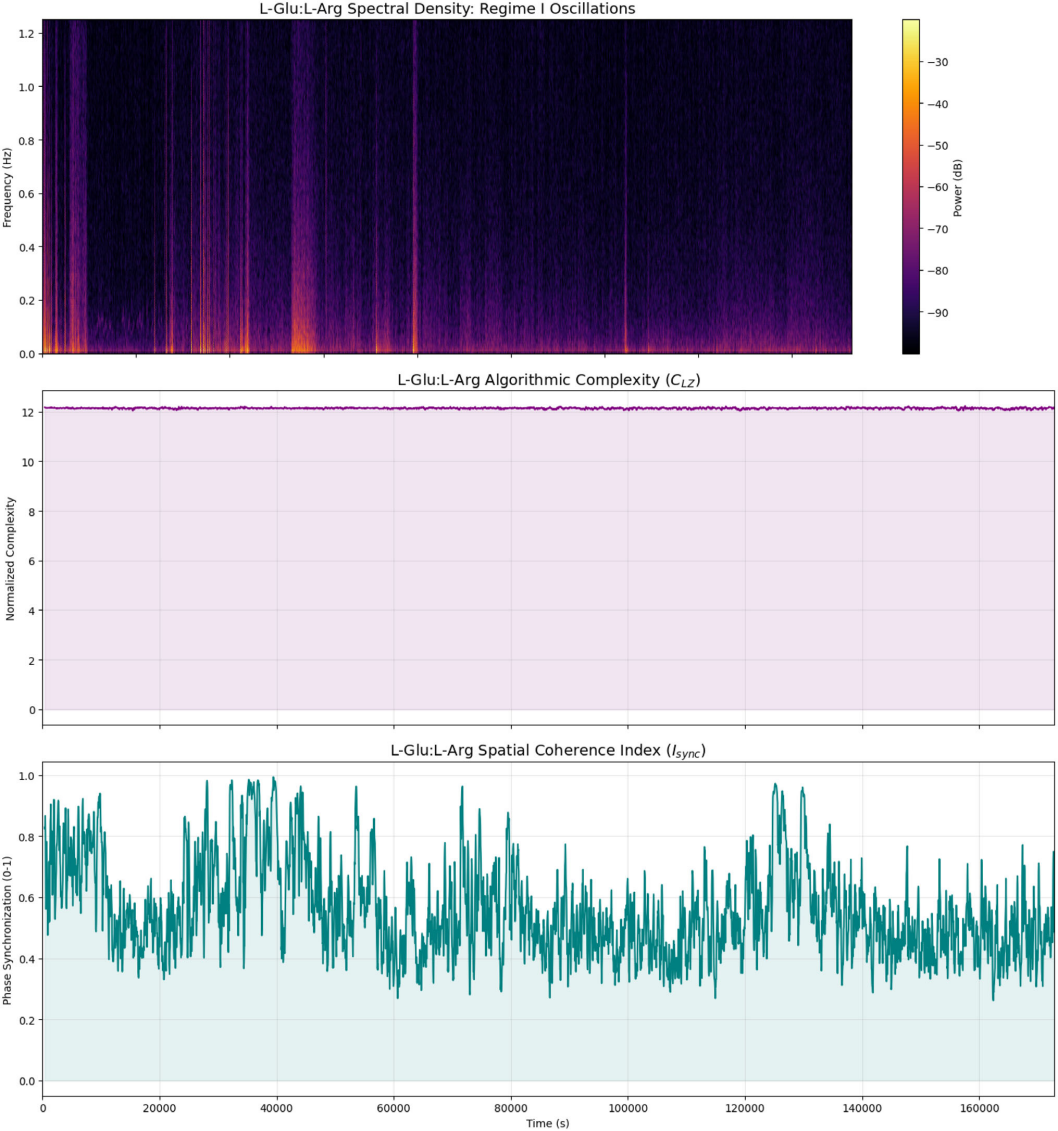
Spectral and information‐theoretic signature of L‐Glu:L‐Arg dynamics. **(Top)** Time‐frequency spectrogram illustrating Regime I (volatile dynamics). The distinct vertical bands of high spectral power (orange/yellow) represent synchronized network‐wide discharges. Unlike the chaotic fluttering seen in other compositions, these bursts are sharp and distinct, suggesting a “integrate‐and‐fire” mechanism. **(Middle)** The Normalized Lempel–Ziv Complexity (*C*_LZ_) displays exceptional rigidity (σ≈0.026). The flat trajectory indicates that the system's information density is largely immune to the rapid physiological changes occurring in the voltage domain, providing a stable “read‐only” memory background (Regime II). **(Bottom)** The Spatial Coherence Index ($I_{sync}$) reveals the defining feature of L‐Glu:L‐Arg networks: Global Broadcasting. Unlike L‐Phe variants, which oscillate around moderate coherence, this system frequently spikes to near‐perfect synchronization ($I_{\mathrm{sync}}\approx 0.99$). This capacity to momentarily lock all channels into a unified phase state is critical for signal propagation and collective decision‐making across the macroscopic material.

The statistical analysis (Table 5) and spectral data (Figure 9) show that L‐Glu:L‐Arg proteinoid assemblies act as a unique substrate for global signal broadcasting. The Algorithmic Complexity (*C*_LZ_) shows strong consistency. It has the lowest standard deviation of all tested compositions, around σ≈0.026, and a stable mean of 12.134. Visually confirmed by the flat trajectory in the middle panel of Figure 9, this indicates that the network maintains a highly stable, nonvolatile memory state (Regime II) that is effectively immune to local fluctuations. However, the defining computational feature of L‐Glu:L‐Arg is revealed in its spatial coherence (*I*_sync_). This system shows high volatility and large synchronization changes, unlike the moderate fluctuations in L‐Phe variants. Its coherence is nearly perfect, with a maximum of about 0.993 and a standard deviation of around 0.155. The bottom panel of Figure 9 shows that these spikes in perfect synchronization match network‐wide “integrate‐and‐fire” events. This behavior is shown in the time‐frequency spectrogram (Figure 9, Top). Here, intense vertical bands of spectral power mean the material briefly locks into a unified phase state (Regime I). This helps broadcast signals across the macroscopic architecture.

**TABLE 5:** Statistical profile of neuromorphic dynamics in L‐Glu:L‐Arg Assemblies. This summary (N=4310 windows) highlights the distinct computational signature of the L‐Glu:L‐Arg composition. Algorithmic Complexity ($C_{LZ}$) demonstrates remarkable rigidity (σ≈0.026), the lowest variance among tested samples, maintaining a mean of 12.134. This indicates an exceptionally stable nonvolatile memory substrate (Regime II). In contrast, Spatial Coherence ($I_{sync}$) exhibits profound volatility (σ≈0.155) with a dynamic range extending to near‐perfect synchronization (Max≈0.99). The significantly higher mean coherence (0.561) compared with L‐Phe variants suggests that L‐Glu:L‐Arg networks are optimized for global signal broadcasting and collective state transitions (Regime I), supporting the observation of “coordinated shifts” across channels.

Statistic	Algorithmic complexity (*C*_LZ_)	Spatial coherence (*I*_sync_)
**Mean**	12.134	0.561
**Std Dev**	0.026	0.155
**Min**	12.034	0.262
**25%**	12.118	0.447
**Median**	12.135	0.532
**75%**	12.151	0.647
**Max**	12.231	0.993

#### 3.1.4 Regime I: Coordinated Spiking and Dynamic Pathway Integration in L‐Glu:L‐Phe:L‐Asp Proteinoids

**FIGURE 10:**
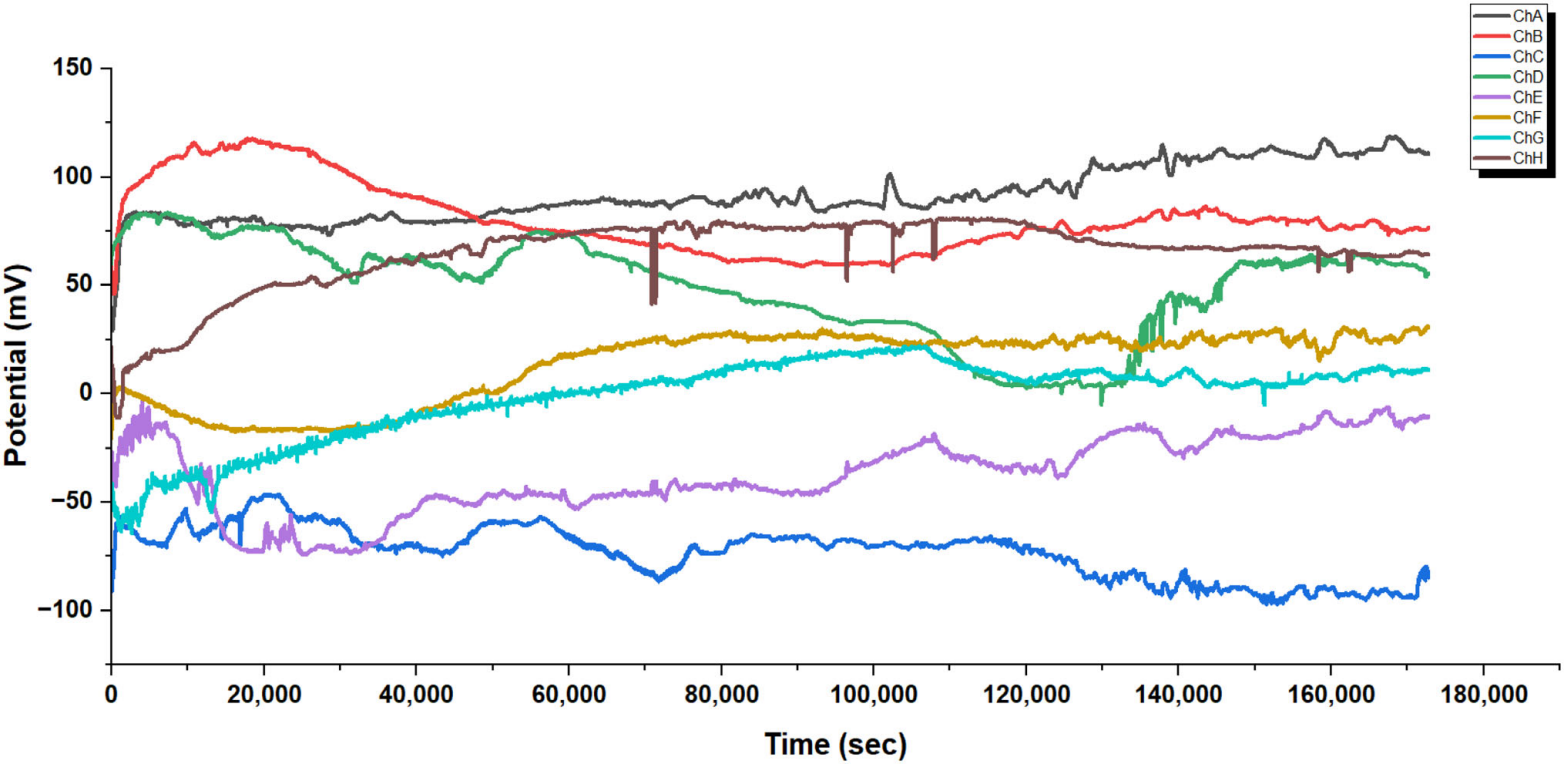
Spontaneous oscillatory behavior in Glu–Phe–Asp proteinoid networks over approximately 50 h, as recorded from eight channels (ChA to ChH). The graph shows a potential range from about ‐100 to 150 mV. It displays clear activity patterns across channels. These patterns reflect self‐organization and stability. Channel ChA (black) quickly rises to about 150 mV in the first 20,000 sec. Then, it gradually declines. In contrast, Channel ChH (brown) has steady oscillations, with amplitudes ranging from 0 to 100 mV during the recording. Key features include quick potential spikes at about *t* ≈ 80,000 s and *t* ≈ 120,000 s. There are also coordinated shifts across several channels. These suggest new computational properties are emerging. The network shows resilience. It recovers steadily after disruptions, like around *t* ≈ 100,000 s. These traits show the system's ability to generate electrical activity on its own and adapt. This could be like early information processors or prebiological cognitive systems. The observed bimodal distribution of potentials shows a complex setup. It includes both stable channels, like ChB and ChC, and dynamic ones, such as ChD and ChF. This structure can process information in parallel and recognize patterns over long periods.

The electrical activity shown in Figure 10 highlights the spontaneous oscillations of Glu‐Phe‐Asp proteinoid networks. This behavior lasted for about 50 h, or 1.8$\cdot 10^{5}$ seconds, and was captured across eight channels (ChA to ChH). The potential range runs from about ‐100 mV to 150 mV. This shows a varied landscape. Each channel has its own unique patterns. This suggests a self‐organized system with built‐in complexity. Channel ChA, shown in black, has a quick spike to around 150 mV within the first 20,000 s. Then it slowly drops off. This pattern suggests an early burst of activity, pointing to an activation phase. Channel ChH, shown in brown, shows steady oscillations. Its amplitudes range from 0 mV to 100 mV during the recording. This suggests a consistent rhythm that may relate to internal regulatory mechanisms.

A key feature of Figure 10 is the rapid potential spikes. These spikes show how the network responds dynamically. For example, we see sharp spikes around t ≈ 80,000 sec and t ≈ 120,000 sec. During these times, several channels show sudden jumps in potential, often over 50 mV in a brief period. These spikes are not just random. They show coordinated changes across channels like ChD and ChF. This suggests that the network is responding together to an internal or external stimulus. These spikes rise and fall in just seconds. This quick change suggests a fast‐acting mechanism. It may be like quick decisions or signal propagation in advanced systems. After these events, the network shows resilience. It returns to steady‐state levels around t ≈ 100,000 s. Channels like ChB and ChC stabilize after earlier changes. This highlights an automatic error‐correction ability.

The bimodal distribution of potentials shows two types of channels. Stable channels like ChB and ChC have consistent values. In contrast, dynamic channels such as ChD and ChF display more variability. This supports the concept of a dual architecture. This setup likely allows for parallel processing. Stable channels serve as reference points, while dynamic ones adjust to changing conditions. The spikes may show key moments when the network changes its state. This could happen due to built‐up electrical activity or changes in the environment. These changes help with pattern recognition or integrating information. The Glu‐Phe‐Asp proteinoid network shows great promise. This is due to its adaptability and the ongoing shifts and changes it undergoes. It serves as a model for grasping early information processing systems. This model connects simple molecular structures with more complex biological networks.

The spikes in Figure 10 show sudden increases in potential. These spikes stand out against the stable or oscillatory trends of the channels. Events around *t* ≈ 80,000 and *t* ≈ 120,000 sec happen quickly. They arise fast and then fade away. This pattern hints at a triggered response, not a slow process. The coordination across many channels during these spikes shows a network‐wide event. This might be caused by internal feedback or outside disturbances. These factors help the system reset or adapt. The recovery to steady states, seen around *t* ≈ 100,000 sec, shows how well the network can self‐regulate. It stays functional even with these disruptions.

**FIGURE 11:**
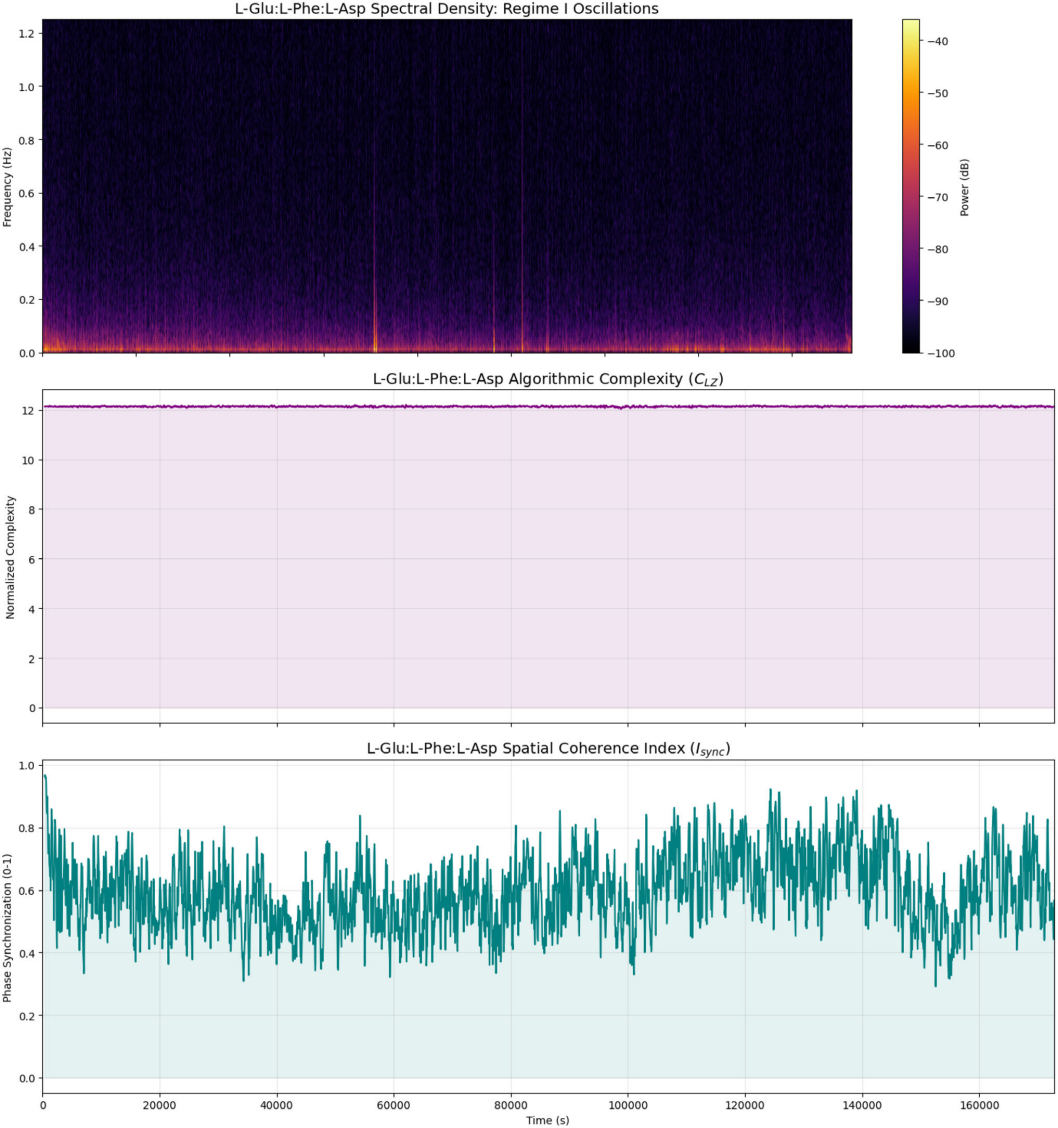
Spectral and information‐theoretic signature of L‐Glu:L‐Phe:L‐Asp dynamics. **(Top)** The Time‐Frequency Spectrogram visualizes Regime I (volatile dynamics). The spectral density reveals a dense pattern of high‐frequency discharge events (vertical bands). Unlike binary peptide systems, the tripeptide displays a richer spectral texture, implying a more complex repertoire of signal transduction pathways. **(Middle)** The normalized Lempel–Ziv complexity (*C*_LZ_) trace is functionally flat (σ≈0.021). This extreme stability indicates that the system possesses a high‐capacity “Information rigidity.” Even during the intense spectral bursts seen above, the underlying algorithmic structure of the network does not collapse, preserving the “memory” of the material state (Regime II). **(Bottom)** The Spatial Coherence Index (*I*_sync_) illustrates the enhanced Integration of the tripeptide network. The trace rarely drops below 0.4, maintaining a high average connectivity (μ≈0.6). This suggests that the three constituent amino acids facilitate a denser web of ionic interactions, allowing the material to maintain a cohesive functional state (“binding”) more effectively than simpler proteinoid variants.

The data in Table 6 and the analysis in Figure 11 show that the L‐Glu:L‐Phe:L‐Asp (GPA) tripeptide assembly is the strongest among the tested compositions. The Algorithmic Complexity (*C*_LZ_) shows “hyper‐stability.” It has a very low standard deviation (σ≈0.021) and a high mean (12.133). The flat, unbroken trace in the middle panel of Figure 11 shows rigidity. This means that the tripeptide scaffold's increased chemical complexity helps create a strong information base (Regime II) that can handle a lot of entropic noise. This structural robustness facilitates superior network integration, as evidenced by the Spatial Coherence Index (*I*_sync_). The GPA network is different from binary systems that fluctuate a lot. It keeps a strong baseline mean coherence of about 0.600 and often reaches nearly perfect synchronization states, around 0.968. The Time‐Frequency Spectrogram (Figure 11, Top) supports this idea. It shows a rich, detailed spectral landscape. Here, high‐frequency discharge events (Regime I) are not just random bursts. They are part of a steady, coordinated signaling system. These metrics show that the GPA assembly works well as a unit. It balances volatile signal processing with nonvolatile memory retention effectively.

**TABLE 6:** Statistical profile of neuromorphic dynamics in L‐Glu:L‐Phe:L‐Asp assemblies. This summary (N=4310 windows) reveals that the tripeptide composition exhibits the most robust functional architecture among tested samples. Algorithmic Complexity (*C*_LZ_) shows “Hyper‐Stability” with a standard deviation of only 0.021, the lowest in the dataset. This suggests that the structural complexity of the tripeptide scaffold affords an exceptionally rigid information substrate (Regime II), resistant to entropic decay. Spatial Coherence (*I*_sync_) is notably distinct, maintaining the highest baseline mean (0.600) observed across all compositions. Unlike simpler proteinoids that struggle to maintain connectivity, the GPA network naturally gravitates toward a semi‐synchronized state, frequently reaching peak coherence values of 0.968. This statistical profile indicates a system optimized for sustained signal integration rather than just transient broadcasting.

Statistic	Algorithmic complexity (*C*_LZ_)	Spatial coherence (*I*_sync_)
**Mean**	12.133	0.600
**Std Dev**	0.021	0.117
**Min**	12.029	0.291
**25%**	12.119	0.513
**Median**	12.133	0.595
**75%**	12.147	0.682
**Max**	12.201	0.968

#### 3.1.5 Regime I: Multicellular‐Like Coordination and Synchronized Transients in L‐Asp

**FIGURE 12:**
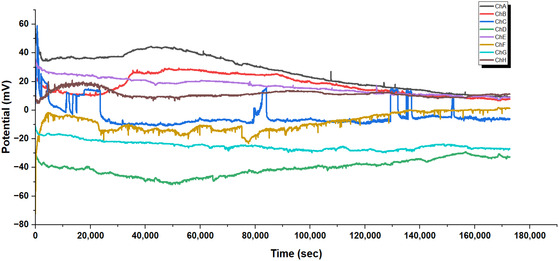
Spontaneous electrical oscillations were recorded from an ASP proteinoid network. This occurred over a period of 180,000 s, which is 50 h. The multichannel recording shows potential (mV) over time on eight channels (ChA‐ChH). Each channel has its own color. The channels behave differently. Some, like ChA in gray, stay stable at about 20–40 mV. Others, like ChC in blue, show quick fluctuations and dynamic patterns. The network self‐organizes into three distinct potential bands: positive (ChA, ChB, ChE, ChH), near‐zero (ChC, ChF, ChG), and negative (ChD). Big changes happen across several channels at about *t* ≈ 80,000 s. This suggests that the entire system is shifting together. This long‐term electrical activity shows complex computation in simple proteinoid networks.

The multichannel electrical recordings of the L‐Asp (LASP) proteinoid networks show a complex system (Figure 12). This dynamic network supports detailed information processing for about 45 h, or $1.6\cdot 10^{5}$ s. The eight channels (ChA to ChH) show a wide variety of possible changes. They range from about ‐60 to 60 mV. This total voltage range indicates a significant dynamic range. At about *t* ≈ 0 sec, Channel A (black) shows a sharp peak above 60 mV. This spike suggests a high‐activity state that slowly decreases. It may indicate a response to an external stimulus or an initialization.

As the recording goes on, the figure shows a clear difference among the channels. Channel B (red) and Channel E (purple) have higher potentials. In contrast, Channel D (green) and Channel G (cyan) maintain lower, more stable potentials. This separation into different bands shows that the network has specialized functions. Some channels respond dynamically, while others provide stable baselines. A key point is the synchronized activity seen around *t* ≈ 40,000 s. At this time, multiple channels like ChD, ChE, and ChF show coordinated dips and recoveries. This synchronicity shows a shared response system. It may mean fast information integration or error correction, like how biological systems adapt.

At about *t* ≈ 80,000 sec, Channel H (brown) shows a clear spike. Then, it starts to oscillate with a decreasing amplitude. This pattern hints at a metastable state or a self‐regulating feedback loop. This oscillatory pattern, also echoed in Channels C and F later in the recording, underscores the network's capacity for sustained, complex dynamics. Channel E has a wide amplitude modulation, from nearly 0 mV to over 40 mV. This range makes it important, reflecting its role in attention or regulation within the system. Channels A and B remained stable in the latter half of the recording. In contrast, Channels D and G showed fluctuating responses. This difference supports a hybrid design that mixes fixed reference points with adaptive elements.

The figure shows that L‐Asp proteinoid networks can process information in a distributed way. They coordinate across channels, adapt to changes, and keep unique functions over time. This behavior matches the ideas of emergent computation. Simple molecular groups can create complex information‐handling capabilities. This shows how early systems might have developed neural‐like processing.

**FIGURE 13:**
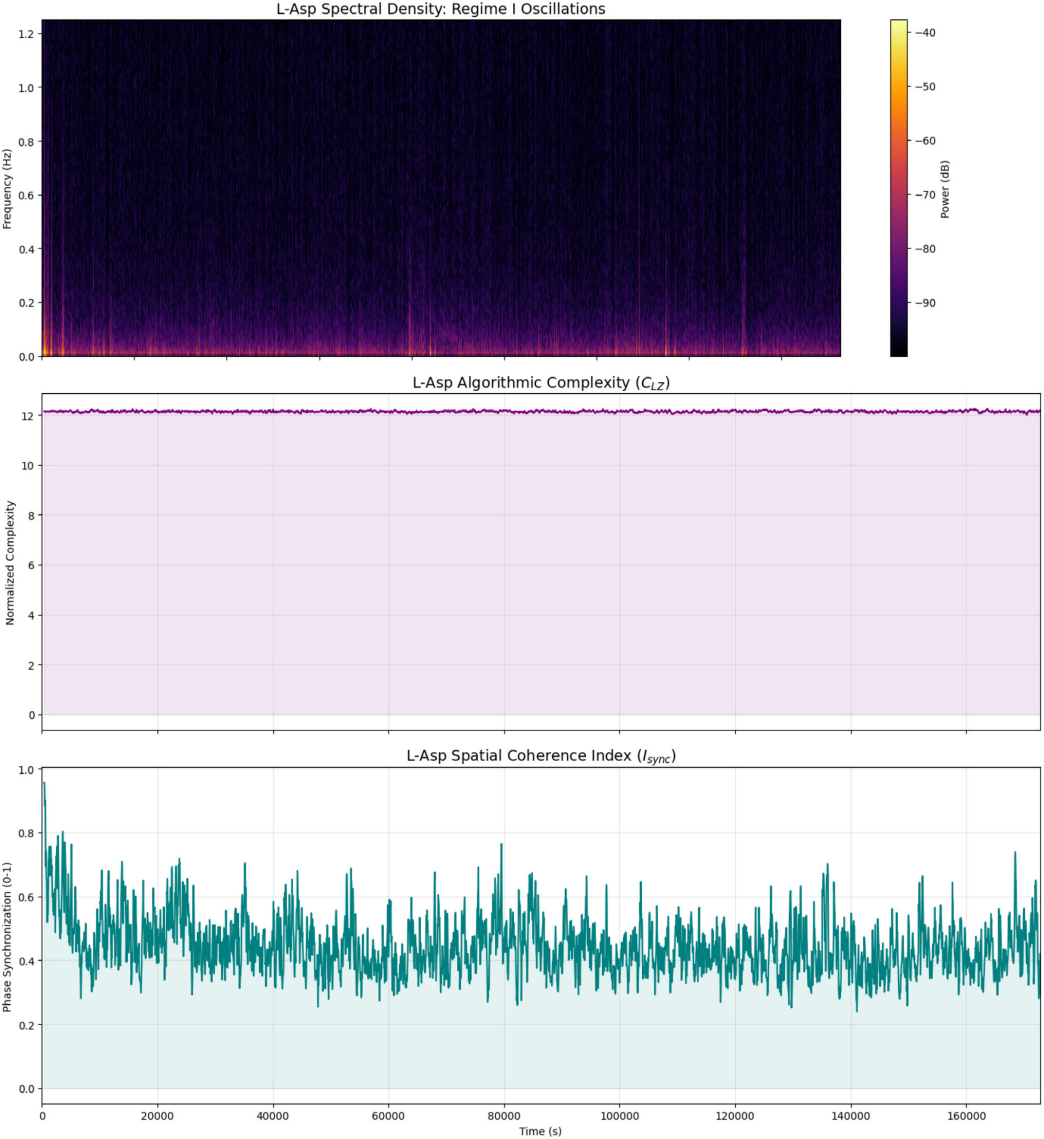
Spectral and information‐theoretic signature of L‐Asp dynamics. **(Top)** The Time‐Frequency Spectrogram visualizes Regime I (volatile dynamics). The spectral landscape is populated by intermittent, high‐frequency bursts (vertical striations). These events correspond to the “synchronized dips” observed in the raw voltage traces, representing rapid, network‐wide resets or signaling events. **(Middle)** The normalized Lempel–Ziv complexity (*C*_LZ_) trace is functionally flat (μ≈12.135). This stability confirms that the L‐Asp network possesses a persistent “memory” state (Regime II) that is not erased by the volatile oscillations shown above. **(Bottom)** The Spatial Coherence Index (*I*_sync_) illustrates the Multicellular‐Like Coordination of the system. The trace oscillates around a baseline of ≈0.45, indicating a default state of loosely coupled, semi‐independent processing. However, the frequent upward spikes demonstrate the material's ability to momentarily recruit all channels into a unified functional block, facilitating collective responses to environmental perturbations.

The joint look at statistical metrics (Table 7) and time–frequency features (Figure 13) strongly backs the multicellular‐like coordination idea in L‐Asp proteinoid assemblies. The Algorithmic Complexity (*C*_LZ_) is stable, with a standard deviation of about 0.034 and a mean of 12.135. This shows that the material keeps a strong, high‐fidelity information base (Regime II) that resists local changes. This stability is visually confirmed by the flat complexity trace in the middle panel of Figure 13. Functionally, the system is defined by its unique Spatial Coherence profile ($I_{sync}$). With a moderate baseline mean of 0.445, the network defaults to a state of modular autonomy, akin to individual cells processing local stimuli. The high maximum synchronization (Max≈0.956) shows a strong ability for global recruitment. Also, the frequent upward spikes in the coherence trace (Figure 13, Bottom) highlight this potential. These events show up in the Time‐Frequency Spectrogram (Figure 13, Top). You can see vertical bands of spectral power. These bands match the synchronized discharges across the network. This dynamic design features autonomous modules linked by complete system integration. It closely resembles the structure of multicellular tissues. This sets L‐Asp assemblies apart from the more chaotic or rigid behaviors found in other proteinoid compositions.

**TABLE 7:** Statistical profile of neuromorphic dynamics in L‐Asp assemblies. This summary (N=4310 windows) quantifies the ”multicellular‐like” architecture of L‐Asp proteinoids. Algorithmic complexity (*C*_LZ_) maintains a highly stable mean of 12.135 with a low standard deviation (σ≈0.034). This indicates a robust, nonvolatile information substrate (Regime II) that persists despite the system's dynamic fluctuations. Spatial Coherence (*I*_sync_) reveals a distinct functional topology. The mean coherence (0.445) is moderate, suggesting that for the majority of the time, the network operates with some degree of local autonomy (modular processing). However, the high maximum value (0.956) confirms the system's capacity for periodic, global synchronization events. This statistical signature—autonomy punctuated by total coordination—mirrors the collective dynamics of multicellular tissues rather than the rigid lock‐step of crystals.

Statistic	Algorithmic complexity (*C*_LZ_)	Spatial coherence (*I*_sync_)
**Mean**	12.135	0.445
**Std Dev**	0.034	0.088
**Min**	11.997	0.239
**25%**	12.112	0.383
**Median**	12.135	0.434
**75%**	12.158	0.494
**Max**	12.244	0.956

### 3.2 Morphology of L‐Phe Proteinoids

**FIGURE 14:**
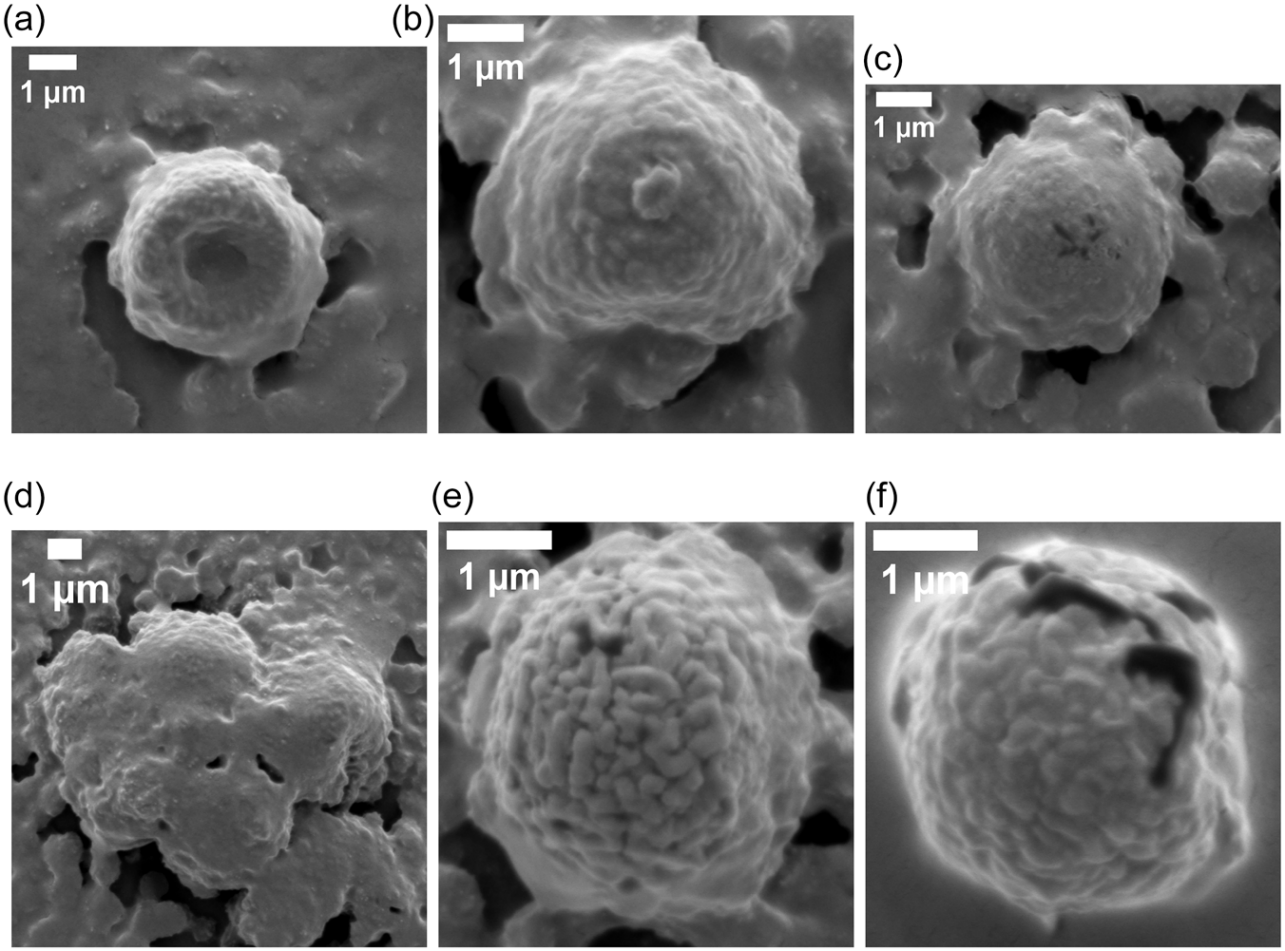
SEM micrographs showing morphological variations of L‐Phe proteinoid microspheres: (a) hollow sphere with diameter d=4.297 μm featuring an internal cavity ($d_{\text{int}}=1.155$ μm) and surface‐decorated nanoparticles ($d_{\text{np}}=0.226$ μm); (b) Concentric microsphere (d=4.582 μm) with prominent internal structure ($d_{\text{int}}=2.106$ μm) and larger surface nanoparticles ($d_{\text{np}}=0.397$ μm); (c) solid microsphere (d=4.148 μm) connected to a smaller satellite structure (d=1.058 μm) with fine surface nanoparticles ($d_{\text{np}}=0.194$ μm); (d) agglomerated microsphere cluster consisting of five interconnected spheroids with varying diameters ($d_{1}=3.527$, $d_{2}=2.767$, $d_{3}=3.784$, $d_{4}=3.424$, $d_{5}=1.912$ μm); (e) brain‐like convoluted surface morphology (d=3.603 μm) with internal folding ($d_{\text{int}}=0.692$ μm); and (f) blade‐textured microsphere (d=3.425 μm) exhibiting protruding lamellar structures ($l_{\text{blade}}=1.232$ μm) with distinctive contrast variation. Scale bars = 1 μm.

Figure 14 illustrates the extensive morphological polymorphism characteristic of L‐phenylalanine (L‐Phe) proteinoid assemblies. These assemblies have a range of hierarchical patterns at the micrometer scale. The SEM micrographs demonstrate significant structural diversity while maintaining certain consistent parameters. The microsphere diameter distribution follows a relatively narrow range (3.425≤d≤4.582 μm), with a mean diameter of ≈ 4.01 μm. This consistent size hints at a self‐assembly process. It is controlled by thermodynamics and depends on the balance between interfacial energy and entropy. Despite dimensional uniformity, the internal architecture demonstrates significant variability. The internal cavity diameter to overall diameter ratio varies. It goes from zero in solid spheres (Figure 14c) to 0.46 in concentric structures (Figure 14b). This shows that the self‐assembly process allows for different metastable configurations. The surface nanoparticle decoration ranges in size from 0.194 to 0.397 μm. There is a clear link between the size of the nanoparticles and the curvature of the surface below. This relationship suggests localized variations in assembly kinetics influenced by surface geometry. The higher‐order assemblies in Figure 14d–f are especially notable. They show a complex shape that emerges in new ways. The agglomerated cluster (Figure 14d) hints at a process of sequential nucleation and growth. The complex shape (Figure 14e) resembles biomimetic structures, showing complex folding patterns like those found in cortical tissue [90]. The lamellar extensions seen in Figure 14f ($l_{\mathrm{blade}}=1.232$ μm) are important. They show a shift from spherical to flat symmetry. This change may be caused by uneven interactions between aromatic rings in the L‐Phe side chains. This shape variety connects to the complex electrical behaviors seen before. It suggests that certain design features affect how information is processed. The polymorphism likely helps the system handle quick oscillations and slow, thoughtful changes in proteinoid networks.

### 3.3 Morphology of L‐Phe:L‐Lys Proteinoids

**FIGURE 15:**
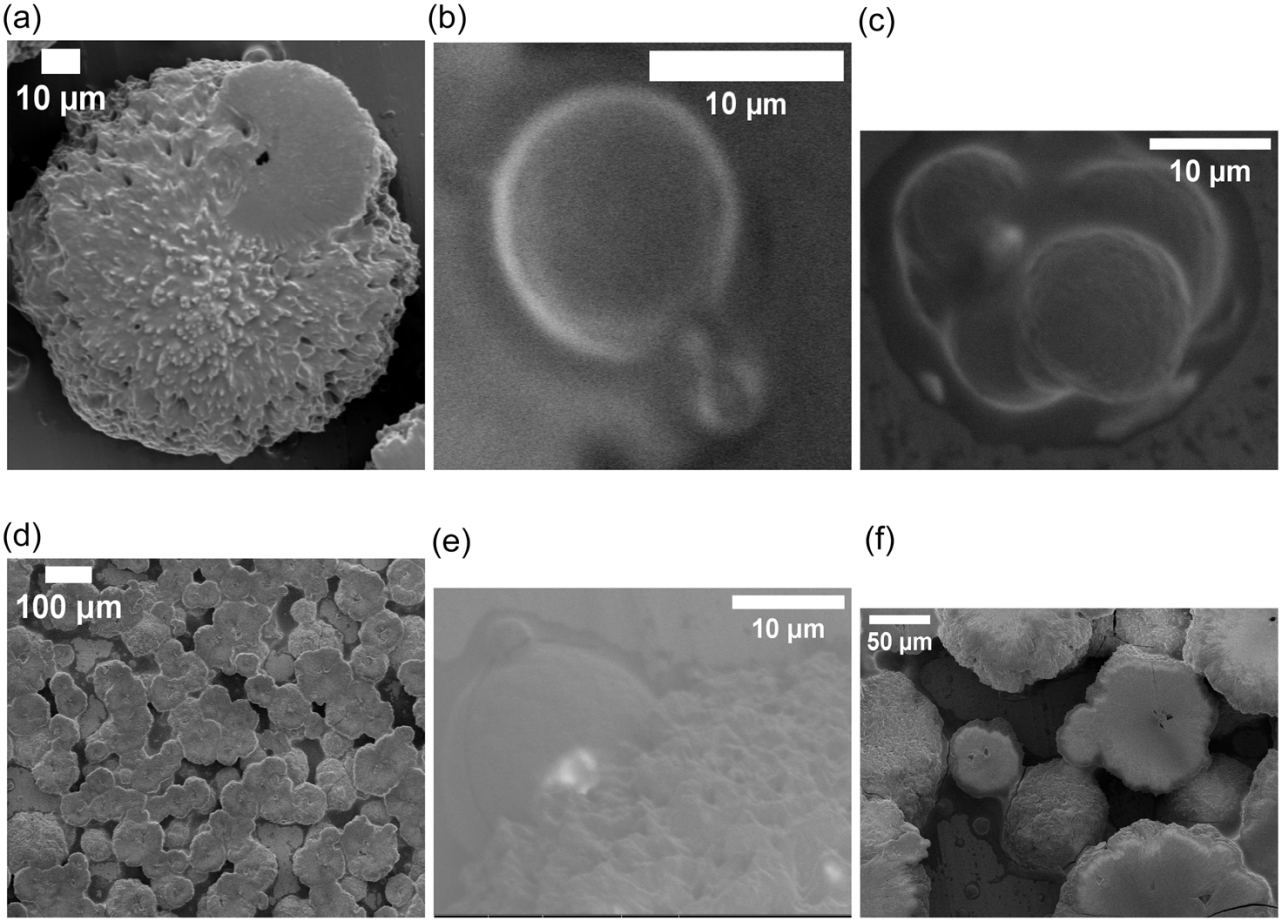
SEM micrographs revealing the hierarchical self‐assembly behavior of L‐Phe:L‐Lys proteinoid structures. (a) Unique shapes show a large crystalline lysine disk (*d* = 102.897 μm) with a textured surface. This is accompanied by a smaller lysine disk (*d* = 42.862 μm) and proteinoid microspheres (*d* = 5.798 μm). (b) An isolated PHE‐LYS crystal disk measures *d* = 11.957 μm. It shows a smooth edge and has a nascent proteinoid microsphere (*d* = 2.924 μm) budding from its surface. This hints at a possible growth mechanism. (c) A complex proteinoid assembly includes five connected microspheres. Their sizes are *d*: 14.045, 10.272, 9.994, 14.045, and 6.001 μm. This shows the system can form higher‐order structures. (d) A population view of lysine crystal disks shows diameters from 60 to 91 μm. They have a consistent shape and little size variation. High‐magnification image of a mature proteinoid microsphere (*d* = 20.006 μm) shows an emergent budding sphere (*d* = 2.741 μm). This provides evidence for vesicle‐like reproductive mechanisms. (f) Multiscale organization showing large lysine crystal disk (*d* = 129.406 μm), intermediate lysine crystal (*d* = 59.771 μm), and proteinoid microsphere (*d* = 11.652 μm), emphasizing the system's ability to form structures across multiple length scales. Scale bars: (a, b,c, e) 10 μm, (d) 100 μm, and (f) 50 μm.

Figure 15 shows the structure of L‐Phe:L‐Lys proteinoid assemblies. These assemblies have different layers and sizes, ranging across two orders of magnitude. L‐Phe:L‐Lys structures show a lot of shape variety and size differences. This is different from the more uniform microspheres seen in L‐Phe systems. The most notable feature is the mix of two different shapes: large crystalline disks and smaller spherical structures. The crystalline lysine disks are the largest in size, measuring between 12 μm and over 129 μm in diameter. Examples are disks that measure 102.897 μm (Figure 15a), 11.957 μm (Figure 15b), and 129.406 μm (Figure 15f). These disks have smooth edges and flat surfaces. This suggests anisotropic growth dynamics. This growth is likely driven by how lysine side chains are arranged. Complementing these macroscopic disks are smaller proteinoid microspheres ranging from ≈ 2.7 to 20 μm in diameter. These spherical structures appear to interact with the crystalline disks through various methods: (1) Surface association: Figure 15b shows a new microsphere (*d* = 2.924 μm) forming on a crystalline disk. This hints at possible nucleation sites at the disk's edges. (2) Hierarchical clustering: Figure 15c shows five linked microspheres. Their diameters range from 6.001 to 14.045 μm. This suggests a higher‐order organization. (3) Reproductive‐like budding: Figure 15e shows a mature microsphere (*d* = 20.006 μm) with a budding structure (*d* = 2.741 μm). This looks like vesicular reproduction methods. Figure 15d shows the population's diversity. It displays lysine crystal disks that have similar shapes. But, their diameters vary from 60 to 91 μm. Figure 15f shows how this system organizes at multiple scales. It displays structural elements that range from about 11 to 129 μm in one field of view. This analysis shows that Phe‐Lys proteinoid assemblies are much more complex than single‐amino acid systems. This happens due to the strong interaction between hydrophobic phenylalanine and positively charged lysine. The observed structure and growth patterns hint at complex functions. These may include information processing abilities that arise from these advanced assemblies.

### 3.4 Morphology of L‐Glu:L‐Arg Proteinoids

**FIGURE 16:**
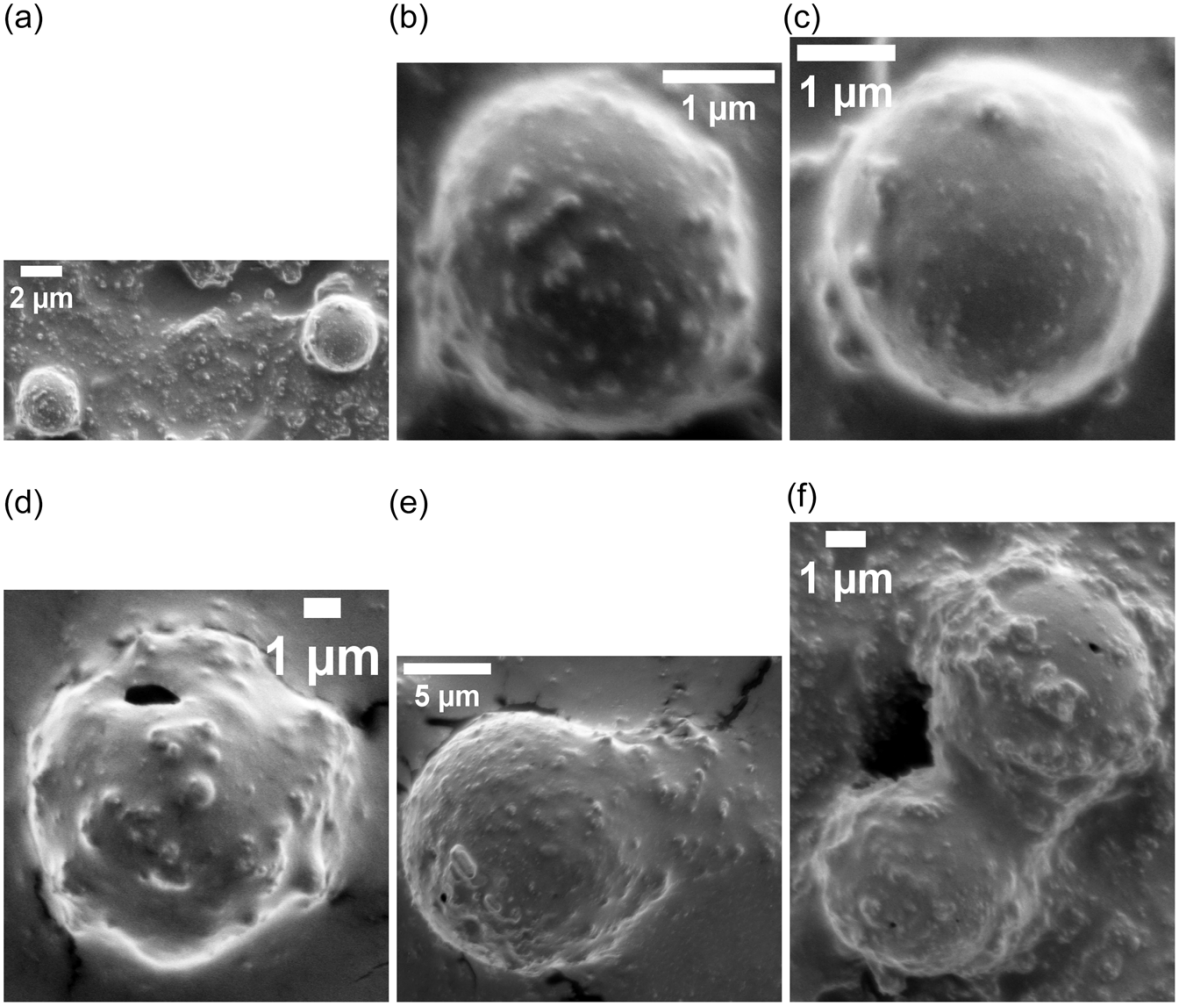
High‐resolution SEM micrographs revealing the morphological characteristics of L‐Glu:L‐Arg proteinoid microstructures. (a) Two different microspheres are present. One has a diameter of 3.424 μm and the other 2.903 μm. They are 11.182 μm apart. Also, there are small budding spheres, each 0.423 μm wide, starting to form from the substrate. (b) A mature microsphere (3.154 μm) has surface nanospheres (0.203 μm). This suggests possible nucleation sites for secondary growth. (c) A well‐formed microsphere (*d* = 3.337 μm) has a clear budding region (*d* = 0.946 μm). This shows the asymmetric growth typical of these proteinoid assemblies. (d) Larger microsphere (*d* = 8.550 μm) has a central hole (*d* = 1.461 μm) and a budding area (*d* = 0.782 μm). (e) Expanded microsphere (*d* = 14.992 μm) has a surface with a crystalline structure (*d* = 2.009 μm) and contains distributed nanospheres (*d* = 0.554 μm). This shows the system's ability to combine different shapes. (f) A fusion complex made of two microspheres (sizes: 6.101 and 4.969 μm; total size: 10.800 μm) has an interconnecting region (*d* = 3.560 μm). This shows how coalescence occurs in the evolution of proteinoid structures. Scale bars: (a) 2 μm, (b–d,f) 1 μm, and (e) 5 μm.

Figure 16 shows high‐resolution SEM images of L‐Glu:L‐Arg proteinoid microstructures. These images reveal unique self‐assembly behaviors. These behaviors are quite different from those seen in L‐Phe and L‐Phe:L‐Lys systems. The L‐Glu:L‐Arg proteinoid assemblies mainly form spherical shapes. They also show clear signs of dynamic growth.

The microspheres show two different size groups. The smaller ones range from about 2.9 to 3.4 μm (Figure 16a–c). The larger ones measure between 8.5 and 15.0 μm (Figure 16d,e). This dimensional difference suggests that there are different ways for nucleation and growth to happen. Electrostatic interactions likely impact these processes. They occur between negatively charged glutamic acid and positively charged arginine residues.

A key feature is the presence of growth dynamics shown in different structural motifs.

• Substrate nucleation: Figure 16a shows budding spheres (*d* = 0.423 μm) forming from the substrate. This gives insight into the early stages of formation.
• Surface‐mediated secondary growth: Figure 16b shows mature microspheres with nanospherical structures (*d* = 0.203 μm). These structures may act as nucleation sites for hierarchical assembly.
• Asymmetric budding: Several examples (Figure 16c,d) show clear budding areas. They measure 0.946 and 0.782 μm. This suggests directional growth, similar to how cells divide.
• The structural complexity in Figure 16e shows a larger microsphere. It has distinct surface crystallinity and contains embedded nanospheres. This highlights the system's ability to integrate multiple components.
• Fusion phenomena: Figure 16f shows clear evidence of microsphere coalescence. Two distinct structures, measuring 6.101 and 4.969 μm, are connected by a large interconnecting region of 3.560 μm.

Some structures have central cavities (Figure 16d). This suggests that assembly happens in a complex way. It may involve internal pressure differences or templating mechanisms. The L‐Phe:L‐Lys system forms clear crystalline disks. In contrast, L‐Glu:L‐Arg assemblies are spherical. They also show a lot of structural variation.

This analysis shows that L‐Glu:L‐Arg proteinoid microstructures grow in dynamic ways. This growth hints at possible new functional properties. The observed budding, fusion, and organization features show systems that can handle complex information. Their ability to adapt structurally and connect in different ways supports this.

### 3.5 Morphology of L‐Glu:L‐Phe:L‐Asp Proteinoids

**FIGURE 17:**
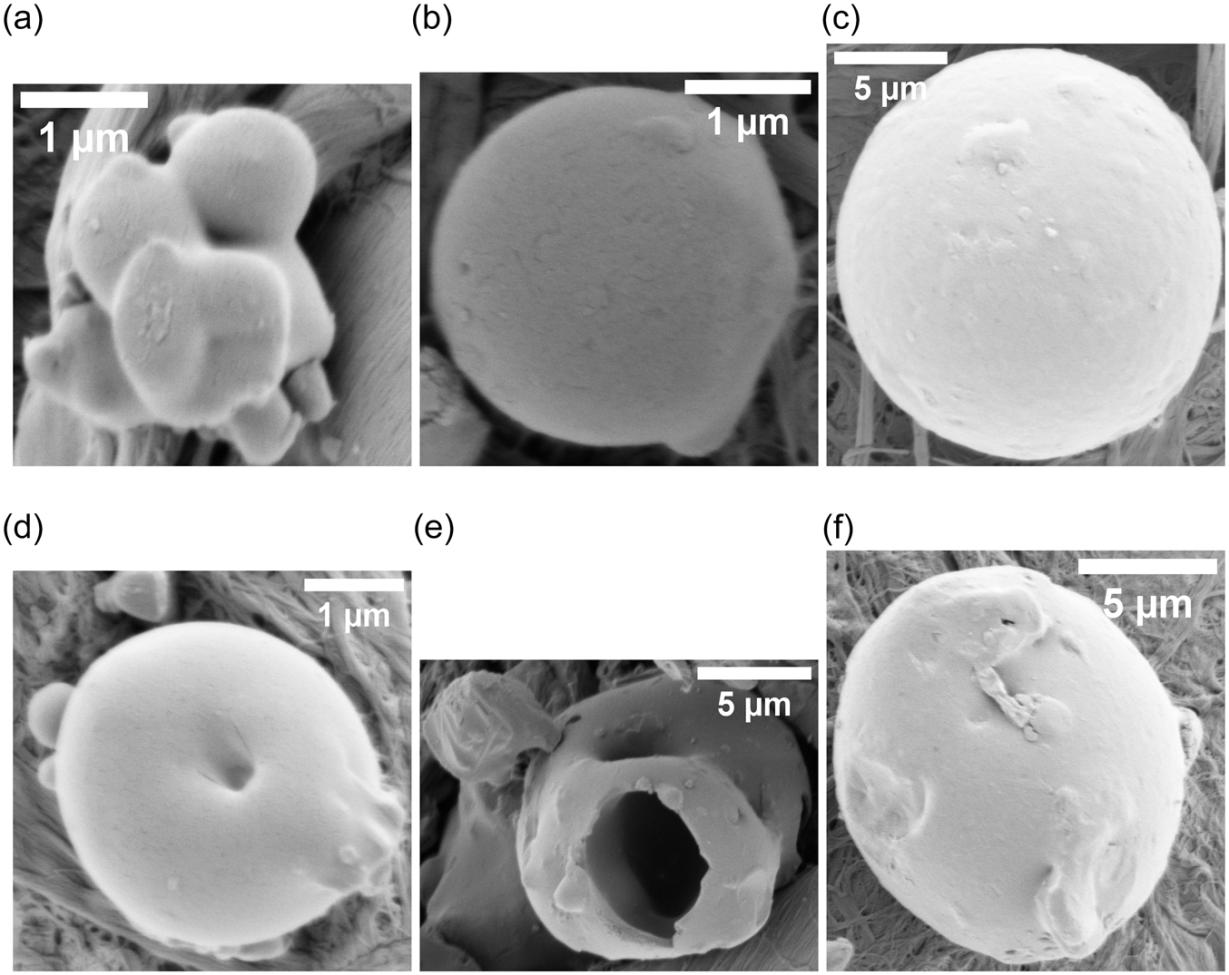
SEM characterization of L‐Glu:L‐Phe:L‐Asp proteinoid structures demonstrating diverse morphologies and self‐organization capabilities. (a) Complex assembly of interconnected microspheres (*d* = 0.899, 1.021, 1.343, 0.919, 1.000 μm) exhibiting cooperative multicellular‐like arrangement with defined junction interfaces. (b) Individual microsphere (*d* = 2.925 μm) with smooth surface morphology and initial‐stage budding formation (*d* = 0.198 μm), suggesting potential reproductive mechanism. (c) Mature large‐scale microsphere (*d* = 17.148 μm) displaying uniform spherical geometry and minimal surface irregularities, indicating stable thermodynamic equilibrium state. (d) Toroidal microsphere (*d* = 3.494 μm) with central perforation (d=0.358nm) and peripheral nanosphere budding (d=0.564nm), representing potential vesicular transition state. (e) Hollow microsphere with large aperture (*d* = 25.440 μm), substantial wall thickness (17.518 μm), outer diameter (12.536 μm), and adjacent smaller microsphere (*d* = 14.097 μm), demonstrating advanced hollow structure formation. (f) Elongated microsphere (*d* = 49.548 μm, length  = 26.590 μm) exhibiting asymmetric growth pattern with surface protrusions, suggesting directional expansion mechanisms. Scale bars: (a,b,d) 1 μm and (c,e,f) 5 μm.

Figure 17 shows the amazing variety of L‐Glu:L‐Phe:L‐Asp (GFD) proteinoid assemblies. Adding three different amino acids greatly increases the shapes beyond what we see in dipeptide systems. These tripeptide structures vary greatly in size. They range from submicron to tens of micrometers, covering almost two orders of magnitude.

The GFD proteinoid system shows a multicellular‐like organization. Figure 17a highlights this with an interconnected network of small microspheres. These microspheres range from 0.899 to 1.343 μm and have clear junction interfaces, similar to basic multicellular clusters. This spatial arrangement indicates potential cooperative interactions between adjacent structures.

Micrographs show structures at various developmental stages. They range from early budding formations (Figure 17b,d = 0.198 μm) to fully matured microspheres (Figure 17c,d = 17.148 μm). This range offers insight into growth and maturation processes.

GFD assemblies are more complex than simpler proteinoid systems. They have advanced topological features, like toroidal structures with central holes (Figure 17d). They also form large hollow microspheres with thick walls (Figure 17e). The hollow structure in Figure 17e shows a thick wall (17.518 μm) compared with its outer diameter (12.536 μm). This design shows the system can create complex internal shapes that might support different functions.

The GFD system shows great scale diversity. It ranges from tiny sub‐micron structures in clusters to large microspheres that can be nearly 50 μm in diameter (Figure 17f). The elongated microsphere in this figure measures 49.548 μm by 26.590 μm. This shape shows a clear difference from a perfect sphere. It suggests that growth happened in different ways, driven by local molecular interactions.

The complex structure of GFD proteinoids comes from the unique properties of their amino acids. For example, the negative charge of glutamic acid and aspartic acid balances the hydrophobic effects of phenylalanine. This chemical variety allows for more advanced self‐assembly. This leads to the different shapes we see.

It is interesting to see solid, hollow, and perforated structures all in the same proteinoid system. This suggests that there are different ways these structures can form and stay stable. This analysis of GFD proteinoid structures shows that more amino acid variety leads to better structure. This improvement may support complex behaviors and better information processing.

### 3.6 Morphology of L‐Asp Proteinoids

**FIGURE 18:**
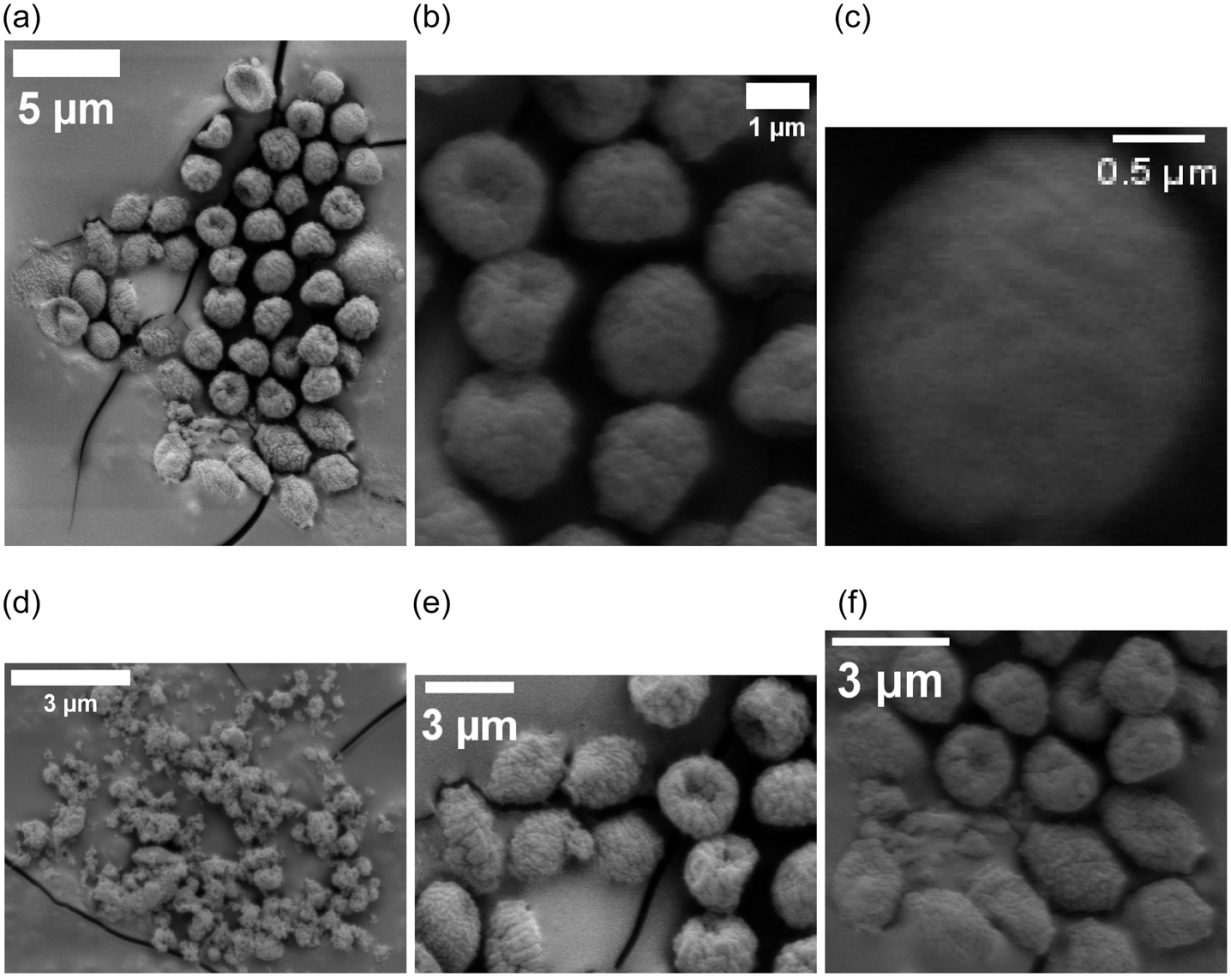
Multiscale SEM analysis of L‐aspartic acid proteinoid microsphere formations revealing hierarchical self‐assembly behavior. (a) Macroscale colony organization exhibiting densely packed microsphere assembly ($d_{\mathrm{avg}}\approx 2–3$ μm) with distinct spatial arrangement patterns and varying degrees of surface roughness, suggesting cooperative growth dynamics. Scale bar: 5 μm. (b) Higher magnification view of microsphere subpopulation revealing uniform size distribution and consistent morphology with characteristic surface texturing, indicative of stabilized growth conditions. Scale bar: 1 μm. (c) Ultrahigh resolution imaging of individual microsphere surface (*d* ≈ 0.8 μm) showing nanoscale topology with subtle undulations and potential nucleation sites for secondary growth processes. Scale bar: 0.5 μm. (d) Dispersed microsphere population (*d*_range_ ≈ 0.5–1.5 μm) demonstrating varied developmental stages with distinct surface‐to‐volume ratios (S/V), suggesting temporal evolution of assembly mechanisms. Scale bar: 3 μm. (e) Intermediate‐stage microsphere cluster with pronounced surface texturing and emergent interfacial connections ($\delta_{\mathrm{contact}}\approx 0.2–0.4$ μm) between adjacent structures, indicating potential communication pathways. Scale bar: 3 μm. (f) Advanced‐stage assembly with evidence of structural remodeling and morphological adaptation, characterized by varied geometries transitioning from spherical to quasi‐polyhedral forms ($\varphi_{\mathrm{deformation}}\approx 0.15–0.25$), possibly resulting from spatial confinement forces ($F_{\mathrm{confinement}}\propto \nabla P$). Scale bar: 3 μm. The increasing complexity seen in structures from 0.5 to 5 μm helps us understand the thermodynamic and kinetic factors behind proteinoid self‐assembly. This knowledge could be useful for biomimetic applications and models of prebiotic chemistry.

Figure 18 shows a detailed look at L‐aspartic acid proteinoid microspheres. It highlights unique assembly patterns that stand out from earlier studied amino acid systems. L‐aspartic acid proteinoids show a consistent size and shape. Their patterns are organized, which hints at a carefully controlled assembly process.

The macroscale structure in Figure 18a shows microsphere colonies that are tightly packed. These colonies have diameters that average 2–3 μm. This setup shows a pattern that suggests cells are communicating with each other. It may also indicate how they respond to their environment and organize themselves in space. These aspartic acid structures are different from more complex proteinoid systems. They have very uniform spherical shapes.

A closer look at higher magnification (Figure 18b) shows that the microspheres have a consistent shape. The smooth surface texture shows that growth conditions were controlled. This could be due to the special hydrogen bonding abilities of aspartic acid side chains. The structural homogeneity suggests a thermodynamically favored assembly pathway with minimal alternative configurations.

Figure 18c shows ultra‐high resolution imaging. This imaging gives us a clear view of the nanoscale surface details of each microsphere. The small waves seen at this scale (0.8 μm diameter) likely show how aspartic acid residues are organized at the molecular level. They may also act as starting points for growth processes.

We can see the growth of microspheres in Figure 18d. They vary in size from 0.5 to 1.5 μm and have different surface traits. This distribution shows how the assembly process changes over time. Smaller structures stand for earlier stages of development. The varying surface‐to‐volume ratios across this population provide insight into growth kinetics.

Figure 18e shows new interfaces between nearby microspheres. The contact areas are about 0.2–0.4 μm. These junction points might show new communication paths between proteinoid structures. They could allow for material exchange or signal transmission in the collective assembly.

The advanced‐stage assembly in Figure 18f shows how it adapts its shape under spatial limits. The microspheres change from round shapes to almost polyhedral ones. This happens with deformation parameters of about 0.15–0.25. This shape modulation suggests responsive mechanical properties and adaptability to environmental pressure.

The L‐aspartic acid proteinoid system is notable for its strong structural uniformity. This sets it apart from other proteinoid assemblies that have been studied before. This consistency likely comes from aspartic acid's unique properties. These include its negative charge, ability to form hydrogen bonds, and shorter side chain. These properties seem to limit morphological diversity. At the same time, they promote organized collective arrangements. This order may help develop computational or signal processing abilities due to their regular network structure.

#### 3.6.1 Comparative Analysis of Regime I Volatile Dynamics Across Proteinoid Networks

The electrical properties of various proteinoid compositions presented in Table 8 demonstrate how these systems exemplify Regime I (volatile dynamics). This dynamic regime is characterized by rapid, automatic signal transduction and immediate information processing, analogous to reflexive rather than deliberative mechanisms. The table compares five types of proteinoids: L‐Phe, L‐Phe:L‐Lys, L‐Glu:L‐Arg, L‐Glu:L‐Phe:L‐Asp, and L‐Asp. Each shows unique oscillatory behaviors and time patterns. These traits highlight their promise as models for early information processing systems.

**TABLE 8:** This table summarizes the emergent electrical properties of various proteinoid compositions, illustrating how these systems emulate the functional characteristics of Regime I (volatile dynamics). The observed spontaneous oscillatory behaviors demonstrate intrinsic computational capabilities, including temporal pattern recognition, parallel information processing, and adaptive reconfiguration. These electrical signals indicate that simple proteinoid assemblies can execute complex information handling tasks via thermodynamic mechanisms, analogous to the reflexive, precognitive operations of biological neural networks. The progressive shortening of oscillatory intervals suggests a capacity for self‐optimization, while coordinated state transitions across multiple channels provide evidence for network‐wide information integration. These findings position proteinoid microspheres as viable physical models for understanding the abiotic origins of information processing and as potential substrates for novel biomimetic computing architectures.

Proteinoid type	Key oscillatory features	Temporal dynamics	Functional implications
L‐Phe	Spontaneous bursts with decaying amplitudes (7.9, 4.27, 3.4 mV); rapid transitions (ΔV/Δ *t* = 1.129 mV/sec)	Progressive shortening of intervals (11.9, 9.67, 2.6, 2.35 min); exponential decay pattern	Automatic, parallel processing with pattern recognition; suggests adaptive learning capability
L‐Phe:L‐Lys	Confined voltage range (37–57 mV); sharp transitions (ΔV = 11.29 mV over <10 sec)	Quasi‐periodic fluctuations (8.4, 7.8, 4.0, 3.5 min); adaptive interval shortening (Δt_1_ > Δt_2_ > Δt_3_ > Δt_4_)	Demonstrates relaxation dynamics (τ constant); shows self‐organizing temporal adaptation
L‐Glu:L‐Arg	Bimodal organization with ΔVtotal ≈ 500 mV; coordinated state transitions at t ≈ 40,000 sec	Stable reference channels with dynamic responders; automatic error‐correction following perturbations	Rapid categorization into discrete potential bands; shows attention‐like amplitude modulation
L‐Glu:L‐Phe:L‐Asp	Varied landscape (−100 to 150 mV); sharp spikes at t ≈ 80,000 and 120,000 s	Stable channels (ChB, ChC) with dynamic channels (ChD, ChF); resilience with return to steady states	Dual architecture supporting parallel processing; multiple pathway integration and adaptation
L‐Asp	Dynamic range of −60 to 60 mV; Synchronized dips and recoveries at t ≈ 40,000 sec	Oscillatory patterns with decreasing amplitude; metastable states with spike events	Distributed network with specialized channels; multicellular‐like coordination and processing

The L‐Phe proteinoid shows quick bursts with decreasing amplitudes of 7.9, 4.27, and 3.4 mV. It also has rapid transitions. This suggests that it can process information automatically and in parallel, allowing it to recognize patterns over time. This is supported by shorter intervals (11.9, 9.67, 2.6, 2.35 min). These changes suggest an ability to adapt. This quality makes L‐Phe a good choice for studying self‐optimization in biomimetic systems. The L‐Phe:L‐Lys proteinoid shows a voltage range of 37–57 mV. It also has fluctuations that occur roughly every 8.4, 7.8, 4.0, and 3.5 min. This behavior shows how it adapts over time, as seen in Table 8. The system has sharp transitions, with ΔV=11.29 mV happening in under 10 s. This shows that it can process information quickly, like how our minds work intuitively.

The L‐Glu:L‐Arg proteinoid, shown in Table 8, has a bimodal structure. It features a total voltage range of about 500 mV. This indicates quick sorting into distinct potential bands. Its coordinated state changes at around 40,000 s and automatic error correction show a network that stays stable while adapting. This is like attention‐like mechanisms found in cognitive systems. The L‐Glu:L‐Phe:L‐Asp proteinoid shows a varied potential landscape from ‐100 to 150 mV. It has sharp spikes at about *t* = 80,000 s and *t* = 120,000 s. This design has stable channels (ChB, ChC) and dynamic ones (ChD, ChF). This setup is great for parallel processing and integrating pathways, as shown in Table 8. The L‐Asp proteinoid shows a dynamic range from ‐60 to 60 mV. It has synchronized dips around *t* ≈ 40,000 sec. This suggests a network with multicellular‐like coordination. It also enhances the diverse processing abilities seen in these proteinoids.

The comparative analysis presented in Table 8 demonstrates how proteinoid networks physically emulate the functional attributes of Regime I (volatile dynamics). These abiotic systems exhibit spontaneous oscillatory behaviors that are functionally isomorphic to biological neural processing, including the capacity for temporal pattern recognition, parallel signal integration, and adaptive reconfiguration. This suggests that rudimentary information processing mechanisms—such as self‐optimization through interval shortening and network‐wide information integration via coordinated state transitions—can emerge directly from molecular self‐assembly without biological machinery. These findings position proteinoid microspheres as critical models for understanding the thermodynamic origins of prebiotic cognition and as viable substrates for developing novel biomimetic computing architectures [91, 92].

### 3.7 Regime II: Nonvolatile Signal Integration and Adaptive Reconfiguration under Optical Stimulation

We employ optical stimulation protocols to characterize the nonvolatile signal integration and adaptive memory states associated with Regime II. This comes from the voltage versus time signal taken from a poppy image. Proteinoids are synthetic polypeptides that may have photosensitive properties. They receive this signal as a simulated optical input. The voltage signal goes from 0 to 5 V over 590 s. It has a 1 kHz sampling rate. This signal reflects how light intensity changes over time. This method lets us test how well proteinoids respond to complex optical patterns. We use the spatial information in the image as a changing electrical stimulus. This helps assess how proteinoids perform under these conditions. The sampling period of 1 ms is key for good temporal resolution.

#### 3.7.1 Transformation of Poppy Image to Voltage Versus Time Plot

**FIGURE 19:**
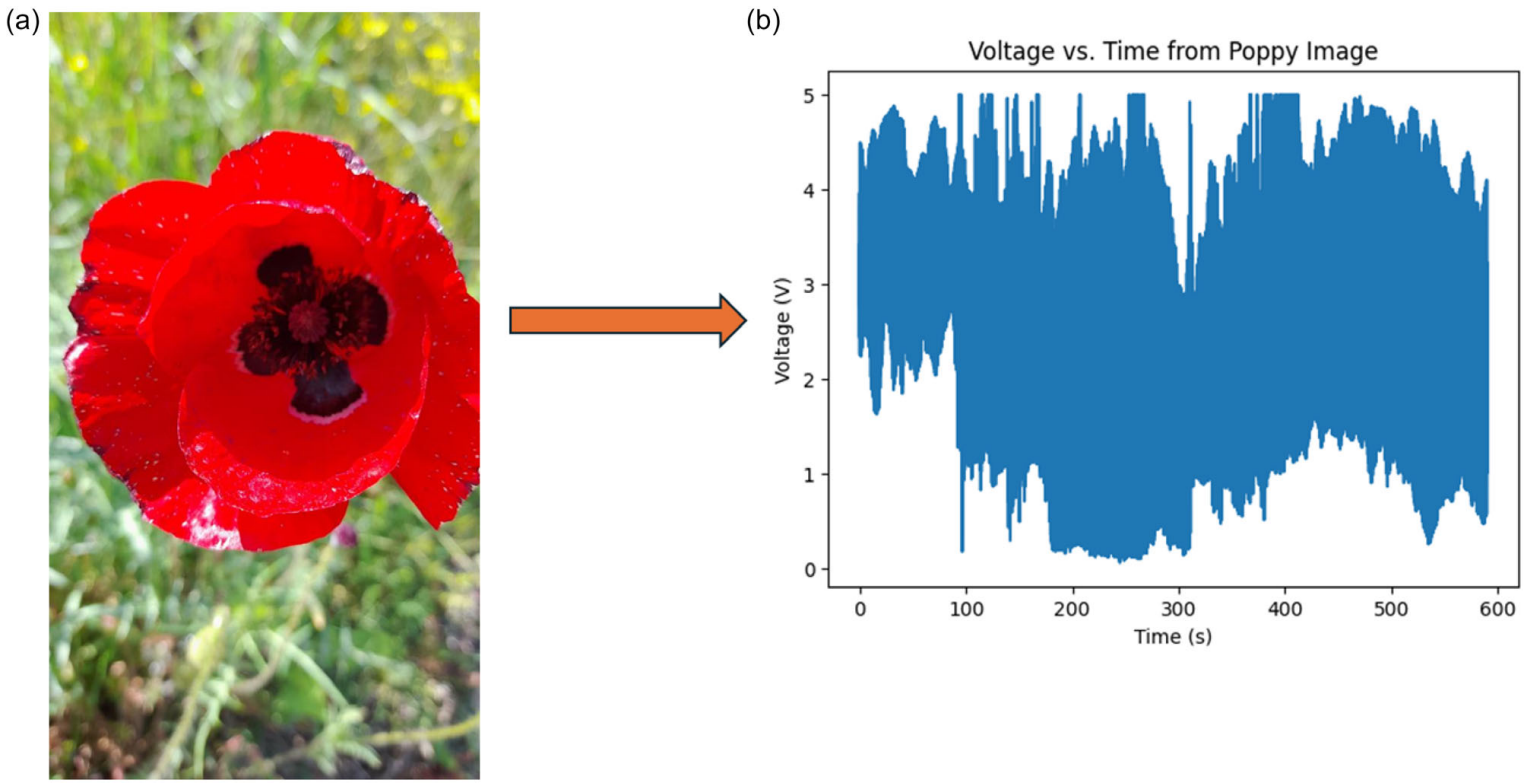
(a) Red poppy flower image used as input. (b) Resulting voltage vs. time plot, where grayscale pixel intensities are mapped to voltages (0–5 V) at a 1 kHz sampling rate, revealing spatial intensity variations as temporal fluctuations over 590 s.

Transforming the poppy image into a voltage versus time plot takes several steps. It uses image processing and signal conversion techniques. The image in Figure 19a shows a red poppy flower. It is in grayscale and set against a green background. We outline the transformation procedure as follows:

1. **Image Loading and Grayscale Conversion**: The image is loaded using the OpenCV library (cv2.imread) in grayscale mode, denoted as I(x,y), where x and y represent the pixel coordinates, and I(x,y)∈[0,255] represents the intensity value at each pixel. This is expressed as:

(21) $$I_{\text{gray}}(x,y)=\mathrm{cv}2.\mathrm{imread}(\text{'fig:poppy.png'},\mathrm{cv}2.{\mathrm{IMREAD}}_{\mathrm{GRAYSCALE}})$$

The dimensions of the image are extracted as height and width, where shape = (height,width).

2. **Flattening the Image**: The 2D grayscale image is converted into a array of intensity values by flattening it row by row. This results in a vector I of length *N* = height ⋅ width, where each element I[i] corresponds to the intensity of a pixel:

(22) $$I=\text{flatten}(I_{\text{gray}})$$

3. **Mapping Intensities to Voltage**: The intensity values, ranging from 0 to 255, are linearly mapped to a voltage range of 0–5 volts. This transformation is defined by the scaling factor $\frac{5}{255}\approx 0.0196\;\text{V per intensity unit}$. However, in the code, a division by 51 is used, which corresponds to a scaling of $\frac{255}{5}=51$ intensity units per volt, effectively mapping I[i] to V[i] as:

(23) $$V[i]=\frac{I[i]}{51},\;V[i]\in [0,5]\;V$$

This step assumes a direct proportionality between intensity and voltage, adjusted to fit the 0–5 V range.

4. **Time Axis Generation**: A time axis t is created with a sampling rate of 1 kHz, where each pixel corresponds to a time step of Δt=0.001s (1 ms). The time vector is computed as:

(24) t=[0,Δt,2Δt,…,(N−1)Δt]

where N is the total number of pixels. For the given poppy image, the total duration is ≈ 590 s, indicating N≈590,000 pixels.

5. **Plotting**: The voltage values V are plotted against the time values t using Matplotlib (plt.plot), resulting in the waveform shown in Figure 19b. The *x*‐axis represents time in seconds, and the *y*‐axis represents voltage in volts. The plot shows a complex pattern. It reflects how pixel intensities vary across the image.

### 3.8 Regime II: Statistical Quantification of Nonvolatile Responses to Optical Stimulation

The table below shows the statistical analysis of how proteinoids respond to optical stimulation from the poppy image. We analyze the input voltage from Channel B and the output potentials from Channels C to H. This is done for five proteinoid samples: L‐Asp, L‐Glu:L‐Arg, L‐Glu:L‐Phe:L‐Asp, L‐Phe, and L‐Phe:L‐Lys. We report the mean, standard deviation, and correlation with the input voltage for each channel. This gives insights into how the proteinoids respond to the optical stimulus.

**FIGURE 20:**
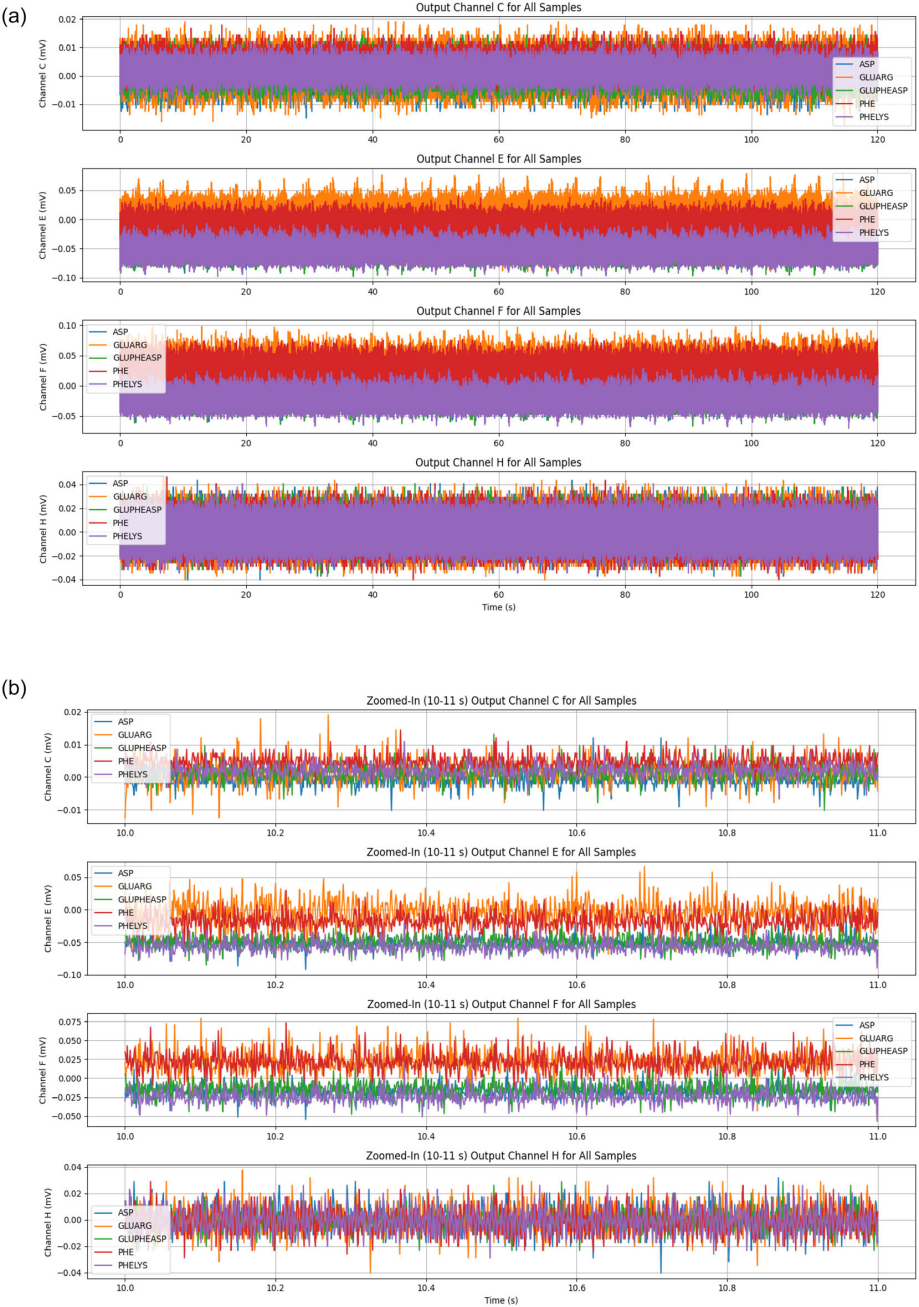
(a) Output signals for Channels C, E, F, and H from all proteinoid samples (L‐Asp, L‐Glu:L‐Arg, L‐Glu:L‐Phe:L‐Asp, L‐Phe, L‐Phe:L‐Lys) were recorded over 120 s. This was done under optical stimulation using the poppy image. (b) A close‐up view (10–11 s) of the same channels shows detailed changes and differences in how the proteinoids respond to the light stimulus.

The analysis shows clear response patterns in the proteinoid samples. They react to the optical stimulus from the poppy image. The input voltage (Channel B) stays consistent in all samples. Its mean is about 2.26 V, and the standard deviation is 1.04 V. This stability offers a reliable basis for comparison, as shown in Table 9. Channels C, E, and H show different levels of correlation with the input. Channel H has the strongest linear relationship, ranging from 0.443258 to 0.601420, especially in the L‐Asp sample at 0.601420. Table 10 shows the behavior of Channels D, F, and G. Channel F has a lot of variability in its mean response. It ranges from −0.024392 mV in L‐Phe:L‐Lys to 0.021535 mV in L‐Phe. The correlations are between 0.403606 and 0.476606. Channel G shows a steady correlation (0.419906 to 0.469194). This means it has a moderate and stable response to the optical stimulus in all samples. These results show that Channel H responds best to the optical input. Channels F and G also behave differently, highlighting the varied properties of the proteinoid samples. Figure 20 shows the output signals for Channels C, E, F, and H from all samples. This highlights how the proteinoid responses change over time. The full 120‐second time‐series in (a) shows the overall response patterns. The zoomed‐in view (10–11 s) in (b) reveals the quick changes and variability in the electrical signals. This backs up the stats shown in Tables 9 and 10.

**TABLE 9:** Statistical analysis of input voltage (Channel B) and output potentials (Channels C to H) for proteinoid samples under optical stimulation. The input voltage from the poppy image has a mean of about 2.26 V. The standard deviation is 1.04 V for all samples. This shows that the input characteristics are consistent. The output potentials vary in means, from −0.057148 to 0.021535 mV. The standard deviations range from 0.001970 to 0.022140 mV. This shows different electrical responses among the proteinoids. The correlation coefficients with the input voltage range from 0.147 to 0.601. Channel H has the highest correlation, ranging from 0.443 to 0.601. This indicates a stronger linear relationship with the optical stimulus in this channel across all samples.

Sample			Output channels, mV						
	Input voltage, V		Mean			Std Dev			**Corr.**
	**Mean**	**Std Dev**	**C**	**E**	**H**	**C**	**E**	**H**	**H**
L‐Asp	2.258309	1.042137	−0.000909	−0.051233	0.000089	0.002067	0.008002	0.009497	0.601420
L‐Glu:L‐Arg	2.257124	1.042109	0.001747	−0.003213	−0.000098	0.003423	0.019202	0.010686	0.577421
L‐Glu:L‐Phe:L‐Asp	2.258549	1.042701	0.001143	−0.049663	−0.000784	0.002501	0.010380	0.006660	0.443258
L‐Phe	2.259743	1.042980	0.004669	−0.016792	−0.000208	0.001970	0.012048	0.009747	0.561741
L‐Phe:L‐Lys	2.260538	1.042911	0.001920	−0.057148	−0.000113	0.002042	0.008970	0.009340	0.526230

**TABLE 10:** Statistical analysis of output potentials (Channels D, F, G) for proteinoid samples under optical stimulation. The output potentials range from −0.042507 to 0.021535 mV. The standard deviations vary from 0.006664 to 0.022140 mV. This shows different electrical responses among the proteinoids. Channel F varies significantly. Its means go from −0.024392 mV (L‐Phe:L‐Lys) to 0.021535 mV (L‐Phe). The correlation with input voltage ranges from 0.403606 to 0.476606. Channel G shows consistent correlations (0.419906 to 0.469194). This suggests a moderate linear relationship with the optical stimulus. Yet, it is not as strong as Channel H's.

Sample	Output channels, mV								
	**Mean**			**Std Dev**			**Corr. with Input**		
	**D**	**F**	**G**	**D**	**F**	**G**	**D**	**F**	**G**
L‐Asp	−0.037293	−0.017742	−0.035765	0.021402	0.008867	0.006664	0.327507	0.428768	0.431117
L‐Glu:L‐Arg	−0.023785	0.021387	−0.015535	0.020608	0.013779	0.006917	0.327846	0.424575	0.454402
L‐Glu:L‐Phe:L‐Asp	−0.020818	−0.014785	−0.033190	0.018825	0.010751	0.009213	0.337826	0.403606	0.424747
L‐Phe	−0.012400	0.021535	−0.001752	0.022140	0.011781	0.007020	0.335603	0.476606	0.469194
L‐Phe:L‐Lys	−0.020714	−0.024392	−0.042507	0.017943	0.009086	0.008383	0.337185	0.432862	0.419906

**FIGURE 21:**
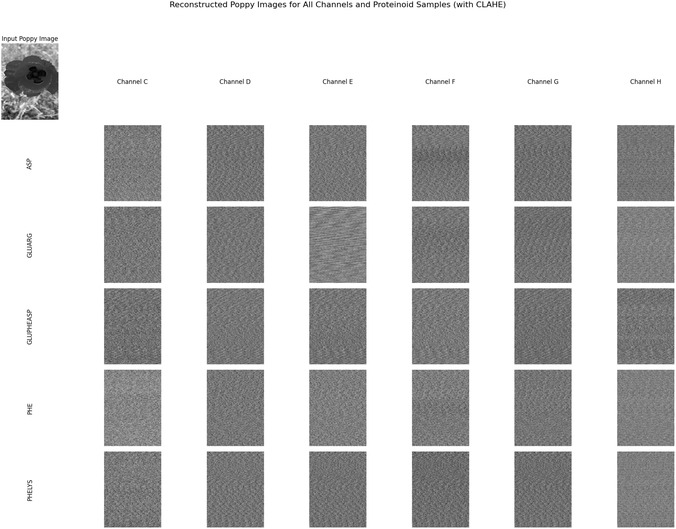
Reconstructed poppy images for Channels C, D, E, F, G, and H were made. These images came from proteinoid samples: L‐Asp, L‐Glu:L‐Arg, L‐Glu:L‐Phe:L‐Asp, L‐Phe, and L‐Phe:L‐Lys. The reconstruction occurred under optical stimulation. The input grayscale poppy image (top left) serves as the reference for the optical stimulus. The reconstructed images show limited detail. This is because the output range is small, from −0.057148 to 0.021535 mV (see Tables 9 and 10). As a result, the intensity range after mapping is narrow. Channel H has the strongest connection to the input voltage, ranging from 0.443258 to 0.601420. It shows clearer features, especially in L‐Asp and L‐Glu:L‐Arg. In contrast, Channels C and E, with lower correlations of 0.147371, create more uniform reconstructions. This points to their weaker response to the optical stimulus.

The Figure 21 displays the reconstructed poppy images for Channels C, D, E, F, G, and H. It covers all proteinoid samples: L‐Asp, L‐Glu:L‐Arg, L‐Glu:L‐Phe:L‐Asp, L‐Phe, and L‐Phe:L‐Lys. The input grayscale poppy image is used as a reference. The images come from the electrical signals we recorded after the light stimulus. We will look at how they appear alongside the statistical data from your tables.

We converted the electrical signals from optical stimulation into images (Figure 21). This showed us how each proteinoid sample saw the poppy image in Channels C to H. The poppy image, originally 1024×576 pixels, was resized to 400×300 pixels (120,000 pixels) to match the 120,000 samples recorded over 120 s at a 1 kHz sampling rate. For each channel and sample, the output potentials p (in mV) were first normalized to the 0–5 V range using a percentile‐based scaling to mitigate the impact of outliers. The 5th and 95th percentiles, $p_{\text{low}}$ and $p_{\text{high}}$, were computed, and the potentials were scaled as:

(25) $$p_{\text{norm}}=5.0\cdot \frac{p-p_{\text{low}}}{p_{\text{high}}-p_{\text{low}}}$$

with $p_{\text{norm}}$ clipped to the range [0, 5]. These normalized potentials were then mapped back to intensity values I (0–255) using the inverse of the original voltage mapping (V=I/51.0):

(26) $$I=p_{\text{norm}}\cdot 51.0$$

followed by clipping to ensure I∈[0,255]. The resulting intensities were reshaped into a 400×300 image. To improve contrast, we used Contrast Limited Adaptive Histogram Equalization (CLAHE). The clip limit was set at 2.0, and the tile grid size was 8×8. This method boosted local contrast and highlighted small differences in the reconstructed images.

The reconstructed poppy images in Figure 21 illustrate the capacity of proteinoid networks for nonvolatile signal integration under optical stimulation (Regime II). This experimental setup probes the material's ability to encode complex spatial patterns into persistent electrical states. The results demonstrate a limited fidelity in reconstructing the original image, indicating that the system operates as a low‐resolution, high‐abstraction reservoir rather than a precise optical sensor. This is clear from the similar look of the images it rebuilds across all samples and channels. This uniformity comes from the small range of electrical potentials recorded. When we map these back to intensity values, we get a narrow dynamic range. This range hides the details of the poppy image. Even with Contrast Limited Adaptive Histogram Equalization (CLAHE), the reconstructed images for Channels C, D, E, F, and G show little change. They look almost like uniform grayscale patterns. This suggests a weak response to the optical stimulus in these channels. Channel H shows clearer features, especially in the L‐Asp and L‐Glu:L‐Arg samples. Here, small contrasts hint at a stronger link to the input stimulus. This aligns with its higher correlation values. In the proteinoid samples, L‐Asp and L‐Glu:L‐Arg show slightly better results in Channel H. They reveal faint outlines that suggest the structure of the poppy image. Meanwhile, L‐Glu:L‐Phe:L‐Asp, L‐Phe, and L‐Phe:L‐Lys create more uniform outputs. This reflects their lower responsiveness. These variations suggest that the specific chemical architecture of the proteinoid assemblies—such as the electronic properties inherent to the amino acid side chains in L‐Asp and L‐Glu:L‐Arg—may significantly enhance their intrinsic photosensitivity. This elevated optoelectronic responsiveness serves to amplify the nonvolatile signal integration and memory retention capabilities defining Regime II. In contrast, other samples display weaker electrical activity, which limits their capability to capture the visual details of the poppy image.

### 3.9 Model Results for Proto‐Intelligence and Proto‐Consciousness

Table 11 shows key stats for three main network properties: complexity (permutation entropy), integration (global efficiency), and segregation (clustering coefficient). These were calculated over about 4,300 time windows for each of the five proteinoid compositions studied. The permutation entropy metric measures how unpredictable and rich the temporal patterns are in electrical signal dynamics. It shows a strong dominance of the L‐Glu:L‐Arg system, with a mean value of 0.752±0.095. This substantially exceeds all other compositions, with the next highest being L‐Glu:L‐Phe:L‐Asp at 0.554±0.059, representing a 35.8% increase in entropic complexity. The high entropy in L‐Glu:L‐Arg shows that this dipeptide creates a complex state‐space path. It moves through various electrical configurations and avoids repeating patterns. This behavior stands out because of the low standard deviation (σ=0.095). This shows that high complexity is not just occasional. Instead, it remains steady throughout the entire 50‐h recording period. The aromatic amino acid compositions (L‐Phe and L‐Phe:L‐Lys) show much lower entropies (μ≈0.52) and higher variability (σ≈0.11‐‐0.12). This means these systems shift between organized, simpler dynamics and brief bursts into more complex behavior. The complex nature of L‐Glu:L‐Arg comes from the way its charged groups interact. The negatively charged carboxyl groups of glutamic acid and the positively charged guanidinium groups of arginine create an active ionic environment. This allows for more varied charge redistribution pathways. In contrast, phenylalanine‐based systems mainly rely on hydrophobic interactions. From a proto‐consciousness view, this complex entropy fits with Integrated Information Theory (IIT). According to IIT, conscious systems need different states. They must be able to have many distinct configurations. L‐Glu:L‐Arg shows this at a chemical level.

**TABLE 11:** Comparative network topology metrics. Mean (μ) and standard deviation (σ) for three key consciousness metrics (N≈4300 windows). The data reveals a functional dichotomy between complexity and structure. L‐Glu:L‐Arg exhibits the highest Permutation Entropy (μ=0.752), indicating the richest, most unpredictable signal state space. However, the tripeptide L‐Glu:L‐Phe:L‐Asp dominates in topological architecture, showing the highest Integration (0.721) and Segregation (0.773). This specific combination—high global efficiency coupled with strong local clustering—satisfies the graph‐theoretical definition of a “Small‐World Network,” a topology associated with efficient information processing in biological brains.

**Composition**	Entropy (complexity)		Integration (global Eff.)		Segregation (clustering)	
	*μ*	*σ*	*μ*	*σ*	*μ*	*σ*
L‐Phe	0.522	0.111	0.552	0.191	0.559	0.208
L‐Phe:L‐Lys	0.516	0.119	0.553	0.194	0.555	0.222
**L‐Glu:L‐Arg**	**0.752**	0.095	0.655	0.223	0.706	0.207
**L‐Glu:L‐Phe:L‐Asp**	0.554	0.059	**0.721**	0.185	**0.773**	0.153
L‐Asp	0.536	0.083	0.527	0.202	0.534	0.225

L‐Glu:L‐Arg is complex, but Table 11 shows that the tripeptide L‐Glu:L‐Phe:L‐Asp leads in topological sophistication. It has the highest integration (0.721±0.185) and segregation (0.773±0.153) values of all tested compositions. This dual optimization is important. High integration, or global efficiency, means that electrical signals move quickly through the network. This reduces resistance along the paths. As a result, the system can coordinate better and allows for what neuroscientists call “global broadcasting” of information [84, 85, 87]. High segregation shows that the network has strong local modular structures. These are tightly connected subgraphs. They can perform specialized tasks in parallel. Then, they contribute to the overall state. These properties together fit the formal definition of a small‐world network topology. Watts and Strogatz first identified this structure in social networks in 1998 [76]. It is now seen as a key principle in biological neural systems, from *C. elegans* to the human cortex [86, 93, 94]. Small‐world architectures are efficient. They lower wiring costs by using fewer long‐range connections. At the same time, they boost information transfer with shorter average path lengths. This design also supports functional specialization with high clustering. The L‐Glu:L‐Phe:L‐Asp system's topology is not easy to achieve. It needs a careful balance. Local connectivity comes from molecular interactions in microsphere clusters. Global integration relies on long‐range ionic currents or electrotonic coupling across the proteinoid field. The low standard deviations (σ=0.185 for integration, σ=0.153 for segregation) show that this small‐world setup is strongly maintained over time. It's not a temporary effect. L‐Asp has the lowest and most varied topology metrics. Its integration is μ=0.527 and segregation is μ=0.534, both with σ>0.20. This suggests a fragmented, less coherent network. These findings match SEM observations of tightly packed microspheres, which lack the organized structure seen in tripeptide systems. The small‐world layout of L‐Glu:L‐Phe:L‐Asp supports Regime II‐like thinking. It allows quick integration of local computations, like how brain regions work together. This structure helps form clear decisions while responding efficiently to stimuli in real‐time.

**FIGURE 22:**
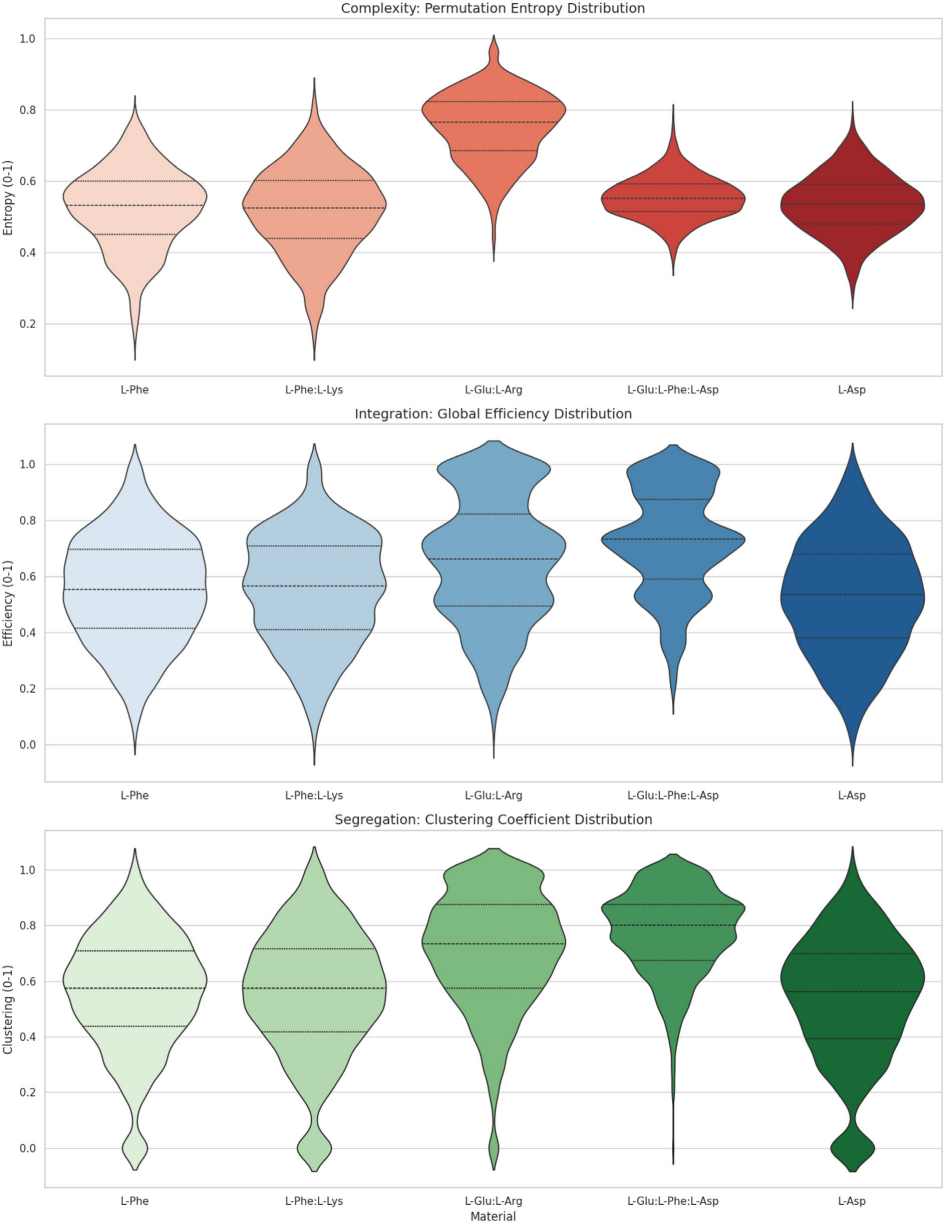
Comparative topological architecture of proteinoid networks. These violin plots illustrate the probability density distributions of three key graph‐theoretical metrics across all five proteinoid compositions (N≈4300 windows per sample). **(Top) Complexity**: The Permutation Entropy distribution reveals that L‐Glu:L‐Arg (orange) maintains the highest and most consistent signal complexity (μ≈0.8), indicating a sophisticated, nonrepetitive state space. In contrast, L‐Phe (light pink) exhibits a broader, lower‐entropy distribution, suggesting simpler periodic dynamics. **(Middle) Integration**: Global Efficiency measures the capacity for network‐wide information flow. L‐Glu:L‐Arg and L‐Glu:L‐Phe:L‐Asp (blue/teal) display top‐heavy distributions, confirming their superior ability to integrate signals across the entire macroscopic structure (“Global Broadcasting”). **(Bottom) Segregation**: The Clustering Coefficient quantifies local modularity. L‐Glu:L‐Phe:L‐Asp (green) shows a tightly clustered distribution at high values (≈0.8), indicating a stable, modular architecture. Conversely, L‐Asp (dark green) exhibits a massive dynamic range (spanning 0.2 to 1.0), reinforcing the “multicellular” hypothesis where the network continuously reconfigures between segregated local processing and global unison.

Figure 22 builds on the stats in Table 11. It shows the full probability density distributions for each metric across about 4,300 temporal windows. This visualization highlights specific signatures that mean‐and‐standard‐deviation summaries might miss. In the Complexity panel (top), the L‐Glu:L‐Arg distribution (orange violin) has a tall, narrow shape centered around 0.8. This shows the system works in a high‐entropy state, rarely shifting to simpler dynamics. This is a ”constitutively complex” phenotype. L‐Phe (light pink) shows a wider, bimodal distribution from 0.3 to 0.7. This suggests it switches between simpler periodic oscillations and more complex chaotic regimes. This aligns with the ”fast‐slow” dual dynamics seen in Figure 2. The Integration panel (middle) shows that both L‐Glu:L‐Arg and L‐Glu:L‐Phe:L‐Asp (orange and teal violins) have high global efficiency values (>0.7). However, L‐Glu:L‐Phe:L‐Asp has a tighter distribution with less variance. This means it has more consistent long‐range connectivity. This stability might explain why L‐Glu:L‐Phe:L‐Asp reconstructions (Figure 16) show clearer spatial features. They have lower entropy, but reliable pathways help signals travel well, even if the dynamics are simpler. The Segregation panel (bottom) shows L‐Asp's uneven distribution. Its mean clustering coefficient is 0.534, which seems normal. However, the violin plot displays a wide range from 0.2 to 1.0. There's notable density at both ends.

L‐Asp networks change their structure over time. They shift between two main states:

• **Highly modular states** (clustering ≈1.0) with isolated microsphere clusters.
• **Globally connected states** (clustering ≈0.2) where local structure breaks down.

The observed oscillation between high and low clustering coefficients reflects changes in the network's functional connectivity, meaning that the strength and effective range of electrical coupling between microspheres vary over time. The SEM image (Figure 18) confirms a stable underlying morphological structure; however, the electrical communication topology of the system evolves dynamically despite this structural stability.

In highly modular states (clustering ≈1.0), electrical signals propagate within local clusters of nearby microspheres, while coupling between clusters remains weak. In contrast, during globally connected states (clustering ≈0.2), transient ionic currents or enhanced capacitive coupling enable signals to travel over longer distances, spreading across the proteinoid field and temporarily disrupting its modular organization. These transitions occur on timescales ranging from minutes to hours, as revealed by sliding‐window analysis using 1,000 samples at a sampling rate of 1 Hz. They are driven by several electrochemical processes, including the redistribution of mobile charge carriers within the proteinoid matrix, changes in local ionic strength or pH at microsphere interfaces, and the accumulation and dissipation of electrical potentials that modulate junction conductance. In addition, possible conformational adjustments of the peptide backbone may alter electrostatic interactions without requiring physical movement of the microspheres. This behavior closely parallels functional dynamics observed in biological neural networks, where anatomical connectivity remains fixed, while synaptic strengths vary, allowing adaptive modulation of information flow without structural rewiring. The L‐Asp proteinoid system therefore exhibits a form of network‐level functional plasticity [95, 96].

This dynamic range backs the ”multicellular‐like coordination” idea from the morphological analysis (Figure 13). Dense‐packed L‐Asp microspheres can briefly sync electrical activity over wide areas. Then, they return to localized, independent oscillations. These distribution features show that proteinoid network topology is not fixed. It can change dynamically. The chemistry involved sets the basic structure, like whether it is small‐world or random. It also affects the range of topological states available. The close distributions of L‐Glu variants show a strong, reliable system for information processing (Regime II stability). In contrast, the wide distributions of L‐Asp and L‐Phe suggest flexible networks that can quickly change states (Regime I reactivity). This gives a solid foundation for the dual‐regime thermodynamic framework discussed in this work.

## 4 Conclusion

This study shows that proteinoid microspheres are key to understanding how information processing began in early environments. We show that these abiotic polypeptide assemblies can spontaneously form dual‐regime computational structures. This happens without needing genetic material or cellular machinery. Our analysis includes electrical, morphological, and information‐theoretic methods. Our key findings show that proteinoid networks allow fast signal transfer (Regime I/volatile dynamics) and long‐lasting memory creation (Regime II/nonvolatile Integration). This all happens through thermodynamic processes. The observed electrical signatures show that information processing happens through the interaction of molecular self‐assembly and environmental factors. Adaptive oscillatory patterns appear in L‐Phe systems with decreasing intervals. In L‐Glu:L‐Arg networks, there is a bimodal voltage change of 500 mV. Correlation profiles under optical stimulation range from *r* = 0.443 to 0.601. These findings highlight the complexity of these systems. Critically, the discovered structure‐function relationships establish a quantitative link between morphological complexity and computational capacity. The small‐world network of the tripeptide L‐Glu:L‐Phe:L‐Asp has a global efficiency of 0.721 and a clustering coefficient of 0.773. This setup reflects how biological neural networks are organized, even though it is maintained through nonbiological methods. This convergence means that efficient information processing setups might be favored by thermodynamics. They likely come from energy dissipation limits instead of being shaped by evolution. This framework for proto‐intelligence and proto‐consciousness offers new ways to measure cognitive‐like traits in prebiotic systems. High algorithmic complexity (CLZ ≈ 12.14) and dynamic spatial coherence (Isync from 0.25 to 0.95) show that proteinoids hold rich information states. They also flexibly change their functional connectivity. This flexibility is key for metastable dynamics, which are important for adaptive behavior. These findings impact three areas: origins of life research, wherethey show that the path from chemistry to cognition may happen through natural stages, not just random molecular events; unconventional computing, where they identify proteinoids as a new type of soft matter processor that can handle memory and computation without needing silicon or biological parts; and synthetic biology, where they offer design principles for creating adaptive, self‐organizing materials with new computational abilities. Future studies should look into how proteinoid networks can scale. They should also examine how these networks learn from repeated stimulation. Lastly, researchers need to explore how to use specific amino acid compositions for certain tasks. Simple molecular assemblies can create a dual‐process system for biological cognition. This changes how we view intelligence. It shows intelligence as a property that emerges from matter not in equilibrium.

## Funding

This study was supported by the Engineering and Physical Sciences Research Council (EP/W010887/1 “Computing with proteinoids").

## Conflicts of Interest

The authors declare no conflicts of interest.

## Data Availability

The data for the study is available online and can be accessed at https://zenodo.org/records/15069273.

## References

[1] A. Adamatzky , “Towards Proteinoid Computers. Hypothesis Paper,” Biosystems 208 (2021): 104480. 34265376 10.1016/j.biosystems.2021.104480

[2] R. Egel , “Origins and Emergent Evolution of Life: The Colloid Microsphere Hypothesis Revisited,” Origins of Life and Evolution of Biospheres 44 (2014): 87–110. 10.1007/s11084-014-9363-8 25208738

[3] M. Kolesnikov , “Proteinoid Microspheres and the Process of Prebiological Photophosphorylation,” Origins of Life and Evolution of the Biosphere 21 (1991): 31–37.

[4] K. Matsuno , Encyclopedia of Astrobiology (Springer, 2023), 2480–2481.

[5] P. Mougkogiannis and A. Adamatzky , “Proteinoid Microspheres as Protoneural Networks,” ACS Omega 8 (2023): 35417–35426. 37780014 10.1021/acsomega.3c05670 PMC10536103

[6] P. Mougkogiannis and A. Adamatzky , “Learning in Ensembles of Proteinoid Microspheres,” Royal Society Open Science 10 (2023): 230936. 37830018 10.1098/rsos.230936 PMC10565364

[7] T. Nakashima , “Metabolism of Proteinoid Microspheres,” in Organic Geo- and Cosmochemistry, Topics in Current Chemistry 139 (Springer, 2005): 57–81. 10.1007/BFb0018078 11542055

[8] S. W. Fox , J. R. Jungck , and T. Nakashima , “From Proteinoid Microsphere to Contemporary Cell: Formation of Internucleotide and Peptide Bonds by Proteinoid Particles,” Origins of Life 5 (1974): 227–237. 4842072

[9] J. G. Miller , “Living Systems: Basic Concepts,” Behavioral Science 10 (1965): 193–237. 5318173 10.1002/bs.3830100302

[10] J. S. B. T. Evans , “Intuition and Reasoning: A Dual‐Process Perspective,” Psychological Inquiry 21 (2010): 313–326.

[11] B. E. Jonathan , International Handbook of Thinking and Reasoning (Routledge, 2017), 151–166.

[12] D. Kahneman , Thinking Fast and Slow (macmillan, 2011).

[13] P. Mougkogiannis and A. Adamatzky , “On the Response of Proteinoid Ensembles to Fibonacci Sequences,” ACS Omega 10 (2025): 10401–10424. 40124033 10.1021/acsomega.4c10571 PMC11923683

[14] D. Tatar , S. Harrison , M. Stewart , C. Frisina , and P. Musaeus , “Proto‐Computational Thinking: The Uncomfortable Underpinnings,” in Emerging Research, Practice, and Policy on Computational Thinking (Springer, 2017), 63–81.

[15] J.‐P. Bouchaud , “Anomalous Relaxation in Complex Systems: From Stretched to Compressed Exponentials,” Anomalous Transport: Foundations and Applications (2008): 327–345.

[16] P. Sterling and S. Laughlin , Principles of Neural Design (MIT press, 2015).

[17] Y. Gokcekuyu , F. Ekinci , M. S. Guzel , K. Acici , S. Aydin , and T. Asuroglu , ”Artificial Intelligence in Biomaterials: A Comprehensive Review,“ Applied Sciences 14 (2024): 6590.

[18] K. Ruiz‐Mirazo , C. Briones , and A. de la Escosura , “Prebiotic Systems Chemistry: New Perspectives for the Origins of Life,” Chemical Reviews 114 (2014): 285–366. 24171674 10.1021/cr2004844

[19] A. Adamatzky , Unconventional computing (Taylor & Francis, 2014).

[20] P. Schwille , “Bottom‐up Synthetic Biology: Engineering in a Tinkerer's World,” Science 333 (2011): 1252–1254. 21885774 10.1126/science.1211701

[21] A. Eschenmoser , “The Search for the Chemistry of Life's Origin,” Tetrahedron 63 (2007): 12821–12844.

[22] S. L. Miller , “A Production of Amino Acids under Possible Primitive Earth Conditions,” Science 117 (1953): 528–529. 13056598 10.1126/science.117.3046.528

[23] D. L. Rohlfing , “Coacervate‐Like Microspheres From Lysine‐Rich Proteinoid,” Origins of Life 6 (1975): 203–209. 239375 10.1007/BF01372406

[24] S. W. Fox , The Origin of Life and Evolutionary Biochemistry (Springer, 1974), 119–132.

[25] A. I. Oparin , The Origin of Life on the Earth (CABI Publishing (CABI Digital Library), 1957).

[26] M. Neveu , H.‐J. Kim , and S. A. Benner , “The “Strong” RNA World Hypothesis: Fifty Years Old,” Astrobiology 13 (2013): 391–403. 23551238 10.1089/ast.2012.0868

[27] D. M. Lilley , ”The Origins of RNA Catalysis in Ribozymes,“ Trends in Biochemical Sciences 28 (2003): 495–501. 13678961 10.1016/S0968-0004(03)00191-9

[28] S. W. Fox , ”Others Simulated Natural Experiments in Spontaneous Organization of Morphological Units From Proteinoid,” in Origins of Prebiological Systems and of Their Molecular Matrices (Academic Press, 1965), 361–382.

[29] S. W. Fox and K. Harada , “Thermal Copolymerization of Amino Acids to a Product Resembling Protein,” Science 128 (1958): 1214–1214. 13592311 10.1126/science.128.3333.1214

[30] L. E. Orgel , “The Origin of Life on the Earth,” Scientific American 271 (1994): 76–83. 10.1038/scientificamerican1094-76 7524147

[31] R. Lohrmann and L. Orgel , “Prebiotic Synthesis: Phosphorylation in Aqueous Solution,” Science 161 (1968): 64–66. 5655266 10.1126/science.161.3836.64

[32] S. W. Fox , “The Origins of Behavior in Macromolecules and Protocells,” Comparative Biochemistry and Physiology Part B: Comparative Biochemistry 67 (1980): 423–436.

[33] L. Raggi , J. L. Bada , and A. Lazcano , “On the Lack of Evolutionary Continuity between Prebiotic Peptides and Extant Enzymes,” Physical Chemistry Chemical Physics 18 (2016): 20028–20032. 27121024 10.1039/c6cp00793g

[34] I. Fry , “Are the Different Hypotheses on the Emergence of Life as Different as They Seem?,” Biology and Philosophy 10 (1995): 389–417.

[35] M. Morange , A History of Molecular Biology (Harvard University Press, 2000).

[36] S. Islam and M. W. Powner , “Prebiotic Systems Chemistry: Complexity Overcoming Clutter,” Chem 2 (2017): 470–501.

[37] P. Mougkogiannis and A. Adamatzky , “Logical Gates in Ensembles of Proteinoid Microspheres,” Plos One 18 (2023): e0289433. 37721941 10.1371/journal.pone.0289433 PMC10506713

[38] W. J. Freeman , “Chaos in the Brain: Possible Roles in Biological Intelligence,” International Journal of Intelligent Systems 10 (1995): 71–88.

[39] A. Trewavas , “Aspects of Plant Intelligence,” Annals of Botany 92 (2003): 1–20. 12740212 10.1093/aob/mcg101 PMC4243628

[40] A. Adamatzky , S. Harding , V. Erokhin , et al., Inspired by Nature: Essays Presented to Julian F. Miller on the Occasion of His 60th Birthday (Springer, 2017), 357–387.

[41] A. Adamatzky , J. Vallverdu , A. Gandia , A. Chiolerio , O. Castro , and G. Dodig‐Crnkovic , Fungal Machines: Sensing and Computing with Fungi (Springer, 2023), 409–422.

[42] P. Mougkogiannis , A. Nikolaidou , and A. Adamatzky , ”Kombucha–Proteinoid Crystal Bioelectric Circuits,“ ACS Omega 9 (2024): 45386–45401. 39554456 10.1021/acsomega.4c07319 PMC11561624

[43] P. Mougkogiannis , A. Nikolaidou , and A. On Adamatzky , “Emergence of Spontaneous Oscillations in Kombucha and Proteinoids,” BioNanoScience 15 (2025): 1–27. 10.1007/s12668-024-01678-5 PMC11835939 39980746

[44] A. Nikolaidou , P. Mougkogiannis , and A. Adamatzky , “Living Kombucha Electronics with Proteinoids,” ACS Omega 10 (2025): 21128–21146. 40488052 10.1021/acsomega.4c09743 PMC12138622

[45] P. Mougkogiannis , A. Nikolaidou , and A. Adamatzky , “Light‐Induced Spiking Response in Proteinoid–actin–kombucha System,” Materials Advances 5 (2024): 9061–9091.

[46] P. Mougkogiannis , A. Nikolaidou , and A. Adamatzky , “On Transducing Properties of Kombucha–proteinoid Complexes,” ACS Applied Bio Materials 7 (2024): 4725–4746. 10.1021/acsabm.4c00535 PMC11253094 38898668

[47] P. Mougkogiannis and A. Adamatzky , “Learning in Kombucha,” Next Materials 9 (2025): 101281.

[48] D. Sahoo and J. Seckbach , “ *The Algae World* ,” Cellular Origin, Life in Extreme Habitats and Astrobiology (Springer Nature, 2015).

[49] Y. Yang and Y. Shen , “A Liquid Metal‐Based Module Emulating the Intelligent Preying Logic of Flytrap,” Nature Communications 15 (2024): 3398. 10.1038/s41467-024-47791-7 PMC11035631 38649382

[50] C. I. Abramson and A. M. Chicas‐Mosier , “Learning in Plants: Lessons From Mimosa Pudica,” Frontiers in Psychology 7 (2016): 417. 27065905 10.3389/fpsyg.2016.00417 PMC4814444

[51] C. L. Fermaniuk , The Smart Plant: A Look into the Controversy behind Plant Intelligence (MacEwan University Student eJournal, 2020), 4.

[52] M. D. Fricker , D. Bebber , and L. Boddy , “Mycelial Networks: Structure and Dynamics,” British Mycological Society Symposia Series (Elsevier, 2008), 3–18.

[53] A. Adamatzky , P. Ayres , A. E. Beasley , N. Roberts , and H. A. Wösten , “Logics in Fungal Mycelium Networks,” Logica Universalis 16 (2022): 655–669.

[54] A. L. Castro‐Delgado , S. Elizondo‐Mesén , Y. Valladares‐Cruz , and W. Rivera‐Méndez , “Wood Wide Web: Communication through the Mycorrhizal Network,” Revista Tecnología en Marcha 33 (2020): 114–125.

[55] P. Mougkogiannis , A. Nikolaidou , and A. Adamatzky , “Living Electronics in Cellulose Zoogleal Mats,” Carbohydrate Polymer Technologies and Applications 9 (2024): 100627.

[56] P. Mougkogiannis and A. Adamatzky , “Scale‐Dependent Analysis of Structure and Electrical Activity in Kombucha Mats and Proteinoid–actin Assemblies,” International Journal of Biological Macromolecules 322 (2025): 146385. 40796039 10.1016/j.ijbiomac.2025.146385

[57] A. Nikolaidou , A. Chiolerio , M. M. Dehshibi , and A. Adamatzky , “Functionalizing the Electrical Properties of Kombucha Zoogleal Mats for Biosensing Applications,” ACS Omega 9 (2024): 30308–30320. 39035971 10.1021/acsomega.4c01227 PMC11256297

[58] H. Harz , C. Nonnengasser , and P. Hegemann , “The Photoreceptor Current of the Green Alga Chlamydomonas,” Philosophical Transactions of the Royal Society of London. Series B: Biological Sciences 338 (1992): 39–52.

[59] P. Mougkogiannis and A. Adamatzky , “Biohybrid Computing with Proteinoids and Algae,” Advanced Science 12 (2025): e06155. 40652521 10.1002/advs.202506155 PMC12463021

[60] K. C. Leptos , M. Chioccioli , S. Furlan , A. I. Pesci , and R. E. Goldstein , “Phototaxis of Chlamydomonas Arises From a Tuned Adaptive Photoresponse Shared with Multicellular Volvocine Green Algae,” Physical Review E 107 (2023): 014404. 36797913 10.1103/PhysRevE.107.014404 PMC7616094

[61] N. P. Money , “Hyphal and Mycelial Consciousness: The Concept of the Fungal Mind,” Fungal Biology 125 (2021): 257–259. 33766303 10.1016/j.funbio.2021.02.001

[62] M. T. Turvey , C. Carello , and C. Carello , “On Intelligence From First Principles: Guidelines for Inquiry into the Hypothesis of Physical Intelligence (PI,” Ecological Psychology 24 (2012): 3–32.

[63] J. J. Gibson , The Ecological Approach to Visual Perception (1979).

[64] E. G. Horn , “Food Competition among the Cellular Slime Molds (Acrasieae),” Ecology 52 (1971): 475–484.

[65] S. K. Ray , G. Valentini , P. Shah , et al., “Information Transfer during Food Choice in the Slime Mold Physarum Polycephalum,” Frontiers in Ecology and Evolution 7 (2019): 67.

[66] F. Jabr and A. Rothschild , “How Brainless Slime Molds Redefine Intelligence,” Nature 7 (2012).

[67] A. Adamatzky and T. Schubert , “Slime Mold Microfluidic Logical Gates,” Materials Today 17 (2014): 86–91.

[68] R. Mayne , A. Adamatzky , and J. Jones , “On the Role of the Plasmodial Cytoskeleton in Facilitating Intelligent Behavior in Slime Mold Physarum Polycephalum,” Communicative & Integrative Biology 8 (2015): e1059007. 26478782 10.1080/19420889.2015.1059007 PMC4594612

[69] X.‐Y. Huang , “The Organization of Moist Convection by Internal Gravity Waves,” Tellus A: Dynamic Meteorology and Oceanography 42 (2022): 270–285.

[70] G. Beintema , A. Corbetta , L. Biferale , and F. Toschi , “Controlling Rayleigh–Bénard Convection via Reinforcement Learning,” Journal of Turbulence 21 (2020): 585–605.

[71] S. A. Barab , M. Cherkes‐Julkowski , R. Swenson , S. Garrett , R. E. Shaw , and M. Young , “Principles of Self‐Organization: Learning as Participation in Autocatakinetic Systems,” Journal of the Learning Sciences 8 (1999): 349–390.

[72] A. Kleidon , Y. Malhi , and P. M. Cox , “Maximum Entropy Production in Environmental and Ecological Systems,” Philosophical Transactions of the Royal Society B: Biological Sciences 365 (2010): 1297–1302. 10.1098/rstb.2010.0018 PMC2871911 20368247

[73] B. Merker , K. Williford , and D. Rudrauf , “The Integrated Information Theory of Consciousness: A Case of Mistaken Identity,” Behavioral and Brain Sciences 45 (2022): e41. 10.1017/S0140525X21000881 34006338

[74] B. J. Thomas , M. A. Riley , and J. B. Wagman , Perception as Information Detection (Routledge, 2019), 237–252.

[75] J.‐P. Lachaux , E. Rodriguez , J. Martinerie , and F. J. Varela , “Measuring Phase Synchrony in Brain Signals,” Human Brain Mapping 8 (1999): 194–208. 10619414 10.1002/(SICI)1097-0193(1999)8:4<194::AID-HBM4>3.0.CO;2-C PMC6873296

[76] D. J. Watts and S. H. Strogatz , ”Collective Dynamics of Small‐Worldnetworks,“ Nature 393 (1998): 440–442. 9623998 10.1038/30918

[77] A. Hagberg , P. J. Swart , and D. A. Schult , Exploring Network Structure, Dynamics, and Function Using NetworkX,” in Proceedings of the Python in Science Conference (U.S. Department of Energy, Office of Scientific and Technical Information (OSTI), 2008).

[78] M. Oizumi , L. Albantakis , and G. Tononi , “From the Phenomenology to the Mechanisms of Consciousness: Integrated Information Theory 3.0.,” PLoS Computational Biology 10 (2014): e1003588. 24811198 10.1371/journal.pcbi.1003588 PMC4014402

[79] G. Tononi , “An Information Integration Theory of Consciousness,” BMC Neuroscience 5 (2004): 42. 15522121 10.1186/1471-2202-5-42 PMC543470

[80] G. Tononi , M. Boly , M. Massimini , and C. Koch , “Integrated Information Theory: From Consciousness to Its Physical Substrate,” Nature Reviews Neuroscience 17 (2016): 450–461. 27225071 10.1038/nrn.2016.44

[81] A. G. Casali , O. Gosseries , M. Rosanova , et al., “A Theoretically Based Index of Consciousness Independent of Sensory Processing and Behavior,” Science Translational Medicine 5 (2013): 198ra105–198ra105. 10.1126/scitranslmed.3006294 23946194

[82] C. Bandt and B. Pompe , “Permutation Entropy: A Natural Complexity Measure for Time Series,” Physical Review Letters 88 (2002): 174102. 12005759 10.1103/PhysRevLett.88.174102

[83] H. Kantz and T. Schreiber , Nonlinear Time Series Analysis (Cambridge University Press, 2003).

[84] V. Latora and M. Marchiori , “Efficient Behavior of Small‐World Networks,” Physical Review Letters 87 (2001): 198701. 11690461 10.1103/PhysRevLett.87.198701

[85] E. Bullmore and O. Sporns , “The Economy of Brain Network Organization,” Nature Reviews Neuroscience 13 (2012): 336–349. 22498897 10.1038/nrn3214

[86] D. S. Bassett and E. Bullmore , “Small‐World Brain Networks,” The Neuroscientist 12 (2006): 512–523. 17079517 10.1177/1073858406293182

[87] O. Sporns and J. D. Zwi , “The Small World of the Cerebral Cortex,” Neuroinformatics 2 (2004): 145–162. 15319512 10.1385/NI:2:2:145

[88] P. Mougkogiannis and A. Adamatzky , “Polymorphism in Glu‐Phe‐Asp Proteinoids,” Biomimetics 10 (2025): 360. 40558329 10.3390/biomimetics10060360 PMC12190980

[89] P. Mougkogiannis and A. Adamatzky , “Electroactive Proteinoid–Quantum Dot Systems,” Small Science 5 (2025): e202500418. 41395513 10.1002/smsc.202500418 PMC12697817

[90] B. Fischl , N. Rajendran , E. Busa , et al., “Cortical Folding Patterns and Predicting Cytoarchitecture,” Cerebral Cortex 18 (2008): 1973–1980. 18079129 10.1093/cercor/bhm225 PMC2474454

[91] P. Mougkogiannis and A. Adamatzky , “Optical Recognition of the English Alphabet Using Proteinoids,” ACS Omega 9 (2024): 51098–51119. 39758676 10.1021/acsomega.4c06401 PMC11696383

[92] P. Mougkogiannis and A. Adamatzky , “Serotonergic Mechanisms in Proteinoid‐Based Protocells,” ACS Chemical Neuroscience 16 (2025): 519–542. 39840997 10.1021/acschemneuro.4c00801 PMC11803625

[93] S. Achard , R. Salvador , B. Whitcher , J. Suckling , and E. Bullmore , “A Resilient, Lowfrequency, Small‐World Human Brain Functional Network with Highly Connected Association Cortical Hubs,” Journal of Neuroscience 26 (2006): 63–72. 16399673 10.1523/JNEUROSCI.3874-05.2006 PMC6674299

[94] L. R. Varshney , B. L. Chen , E. Paniagua , D. H. Hall , and D. B. Chklovskii , “Structural Properties of the Caenorhabditis Elegans Neuronal Network,” PLoS Computational Biology 7 (2011): e1001066. 21304930 10.1371/journal.pcbi.1001066 PMC3033362

[95] O. Sporns , “Small‐World Connectivity, Motif Composition, and Complexity of Fractal Neuronal Connections,” Bio Systems 85 (2006): 55–64. 10.1016/j.biosystems.2006.02.008 16757100

[96] D. S. Bassett , N. F. Wymbs , M. A. Porter , P. J. Mucha , J. M. Carlson , and S. T. Grafton , “Dynamic Reconfiguration of Human Brain Networks during Learning,” Proceedings of the National Academy of Sciences 108 (2011): 7641–7646. 10.1073/pnas.1018985108 PMC3088578 21502525

